# Tautomeric equilibrium, proton affinity and mass spectrometry fragmentation of flexible hydrogen-bonded precursors and rigid $\hbox {N}\longrightarrow \hbox {BF}_2$ fluorescent dyes

**DOI:** 10.1038/s41598-021-94978-9

**Published:** 2021-08-06

**Authors:** Małgorzata A. Kaczorowska, Anna Kaczmarek-Kędziera, Borys Ośmiałowski

**Affiliations:** 1 Faculty of Chemical Technology and Engineering, UTP University of Science and Technology, Seminaryjna 3, 85-326 Bydgoszcz, Poland; 2 Faculty of Chemistry, Nicolaus Copernicus University in Torun, Gagarina 7, 87–100 Toruń, Poland

**Keywords:** Physical chemistry, Theoretical chemistry

## Abstract

The stability of two groups of conformationally locked molecules, similar in topology, but differing only by the type of the bridge rigidifying their structure, is studied. The series of the less-rigid 2-phenacylheterocyclic compounds and their stiff difluoroboranyl derivatives are investigated for the determination of the effect of $\hbox {NCH}_3$/S/O replacement in a five-membered heterocyclic ring and the presence of a strong electron-donating group on the tautomeric equilibrium, protonation affinity, and fragmentation pattern observed in the structural elucidation by means of mass spectrometry technique. The results of the $\omega$B97X-D/6-311++G(d,p) calculations, the topological analysis of electron density as well as the experimental MS measurements show the importance of the number of heteroatoms, their properties, and location in the molecule for the rational design of the systems of desired stable tautomers or the favorable protonation sites. The obtained data allow for the understanding of the fundamentals of the novel highly fluorescent difluoroborates fragmentation behavior, vital for their structural elucidation with the application of high-resolution tandem mass spectrometry methods.

## Introduction

Dyes containing a disubstituted boron center constitute a well-known class of fluorophores, applied in various branches of science and technology, such as biological labeling^1,2^, construction of new drug carriers, anticancer or antimicrobial photosensitizing agents^3,4^ or dye-sensitized solar cells^5,6^. They owe the wide popularity to their extraordinary properties, namely large fluorescence quantum yield and easy chemical modifications. The rational design, and further, the precise tuning of these luminophore characteristics is vital and can be achieved as a consequence of the good understanding of the relationships between the molecular structure and their optical features^7–10^. However, even small structural modification can significantly affect the functional properties and, consequently, the applicability of dyes^11^. Moreover, the advanced process of the molecular design is usually multidimensional and influence of several factors at the same time can undergo the synergistic or anti-cooperative combination. This fact rationalizes the requirement of application of computational chemistry tools in the initial step of the procedure.

In order to verify the properties of the theoretically designed fluorophores, the synthesis of the selected species can be performed. Thus, in the context of the present study, it is also crucial to control precisely the difluoroboranyl derivatives synthesis processes, which often requires confirmation of the structure, properties and—what follows—the reactivity of the precursors and intermediate products. The precursors for these system synthesis are known to exhibit relatively high flexibility due to the presence of several single carbon-carbon bonds, enabling free rotation. For a group of the investigated precursors, such a rotation can strongly affect their rigidity by shifting the hydrogen bond donor towards the hydrogen bond acceptor. This transformation can lead to the formation of the intramolecular hydrogen bond which considerably stabilizes the molecule. In the six-membered quasi-rings formed by the hydrogen bonding, the particular strengthening of this intramolecular interaction has been observed and ascribed to the so-called resonance-assisted hydrogen bond^12–14^. The subtle balance between conformers, hydrogen bonding and tautomeric equilibria delivers the structures characterized by the varying electronic structure and thus, in their proton affinity. This, on the other hand, can strongly influence either the $\pi$-electron delocalization, aromatic character, reactivity, preferring nucleophilic or electrophilic reagents, sensitivity for the red-ox processes, bond energy and thus the fragmentation patterns in the mass spectrometry technique^15,16^.

The instrumental analysis of the strucutre of molecules in solution is usually achieved by NMR techniques. The same technique, however, does not deliver direct information on system stability. For that purpose the mass spectrometry may be helpful, being the technique which allow drawing additional information.

Over the last decade, the combination of electrospray ionization high resolution mass spectrometry (ESI-HRMS) and tandem mass spectrometry (MS/MS) methods has emerged as an important tool in the structural characterization of different classes of dyes^17^. ESI (high voltage, atmospheric pressure) enables most of the small molecules analyzed to be transferred directly from the solvent into the mass spectrometer without changing their structures^18,19^. HRMS offers high mass accuracy and sensitivity, which are necessary for the accurate determination of the elemental composition and charge of the ions generated^20^. Tandem mass spectrometry methods (MS/MS), such as Collision Induced Dissociation (CID, based on inelastic collisions of ions with inert gases)^21^ or Higher Energy Collisional Dissociation, also called a beam type CID^22^ provide detailed information on the structure of the analyzed compounds. They have been used for the structural elucidation of different classes of dyes, including: cyanine dyes^23–25^, xanthenes^26^, water soluble azo-dyes^27^ and BODIPYs and their derivatives^17,28^. However, because of the dependence of the MS/MS fragmentation behavior of dyes on all structural motifs of the analyzed ions (the presence of specific functional groups, occurrence of heteroatoms, the charge of the examined species)^24,26,29^, the application of HCD technique as the essential analytical tool on a larger scale requires the establishing of general rules for the fragmentation of specific classes of dyes. Thus, the simultaneous analysis of the dyes and their precursors of similar topology is crucial for the development of methods and understanding of their structure-property relationship.

The structural modification of the $\hbox {BF}_2$-carrying fluorophores studied by us before were focused on the substituent effetcs, benzannulation, geometry of the amino group, $\pi$-electron conjugation, to mention a few^10^. The influence of the heterocyclic ring composition and the presence of the terminal dimethylamino group on the photophysical properties of the 2-phenacylheterocyclic precursors and their corresponding $\hbox {BF}_2$—bearing counterparts has been thoroughly investigated with the spectroscopic and theoretical approaches^10^. It has been proven that the presence of the –$\hbox {N}(\hbox {CH}_3)_2$ substituent significantly affects the position and the shape of the absorption and emission bands and strongly increases the fluorescence quantum yield.

**Figure 1:**
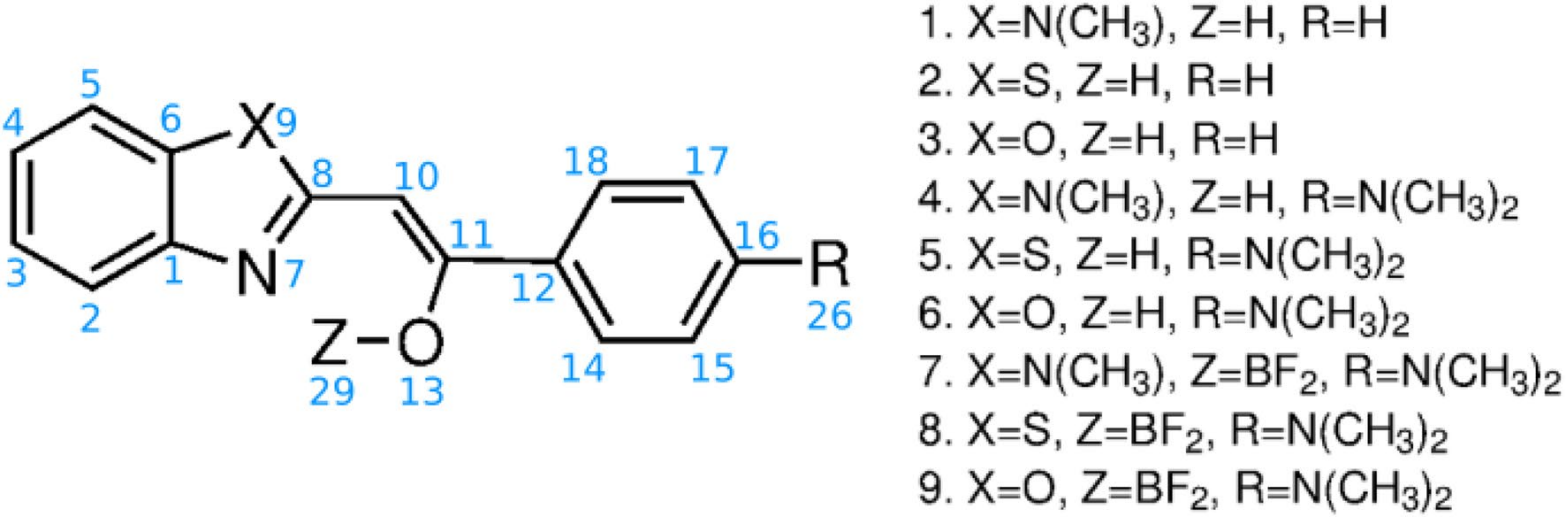
Structures of analyzed compounds (in blue—atom numbering).

The present study provides the extension of this previous report to the field of the reactivity of the precursors and the comparison of the influence of the two intramolecular stabilizing interactions, namely hydrogen bond and $\hbox {N}\longrightarrow \hbox {BF}_2$ interaction. The structures of the analyzed compounds are shown at Fig. 1. The main purpose of this research is to conduct a fundamental study on the influence of N($\hbox {CH}_3$)/S/O replacement in a five-membered ring and the presence of additional strong electron-donating terminal –$\hbox {N}(\hbox {CH}_3)_2$ group on the gas-phase tautomeric equilibria, protonation sites and fragmentation process of a series of 2-phenacylheterocycles stabilized by intramolecular hydrogen bonding and Lewis-acid/Lewis-base interaction.

## Experimental and theoretical methods

### Computational methodology

Full geometry optimization has been performed for all of the investigated species with a long-range corrected hybrid functional containing an empirical Grimme D2 dispersion correction^30^, $\omega$B97X-D^31^, together with a conventional Pople basis set augmented with diffuse functions, 6-311++G(d,p)^32–34^, in vacuum. This has been proven to provide a safe general-purpose approach for prediction of molecular geometries, tautomeric equilibria and reaction barrier heights and delivers the data for comparison with the MS experiment^35–37^. The tautomeric equilibria were also investigated with two other multipurpose hybrid functionals, namely the most popular three-parameter B3LYP^38^ one and the Truhlar M06-2X^39^ Minnesota functional containing 54% of exact exchange, recommended for the main group thermochemistry and non-covalent interactions^39,40^. As the wave-function formalism reference, also the Hartree–Fock^41^ and MP2^42–45^ results are provided in SI. They are compared with the double hybrid functional B2PLYP containing the combination of the exact HF exchange and MP2-like correlation contribution, additionally supplemented with the empirical Grimme dispersion D3 correction. Such functionals are perceived as the significant improvement upon the conventional hybrid functionals in context of dispersion intramolecular contribution description for relative energies or weak intermolecular interactions^46–48^. Additionally, as the reference, the single-point energy calculations for the investigated tautomers has been performed within the high-level local correlation approach in the domain-based local pair natural orbitals coupled cluster version, namely DLPNO-CCSD(T), providing linear scalling vital for high accuracy calculations for large systems^49–53^. The B2PLYP-D/6-311++G(d,p) and DLPNO-CCSD(T)/aug-cc-pVTZ results are presented in Fig. S4.

The character of the stationary points has been confirmed with harmonic vibrational analysis. The reaction paths for proton transfer have been verified with the intrinsic reaction coordinate calculations.

Proton affinities (PA) and gas phase basicities (GB) have been estimated for the model protonation reaction, acid [**X**+H]$^+$ $\longrightarrow$ base [**X**] + $\hbox {H}^+$, as the enthalpy and Gibbs free energy components, respectively:

1 $$\begin{aligned} {\mathrm{PA}}= & \, \Delta H_{base [{\mathbf {X}}]}+\Delta H_{H^+}-\Delta H_{acid [{\mathbf {X}}+H]^+}, \end{aligned}$$

2 $$\begin{aligned} {\mathrm{GB}}= &\, \Delta G_{base [{\mathbf {X}}]}+\Delta G_{H^+}-\Delta G_{acid [{\mathbf {X}}+H]^+}, \end{aligned}$$

where **X** denotes one of the analyzed systems, $\Delta H$ is the enthalpy change, calculated as the sum of electronic energy and thermal correction to enthalpy at 298.15 K and $\Delta G$ is a Gibbs free energy change. The enthalpy of the proton at 298.15 K was assumed to be equal to 5/2RT = 0.002368 Hartree, and its Gibbs free energy − 0.0100 Hartree^54^. It has been previously shown, that the time-efficient DFT approaches provide the reliable values of proton affinity for a wide spectrum of organic moieties^54–58^, therefore the $\omega$B97X-D/6-311++G(d,p) approach has been applied for PAs and GBs as well. Corresponding Wiberg bond indexes are presented. The calculations have been performed in Gaussian16 and Gaussian09 packages^59,60^ and the DLPNO-CCSD(T) calculations have been carried out in ORCA 4.2.1^61,62^.

In order to shed more light on the protonation and fragmentation processes in the investigated systems, the Atom-In-Molecules analysis has been performed within AIMAll program^63–65^. The obtained electron density $\rho$, laplacian of electron density $\bigtriangledown ^2 \rho$, total energy density $H_b$^65^ and the estimation of the energy of the intermolecular hydrogen bonds calculated according to Espinosa et al.^66^ as $E_{HB}=0.5\cdot$V, where V is the potential energy density in bond critical point, are summarized in Supplementary Information.

### Chemicals

All the reagents and solvents necessary for the synthesis were purchased from Sigma-Aldrich and were used without further purification. The methods applied for the synthesis of 2-phenacylheterocycles and their difluoroboranyl derivatives (shown at Fig. 1) have been described in detail elsewhere^10^.

### Mass spectrometry experiments

The Q-Exactive Orbitrap mass spectrometer (Thermo Fisher Scientific, Bremen, Germany) has been used to perform all of the mass spectrometry experiments. The examined compounds were dissolved in methanol and formic acid solution (98:2, vol/vol) before being introduced into the mass spectrometer using an electrospray source (Advion BioSciences ltd., Ithaca, NY, USA). Data acquisition and analysis were performed using the Thermo Xcalibur software (ver. 4.1.31.9). All of the mass spectra were obtained in positive ion mode within the m/z range of 50–750, at the resolution of 140,000 (m/z 200). MS/MS experiments were carried out with the use of higher energy collisional dissociation mode (HCD) with normalized collision energy set individually for each compound (usually in a range of 15–45 eV). The resulting mass spectra were analyzed manually.

## Results and discussion

### Tautomeric equilibria

**Figure 2:**
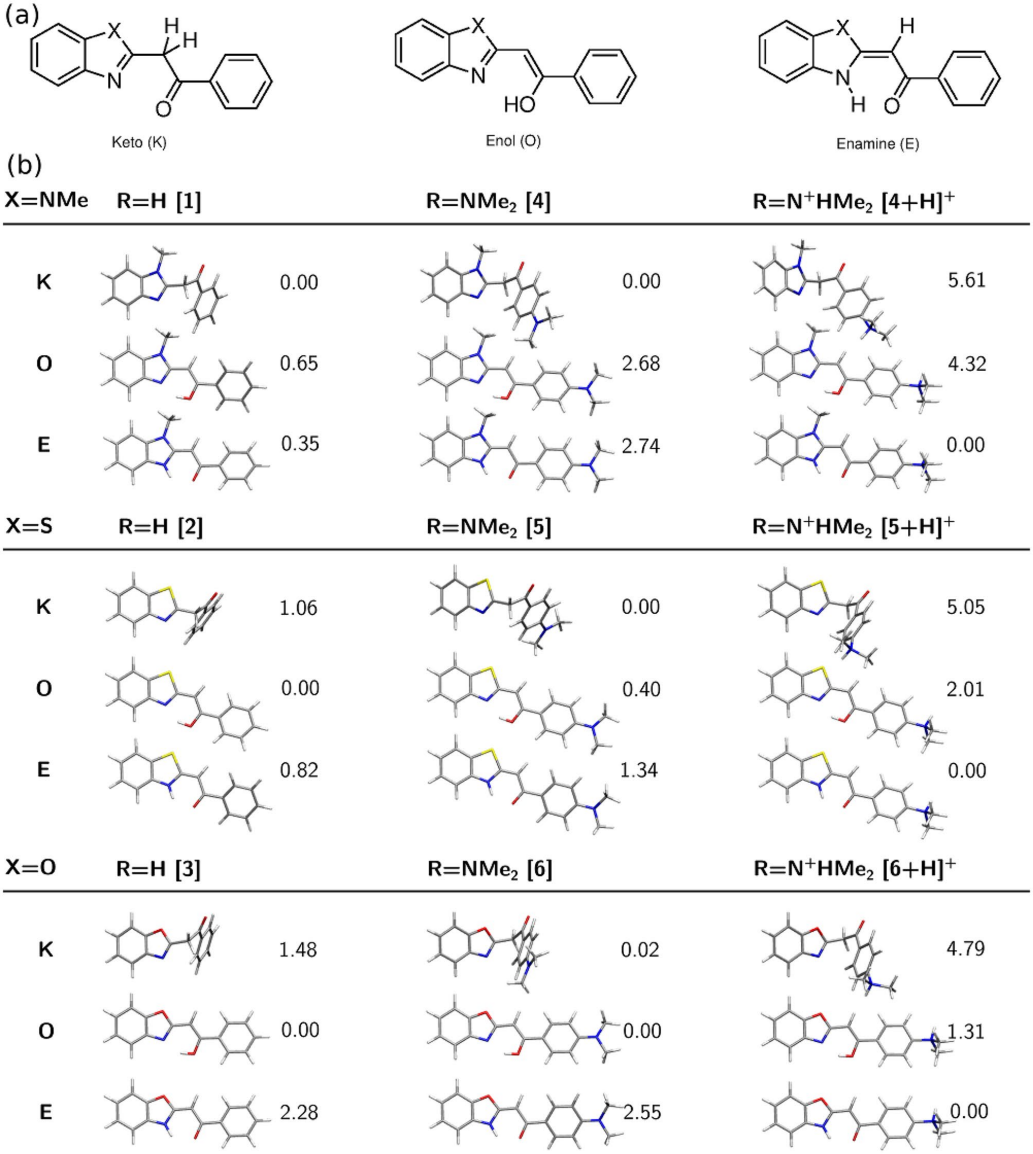
Tautomeric equilibria for neutral **1**–**6** (panel (**a**): K, O and E denote keto, enol and enamine form, respectively) and dimethyloamine-protonated counterparts of **4**–**6**. $\omega$B97X-D/6-311++G(d,p) relative energies in panel (**b**) are given in kcal/mol.

The initial part of the computational study is devoted to the tautomeric equilibria in neutral **1**–**6** species and is focused on the determination of the stability order of the tautomers and thus presume about the composition of the reaction mixture upon the standard conditions. In the present study, **1**–**3** series (R=H) allow for the analysis of the effect of the nature of the heterocyclic five-membered ring on the tautomeric equilibrium, while **4**–**6** (R=$\hbox {N}(\hbox {CH}_3)_2$) gives the information about the influence of the electron-donating dimethylamino group. On the other hand, the protonation of the terminal dimethylamino group, which occurs spontaneously for instance in the process of ESI-HRMS analysis, provides the strong electron-withdrawing effect on the molecule under investigation. Therefore, Fig. 2 presents the relative energies of keto (K), enol (O) and enamine (E) tautomers of **1**–**6** and $[{\mathbf{4}}\hbox {+H}]^{+}$, $[{\mathbf{5}}\hbox {+H}]^{+}$ and $[{\mathbf{6}}\hbox {+H}]^{+}$ systems with protonated dimethylamino group. The full set of relative energies and relative Gibbs free energies for the three tautomers of nine systems obtained in different methods are provided in Supplementary Information as histograms in Fig. S4 and S5. One should also notice that for the comparison for all of the investigated structures the alike keto tautomers have been selected—all of them are significantly bend and possess the carbonyl group pointing up to the direction of the N($\hbox {CH}_{{3}}$)/S/O atom in heterocyclic ring. Consequently, for all of the analyzed phenacylheterocycles and their $\hbox {N}(\hbox {CH}_3)_2$-derivatives this is the lowest energy keto tautomer. Additionally, at least two other rotamers of the keto form can be determined: with carbonyl group pointing downwards in the direction of the heterocyclic amine/imine nitrogen and in carbonyl placed in the plane perpendicular to the heterocyclic ring. All of these keto rotamers for **1**–**6** lie in the 5 kcal/mol energy interval. Enol and enamine tautomers for all of the 2-phenacylheterocycles and their dimethylaminated derivatives are considered as planar forms stabilized with the intramolecular $\hbox {N}\ldots \hbox {H--O}$ or $\hbox {N--H}\ldots \hbox {O}$ interaction.

Several general trends can be noticed. First, for R=H (systems **1**–**3**) the observed energy differences are smaller than 2.3 kcal/mol and for imidazole and thiazole remain below the accuracy of the applied computational methodology. Therefore, one can assume the easy N7-to-O13 proton transfer and significant abundance of all tautomers in the reaction mixture. The substitution of the investigated species with an electron-donating terminal $\hbox {N}(\hbox {CH}_3)_2$ group leads to the additional stabilization of the enol form in the case of **4** and **6**. The protonation of the terminal $\hbox {N}(\hbox {CH}_3)_2$ group significantly destabilizes the keto tautomer in all three investigated systems, thus causing its smallest abundance. Nevertheless, the obtained energy differences among tautomers are in most cases small enough to remain inconclusive. The qualitative tendencies of the $\omega$B97X-D/6-311++G(d,p) calculations are confirmed by the B2PLYP-D/6-311++G(d,p) and DLPNO-CCSD(T)/aug-cc-pVTZ data (Fig. S4).

The general remark that can be drawn from these results is that the reaction mixtures in standard conditions contain multitude of low energy isomers for all analyzed systems. However, it should be underlined that due to the MS protocol applied, ab initio calculations presented here are performed in vacuum in order to achieve the closest correspondence between the experimental MS setup and computational study. This, however, can significantly alter the results with respect to other experimental measurements carried out in solution. Therefore, the direct comparison of the experimental data with the present results is in principle not straightforward taking into account comparisons between MS and NMR techniques. It should be mentioned that on the basis of the $^{1}\hbox {H}$ and $^{13}\hbox {C}$ NMR measurements in $\hbox {CDCl}_3$ and computational approach^67^ of a series of similar 1-methyl-2-phenacylbenzimidazole systems, enamine tautomer is the least stable and therefore not present in the reaction mixture. On the other hand keto has been found to remain the most stable tautomer independently on the substituent present in the phenyl ring, and enol form have been shown to undergo an additional stabilizing effect from the intramolecular $\hbox {N}\ldots \hbox {H-O}$ interaction. The K and O energetic order is reversed in the case of corresponding oxazole derivatives^68^.

### 2-phenacylheterocycles

#### Proton affinity of **1**–**3**

**Figure 3:**
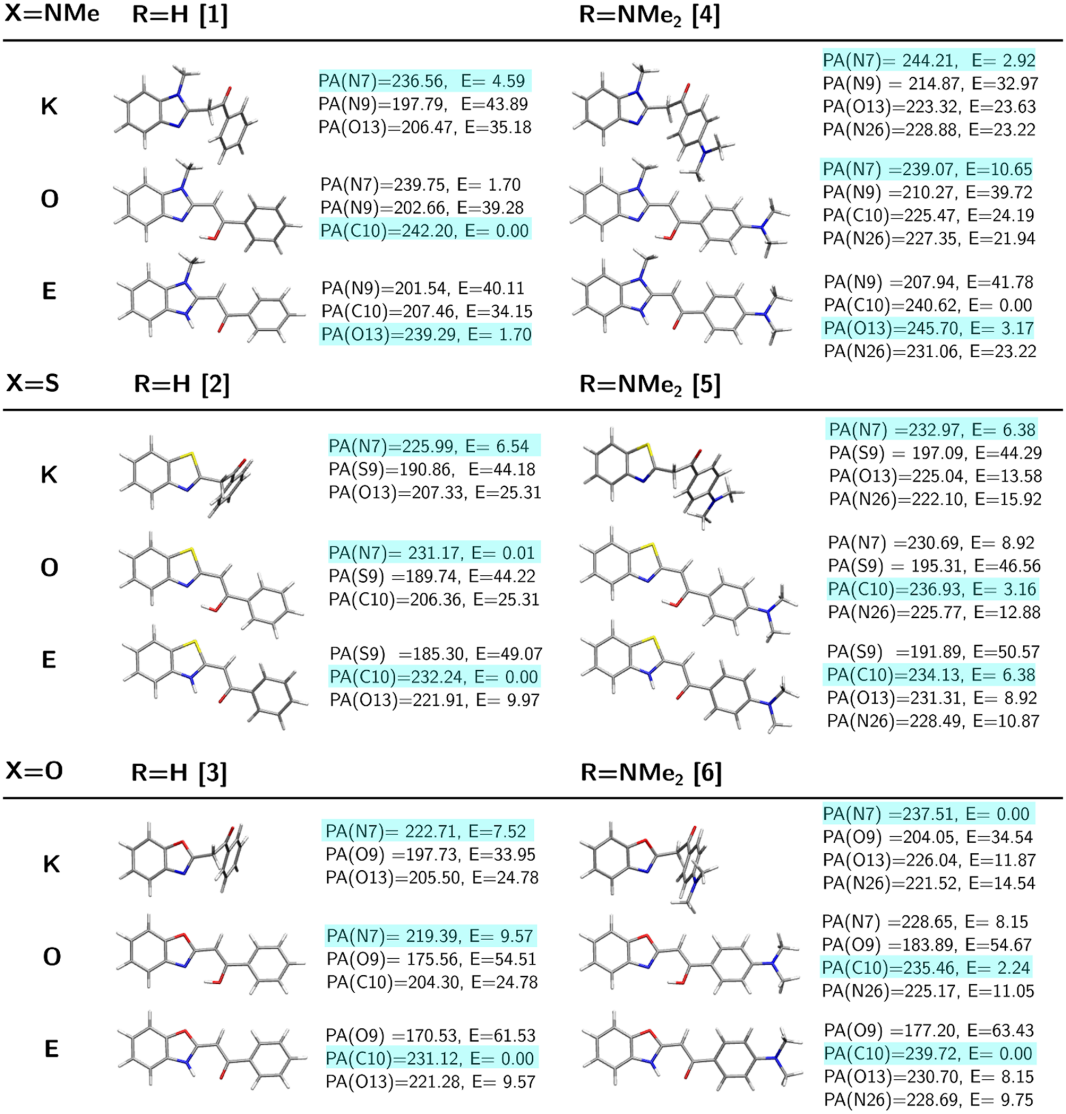
Proton affinities for **1**–**6** tautomers (K, O and E denote keto, enol and enamine form, respectively). PA(N) is the proton affinity at N7 protonation site, PA(O13) denotes the protonation at O13 site, PA(C10) stands for the proton affinity at C10 site, PA(X9) corresponds to the proton affinity at X9 site (where X=N,S,O) and PA(N26) is the proton affinity at the terminal dimethylamino group (in kcal/mol; definition given in text). E is the $\omega$B97X-D/6-311++G(d,p) relative energy for protonated forms, in kcal/mol.

Proton affinity values for the 2-phenacylheterocyclic compounds as well as their –$\hbox {N}(\hbox {CH}_3)_2$-substituted derivatives are presented in Fig. 3. Additionally, the gas-phase basicity for all these systems is provided in Table S10. Both of these notions, as well as the simple energetic criteria, provide the alike informations for the preferred protonation sites in **1**–**3**. The generally observed tendencies point out that in the neutral systems, the sites most prone to protonation are the N7 atom in heterocyclic ring and the C10 atom. The only system which seem to favour the O13 protonation is the enamine form of imidazole. However, one should also notice, that the obtained PA and GB values can be very similar for several protonation sites (for instance PA equal to 239.75 and 242.20 kcal/mol for enolic form of **1** at N7 and C10 sites, respectively), thus not allowing for the definite conclusions about the single prevailing protonated form and—what follows—indicating the probable presence of several protonated forms of similar stability in the experimental conditions. The more detailed discussion of the numerical values of the PA and GB for **1**–**3**, the Wiberg bond indexes and feasible fragmentation mechanisms is provided in SI.

**Figure 4:**
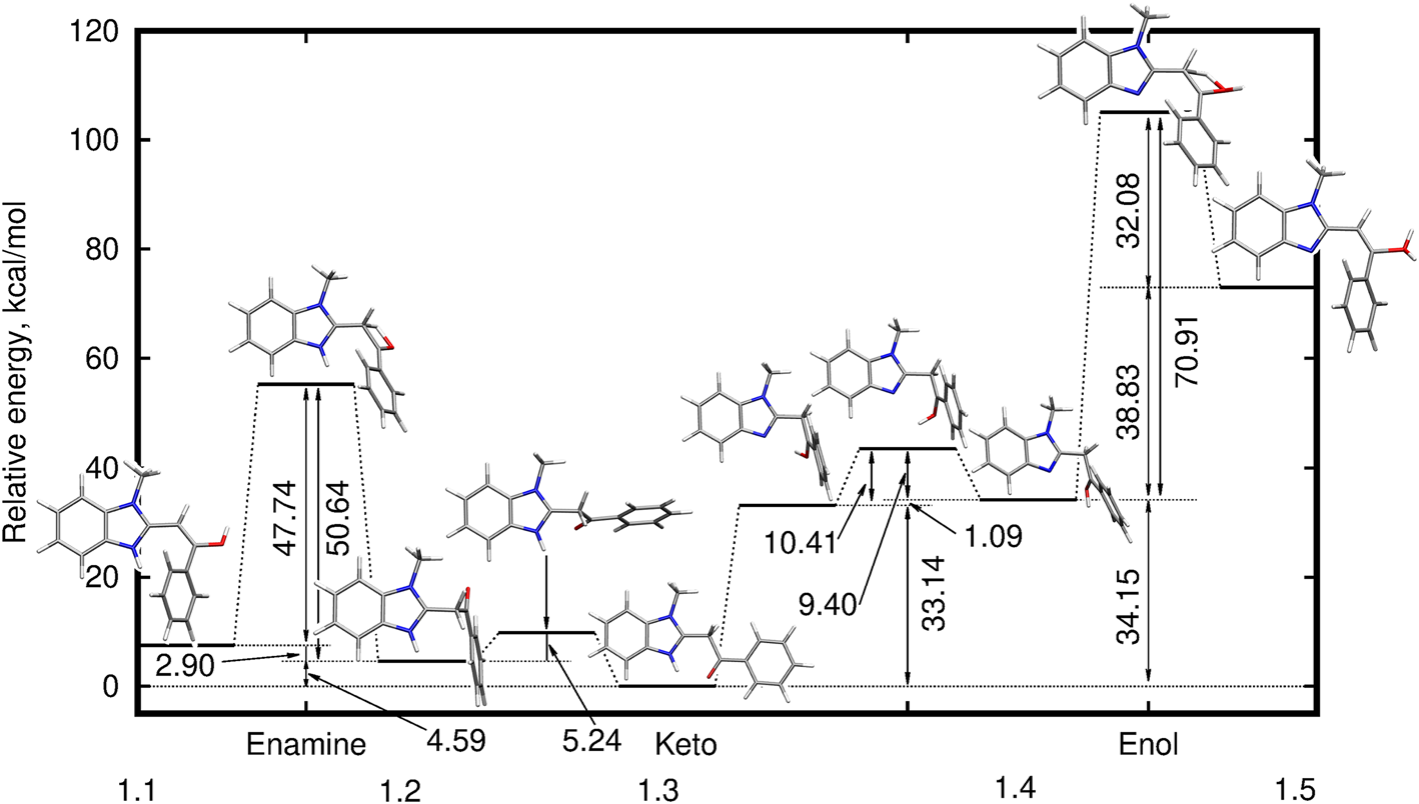
Mechanism of possible proton transfer processes in $[{\mathbf{1}}\hbox {+H}]^{+}$ ion calculated within the $\omega$B97X-D/6-311++G(d,p) approach.

In order to shed the light on the fragmentation pattern, the computational analysis of the mechanism of the possible proton transfer pathways in [**1**+H]$^+$ has been performed and depicted in Fig. 4. The estimated energy barriers demonstrate that starting from the lowest-energy 1.3 keto tautomer of **1** protonated at the C10 site, the process in two directions can proceed: the lower energy barrier lead to the intramolecular rotation and later on, the C10–O13 proton transfer to the enamine form 1.1, with a barrier of 50.64 kcal/mol. The other possibility is the generation of the protonated enolic form 1.5, which however requires crossing of the high (over 100 kcal/mol) overall energy barriers. This would be surely impossible for the spontaneous process in standard conditions, nevertheless taking into account the conditions under which HCD experiments are carried out, it remains viable during the fragmentation process.

The Atom-In-Molecules analysis allows the estimation of the intramolecular hydrogen bond energy, $E_{HB}$, according to Espinosa et al.^66^ The corresponding data for enol and enamine forms of **1**–**6** and their protonated counterparts are summarized in Table S13 and on histograms in Fig. S9. One can clearly notice the substantial differences in the strength of this interaction depending on the system and its tautomer. Enol form is significantly favoured, exhibiting the strongest stabilizing effect of H-bond, however its strength diminishes in the sequence of N($\hbox {CH}_3$)/S/O in heterocyclic ring in both series (namely pristine **1**–**3** and dimethylaminated **4**–**6**). Similar dependence on the heterocyclic ring composition is presented by the intramolecular hydrogen bond in the enolic form protonated at the terminal dimethylamine group.

#### Fragmentation process of **1**–**3**

**Figure 5:**
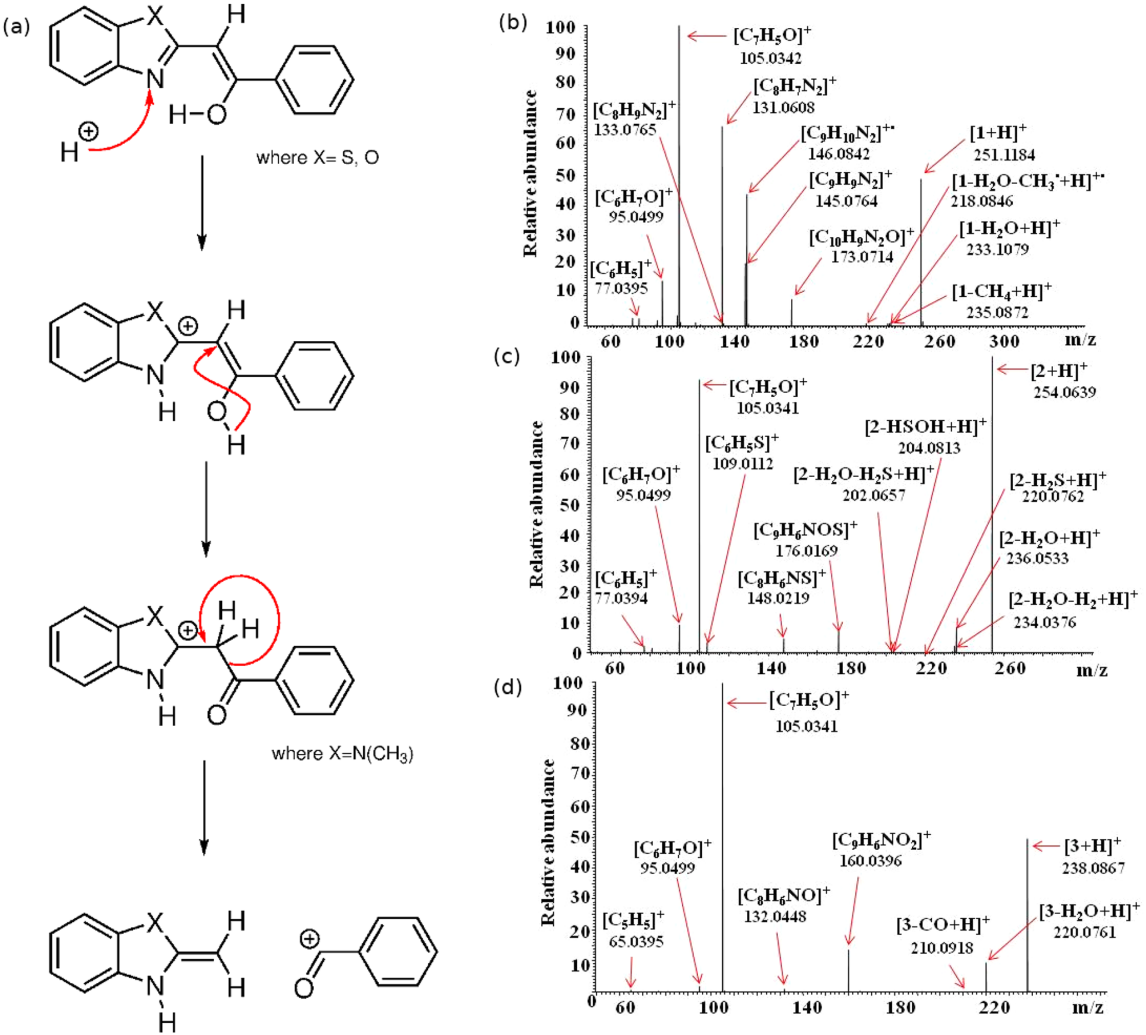
(**a**) Proposed ESI HCD MS/MS fragmentation mechanism of protonated compounds **1**-**3** leading to formation of [$\hbox {C}_{{7}} \hbox {H}_{{5}}$O]$^{+}$ ions, ESI HCD MS/MS mass spectra of singly charged ions $[{\mathbf{1}}\hbox {+H}]^{+}$ (**b**), $[{\mathbf{2}}\hbox {+H}]^{+}$ (**c**) and $[{\mathbf{3}}\hbox {+H}]^{+}$ (**d**). The values given under the molecular formulas correspond to the experimental masses of the generated products ions.

The results of the DFT calculations show that in the case of compounds **1**–**3**, heterocyclic nitrogen atoms (–N=) are the privileged protonation sites (compare Fig. 5, panel (a)). The weakest bonds according to the calculated Wiberg bond indexes (see Table S11), most susceptible for breaking upon the MS collisions, include the detachment of the methyl group from the imidazole ring (N9–C30), and (depending on the protonation site) the C10–C11 or C11–O13 bonds. The ESI HCD MS/MS mass spectra of the three ions $[{\mathbf{1}}\hbox {+H}]^{+}$, $[{\mathbf{2}}\hbox {+H}]^{+}$ and $[{\mathbf{3}}\hbox {+H}]^{+}$ are dominated by signals corresponding to singly charged parent ions and to the [$\hbox {C}_{{7}} \hbox {H}_{{5}}$O]$^{+}$ species (Fig. 5, panel (b)).

In the case of the [$\hbox {C}_{{7}} \hbox {H}_{{5}}$O]$^{+}$ products, different structures should be considered, i.e. [($\hbox {C}_{{6}} \hbox {H}_{{3}}$)-CH-OH]$^{+}$, [($\hbox {C}_{{6}} \hbox {H}_{{4}}$)-CH=O]$^{+}$ and [($\hbox {C}_{{6}} \hbox {H}_{{5}}$)-C=O]$^{+}$. Generating the [$\hbox {C}_{{7}} \hbox {H}_{{5}}$O]$^{+}$ species can be related to the protonation of –N= atoms of the precursors, as shown in panel (a) of Fig. 5. The mechanism of formation of [($\hbox {C}_{{6}} \hbox {H}_{{3}}$)-CH-OH]$^+$ and [($\hbox {C}_{{6}} \hbox {H}_{{4}}$)-CH=O]$^{+}$ ions would involve the complex intramolecular rearrangements, the detachment of hydrogen atoms from the benzene rings, cleavage of several bonds and is unlikely, whereas the mechanism leading to the formation of $\hbox {ArCO}^{+}$ products requires the transfer of only one proton and simple intramolecular rearrangement (in case of protonated keto tautomeric form of singly charged ions of **1** this mechanism is even simpler). Furthermore, also the analysis of previous results of gas-phase fragmentation behavior of similar structure compounds leads to the conclusion that formation of [($\hbox {C}_{{6}} \hbox {H}_{{5}}$)-C=O]$^{+}$ is most likely^69^.

The influence of the properties of the –N($\hbox {CH}_{{3}}$) group and single elements S and O, located in the five-membered ring on the higher energy collisional dissociation behavior of singly charged ions of **1**–**3** is observed in the case of minor HCD fragmentation processes. They are discussed in details in Supporting Information.

### Dimethylamino derivatives of 2-phenacylheterocycles

#### Proton affinitty of **4**–**6**

The substitution of the $\hbox {BF}_2$-containing fluorophores with the terminal $\hbox {N}(\hbox {CH}_3)_2$ group has been shown to drastically affect their photophysical properties due to the more pronounced charge transfer character of the $\hbox {S}_0\longrightarrow \hbox {S}_1$ transition^10^. In the case of their precursors however, although modifying the electron distribution within the molecule by dimethyloamine group can be observed, it seems to not influence much the protonation affinity and thus the ESI HCD MS/MS fragmentation pattern in the gas phase. Nevertheless, in most cases, the PA as well as the GB values seem to be slightly higher for the $\hbox {N}(\hbox {CH}_3)_2$-substituted 2-phenacylheterocycles than for their pristine analogs, what remains in agreement with chemical intuition (compare Fig. 3 and numerical values of GB in Table S10.).

**Figure 6:**
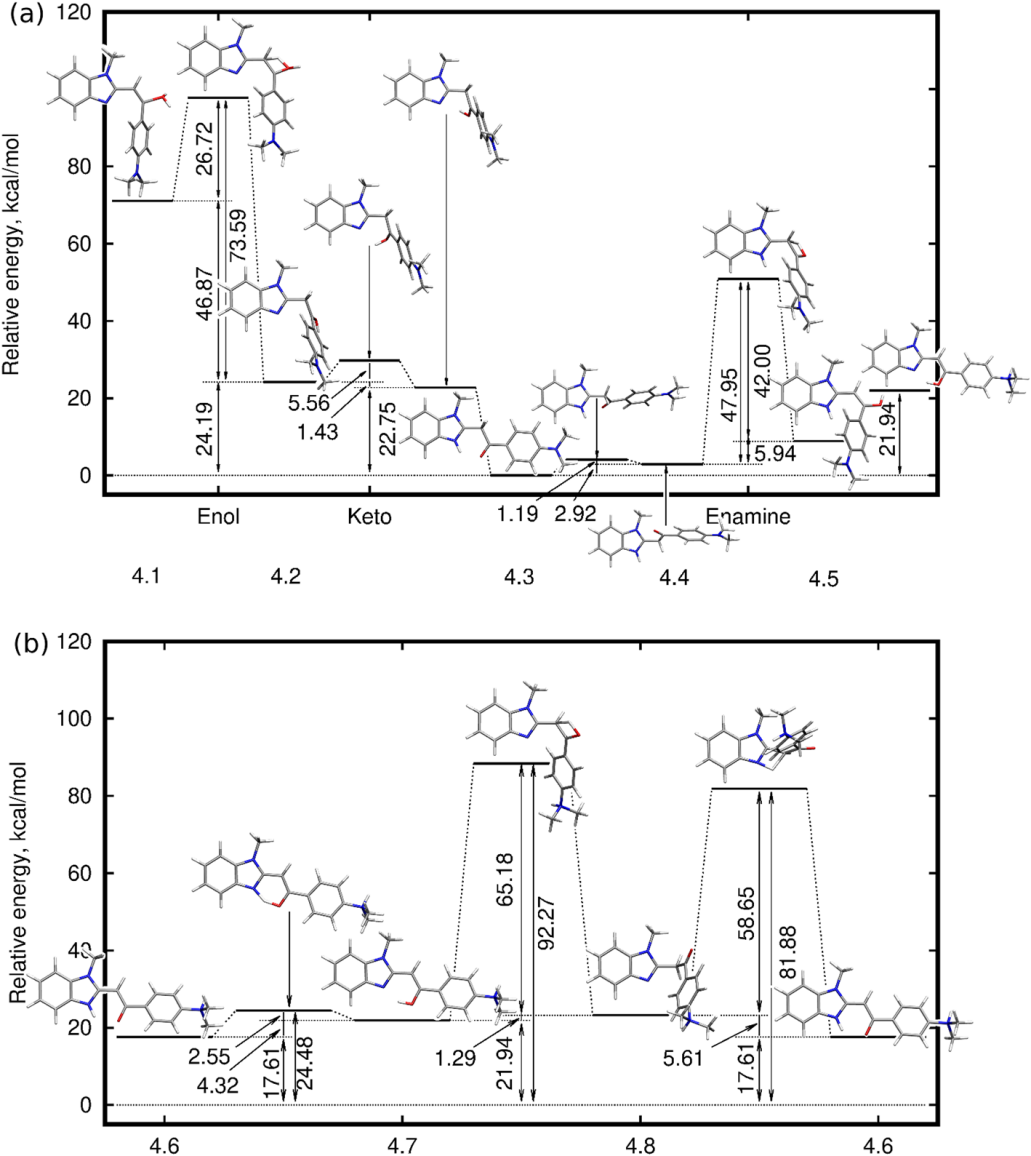
Mechanism of possible proton transfer processes in [**4**+H]$^+$ ion calculated within the $\omega$B97X-D/6-311++G(d,p) approach (**a**) protonation of the main part of the molecular core at N7, C10, O13; (**b**) protonation of the terminal dimethylamine substituent at N26. The relative energy for all the structures presented in both panels is given with respect to the same lowest energy 4.3 form bearing protons at N7 and C10.

Taking into account the distance between the heteroatoms from the molecular core (N7, N/S/O9 and O13) and in the terminal dimethylamino group, the intramolecular proton transfer between N26 and any other protonation site in the system arises to be improbable. Yet, the MS technique applied in the present study, provides the source of the external protons, which can be attached at any point. Therefore, two cases of the proton transfer process are considered here and they are presented as panel (a) and panel (b) of Fig. 6, for a mechanism not involving the terminal $\hbox {N}(\hbox {CH}_3)_2$ group and the other one, in which originally dimethylamino substituent is protonated. In the first path, as it was observed for **1**, the diprotonated hydroxyl group in 4.1 is the least stable and requires the highest energy barrier crossing. On the other hand, the proton transfer from the C10 of initial, most stable 4.3 keto form to O13, preceeded by the intramolcular rotation, delivers another relatively stable 4.5 isomer, which could possibly be observed in noticeable amounts even in standard conditions. Similar processes involving the proton transfer in the central part of the molecule, between the heterocyclic ring and enol group, for the system originally protonated at the terminal dimethylamino substituent, demand crossing of the significantly higher energy barrier (of the order of 60 kcal/mol) than in the case when both protons are placed close to each other. Still, taking into account the collision energy of MS/MS measurements, these processes cannot be definitely excluded from consideration of fragmentation pattern.

#### Fragmentation process of **4**–**6**

The introduction of the terminal dimethylamine group to the 2-phenacylheterocycle adds another possible protonation site, which occurs to be competitive with respect the C10, O13 or N/S/O9 atoms (Fig. 3, Table S10). The calculated Wiberg bond indexes (Tab. S14) indicate that in addition to the weakest points in the systems observed for **1**–**3** series, the detachment of $\hbox {CH}_3$ groups from the terminal dimethylamino substituent could be considered. In the case of the protonation of the nitrogen from the terminal group, also the detachment of the whole –$\hbox {N}(\hbox {CH}_3)_2$ substituent becomes plausible since the corresponding Wiberg bond indexes for C16-N26 bond remain smaller than 1.0.

**Figure 7:**
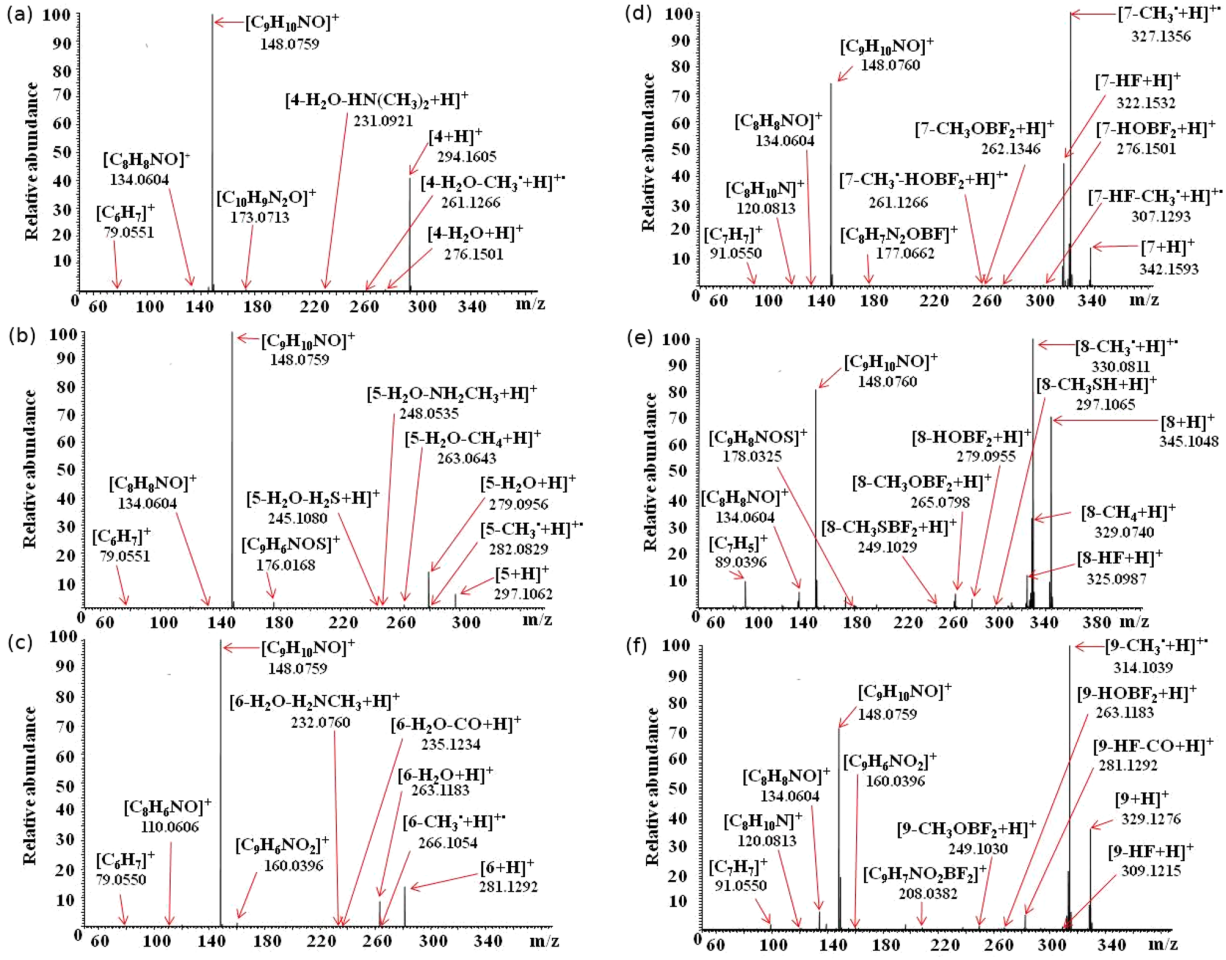
ESI HCD MS/MS mass spectra of singly charged ions $[{\mathbf{4}}\hbox {+H}]^{+}$ (**a**), $[{\mathbf{5}}\hbox {+H}]^{+}$ (**b**), $[{\mathbf{6}}\hbox {+H}]^{+}$ (**c**), [**7**+H] (**d**), [**8**+H] (**e**) and [**9**+H] (**f**). The values given under the molecular formulas correspond to the experimental masses of the generated products ions.

The results of the performed tandem mass spectrometry experiments (Fig. 7) clearly demonstrate that the main dissociation processes of dimethylamino derivatives are similar to that found in the fragmentation of unmodified 2-phenacylheterocycles, and are related to protonation of heterocyclic ring nitrogen atoms. The HCD MS/MS spectra of singly charged ions of compounds **4**-**6** are dominated by signals which can be assigned to [$\hbox {C}_{{9}} \hbox {H}_{{10}}$NO]$^{+}$ ([($\hbox {CH}_{{3}}$)$_{2}$N($\hbox {C}_{{6}} \hbox {H}_{{4}}$)-C=O]$^{+}$) species, corresponding to [$\hbox {C}_{{7}} \hbox {H}_{{5}}$O]$^{+}$ ions formed in the HCD of protonated molecules **1**-**3**.

The higher energy collisional dissociation of all ions containing –N($\hbox {CH}_{{3}}$)$_{2}$ group is also associated with protonation of nitrogen atoms from these moieties and as a consequence elimination of the entire or part of a dimethylamino substituent occurs (i.e., [**4**-$\hbox {H}_{{2}}$O-HN($\hbox {CH}_{{3}}$)$_{2}$+H]$^{+}$, [**5**-$\hbox {H}_{{2}}$O-$\hbox {CH}_{{4}}$+H]$^{+}$, [**6**-$\hbox {H}_{{2}}$O-$\hbox {H}_{{2}}$N(CH)$_{3}$+H]$^{+}$, [**6**-$\hbox {H}_{{2}}$O-$\hbox {CH}_{3}^{\varvec{\cdot }}$+H]$^{+\varvec{\cdot }}$). Furthermore, the presence of the dimethylamino group affects other minor fragmentation processes, because the terminal –N($\hbox {CH}_{{3}}$)$_{2}$ modifies the electron distribution within the molecules. This effect strongly depends on the elemental composition of the heterocyclic moiety or, more precisely, on the properties of atoms/groups that are present in the ring (N($\hbox {CH}_{{3}}$), S, O) and is discussed in details in Supplementary Information.

### Difluoroborates

#### Proton affinity of **7**–**9**

**Figure 8:**
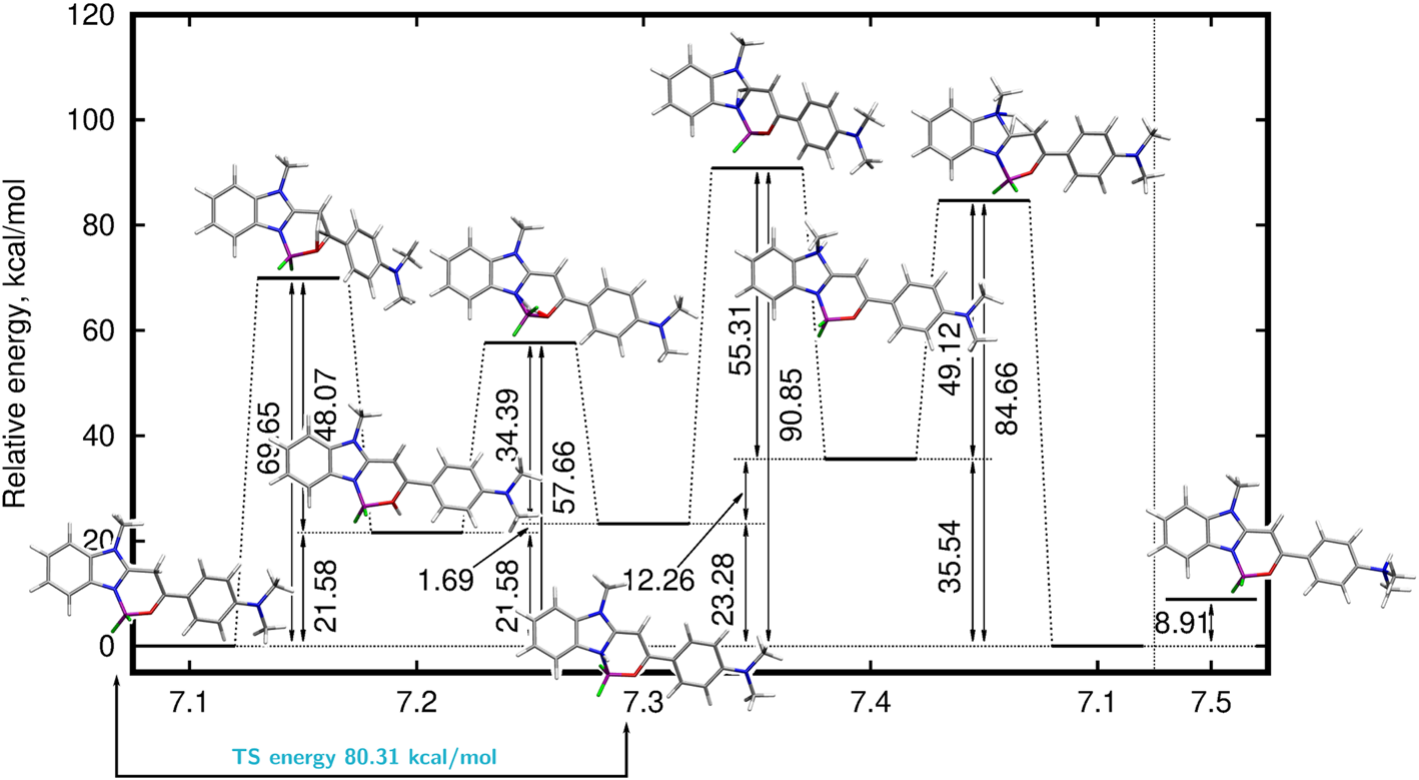
Mechanism of possible proton transfer processes in [**7**+H]$^{+}$ ion calculated within the $\omega$B97X-D/6-311++G(d,p) approach (in blue transition state relative energy for proton transfer from N7 to C10 is given; corresponding figures for [**8**+H]$^{+}$ and [**9**+H]$^{+}$ provided in Fig. S7 and S8).

Due to their rigidity, difluoroborate systems analyzed in the present study appear computationally less demanding. Therefore only one neutral form for **7**, **8** and **9** has been determined and consequently protonated by the ESI proton in all possible protonation sites in order to establish the energetic preferences and favorable proton affinities for fragmentation process justification. It has been undoubtedly shown that the highest proton affinity is found for a C10 protonation in all systems (234.32 kcal/mol for **7**, 229.49 kcal/mol for **8** and 227.26 kcal/mol for **9**). In the case of imidazole (**7**) this leads to the lowest energy protonated isomer 7.1 (see Fig. 8). Thereafter, the proton can be transferred to the O13 site (7.2) crossing the barrier of 69.65 kcal/mol and generating the structure of the relative energy equal to 21.58 kcal/mol. The further proton transfer can occur to the N7 site via the smaller barrier of 36.08 kcal/mol to the 7.3 isomer. The proton relocation to N9 site requires the energy cost of 67.57 kcal/mol and the relatively high energy 7.4 form (35.54 kcal/mol) is obtained. Although not feasible by the straightforward intramolecular process, the external ESI proton can also attack the N26 site generating the 7.5 structure of the relative energy 8.91 kcal/mol. Again, whereas some of the above-mentioned barrier are prohibitively, they can be easily crossed assuming the high collision energy in ESI HCD MS/MS. Thus, one can expect a numerous proton transfer processes occurring in the investigated system, weakening instantaneously different bonds in the molecule and allowing for the complex fragmentation processes. The careful analysis of the Wiberg bond indexes for **7** favors the destruction of the part of the structure containing $\hbox {BF}_{{2}}$ unit, since the O13-B29 (Wiberg bond index for the C10-protonated structure 0.5574), N7-B29 (0.6050) and B-F bonds (0.7799 and 0.7612) are the weakest in the whole system.

The modification of the heterocyclic ring composition from imidazole in **7** to thiazole in **8** does affect much neither the structure of the neutral form of the system nor the sequence of the protonation affinities, still for C10 protonation affinity being the largest (229.49 kcal/mol), followed by the $\hbox {N}(\hbox {CH}_3)_2$ PA (222.55 kcal/mol), fluorine site protonation with the HF detachment (211.29 kcal/mol), O13 protonation (PA equal to 207.13 kcal/mol), N7 protonation (PA 206.30 kcal/mol) and finally—the least readily protonated is the S9 site (184.21 kcal/mol). This PA’s ordering agrees with the energetic sequence of the protonated forms (compare Supplementary Information). The protonation affinity order for **9** directly follows the sequence observed both for **7** and **8**. The detailed discussion of the protonation affinity at different sites together with the Wiberg bond indexes analysis and energy barriers for protion transfer for **7**–**9** is provided in SI.

#### Fragmentation process of **7**–**9**

The gas-phase fragmentation behavior of difluoroborates (the structures **7**–**9** at Fig. 1) is different, compared with the dissociation of the corresponding 2-phenacylheterocycles and their dimethylamino derivatives. The HCD MS/MS spectra of all three ions [**7**+H]$^{+}$, [**8**+H]$^{+}$ and [**9**+H]$^{+}$ are dominated by signals corresponding to products formed as a result of loss of methyl radicals from the parent ions (Fig. 7). It should be emphasized that, although the process of elimination of a methyl radical was observed in the case of gas-phase fragmentation of various BODIPYs molecules^17^ and dimethylamino derivatives of 2-phenacylheterocycles, it was not a privileged/dominant process, which is consistent with the even electron rule. In general, exceptions to this rule are rare but have been reported for conjugated structures and compounds containing aromatic rings^70^. The formation of [**X**-$\hbox {CH}_{3}^{\varvec{\cdot }}$+H]$^{+\varvec{\cdot }}$ products (where **X**=**7**, **8**, **9**), regardless of the structure of the five-membered heterocyclic ring of the studied molecules, indicates that the methyl radicals originate from the dimethylamino groups. Furthermore, because the studied difluoroborates **7**–**9** differ from the corresponding **4**–**6** compounds only by the presence of the $\hbox {BF}_{{2}}$ group, the difference in HCD fragmentation behavior must be related to the properties of boron and/or fluorine atoms. The thesis can be supported by the results of a theoretical study of the influence of the boron atom and –$\hbox {BF}_{{2}}$ group on the acidity of alcohols (i.e., $\hbox {CH}_{{3}}\hbox {OH}$, $\hbox {BH}_{{2}} \hbox {CH}_{{2}}\hbox {OH}$, $\hbox {BF}_{{2}} \hbox {CH}_{{2}}\hbox {OH}$) in the gas phase, performed by Kheirjou et al.^71^. They reported that alcohol boronation considerably enhances its acidity, which can be related to the stabilization of the alkoxy ion due to the overlap of the unoccupied orbital of the boron atom with the electron pairs of the negative oxygen. The results of Natural Bond Orbital (NBO) analysis have shown that an increase in the acidity of the boron-containing alcohols is due to the charge transfer from the negative oxygen (in deprotonated structure) to the empty orbital of –$\hbox {BH}_{{2}}$ and –$\hbox {BF}_{{2}}$^71^. It could be assumed that in the case of the analyzed difluoroborates a similar phenomenon occurs. However, the results of DFT calculations performed for all compounds containing the $\hbox {BF}_{{2}}$ group (**7**-**9**) have shown, that in the most stable forms of ions the boron atoms are coordinated by heterocyclic nitrogen atoms. Consequently, these nitrogen atoms become a less favorable sites for the ionizing protons compared to (–N=) atoms of 2-phenacylheterocycles.

Fragmentation processes related to location of ionizing protons at (–N=) atoms of difluoroborates, leading to formation of [$\hbox {C}_{{9}} \hbox {H}_{{10}}\hbox {NO}$]$^{+}$ ions, occurs, but, are not the dominant dissociation reactions, as was the case with the dimethylamino derivatives of 2-phenacylheterocycles ([$\hbox {C}_{{9}} \hbox {H}_{{10}}\hbox {NO}$]$^{+}$ – major products in HCD of singly charged ions of **4**–**6**, analogous to dominant [$\hbox {C}_{{7}} \hbox {H}_{{5}}\hbox {O}$]$^{+}$ ions formed in HCD of protonated **1**–**3** molecules). Instead, the dominant dissociation processes of compounds **7**–**9** are associated with the protonation (ESI) of the nitrogen atoms of the dimethylamino groups and lead to the privileged loss of methyl radicals. This process is rationalized by the large values of the corresponding proton affinity and gas-phase basicity at the N26 centre (Tab. S17) and the Wiberg bond indexes for N26-C32 and N26-C36 bonds lower than 1.0 (Tab. S18).

More details on the secondary fragmentation processes of difluoroborates including the elimination of the HF, $\hbox {HOBF}_2$ or $\hbox {CH}_3 \hbox {SBF}_2$ moieties, is provided in SI.

## Summary and conclusions

The knowledge of the stability of fluorophores in the light of their structure is important not only in their excited state where all photoreactions may proceed, but also in the ground state. The most probable reaction in ground state of $\hbox {BF}_2$-carrying molecules is the loss of the $\hbox {BF}_2$ moiety leading to their precursors. Since those precursors exhibit tautomerism, their properties may, again, induce side reactions. Their knowledge and control can be vital for further utilization, and therefore a detailed information on the structural features of these systems with the emphasis on relative energies and stability of bonds is crucial. Based on those, one can even more precisely design compounds for special applications. In the light of the above, the structural changes introduced in the present study (H vs. $\hbox {NMe}_2$ and N vs. O vs. S) show how important are subtle modification for fragmentation.

All the theoretical calculations consistently support the mechanism of the fragmentation of 2-phenacylheterocycles as well as their difluoroborate counterparts, suggested on the basis of the experimental MS measurements. Wiberg bond indexes correlate well with the AIM electron density and together with the energy for the investigated reaction paths they rationalize the detachment of the benzoyl ion.

The structural elucidation of a set of 2-phenacylheterocycles which differ by the elements located in heterocyclic moieties ($\hbox {NCH}_{{3}}$/S/O), and the substituents attached to the benzene rings (presence/absence of –N($\hbox {CH}_{{3}}$)$_{2}$), as well as for their difluoroboranyl derivatives has been performed by means of ESI HCD MS/MS technique. For all of the analyzed ions, gas-phase charge-directed fragmentation processes strongly depend on the number and properties of heteroatoms. The formation of dominant HCD products of all 2-phenacylheterocycles with and without a dimethylamino groups is related to the location of ionizing proton(s) at the heterocyclic ring nitrogen atoms (–N=) (or at the carbon atoms from –CH– moieties, followed by intramolecular proton transfers). The presence of additional –N($\hbox {CH}_{{3}}$)$_{2}$ group bonded to the benzene ring causes the formation of minor fragmentation products associated with elimination of the entire or part of the dimethylamino substituent (i.e. [**4**-$\hbox {H}_{{2}}$O-HN($\hbox {CH}_{{3}}$)$_{2}$+H]$^{+}$, [**6**-$\hbox {H}_{{2}}$O-$\hbox {CH}_{3}^{\varvec{\cdot }}$+H]$^{+\varvec{\cdot }}$). It also has an effect on other, minor fragmentation processes (i.e. reduction of intensity of the signals corresponding to the ions formed as a result of protonation of heteroatoms located in the heterocyclic ring). In the case of difluoroboranyl derivatives of 2-phenacylheterocycles, HCD leads to the formation of dominant [**X**-$\hbox {CH}_3^{\varvec{\cdot }}$+H]$^{+\varvec{\cdot }}$ products, related to the protonation of nitrogen of –N($\hbox {CH}_{{3}}$)$_{2}$. Additionally, the results confirm that the loss of $\hbox {HOBF}_{{2}}$, which has been observed in ESI MS/MS experiments performed for all difluoroboranyl derivatives (presented here and reported previously^28^), can serve as a signature tag for the presence of the –O-$\hbox {BF}_{{2}}$ moiety. As the elimination of neutral $\hbox {HSBF}_{{2}}$ was observed for both, the compounds containing –$\hbox {SBF}_{{2}}$^28^ group and those with –$\hbox {OBF}_{{2}}$ unit and sulfur atom located in the heterocyclic ring, this fragmentation process does not provide information about the location of the difluoroboranyl moiety and it does not play such analytical role as $\hbox {HOBF}_{{2}}$ elimination.

The $\hbox {NCH}_{{3}}$/S/O replacement in the five-membered ring has a strong impact on minor HCD fragmentation reactions. For example, in the case of compounds containing a sulfur atom, the loss of $\hbox {H}_{{2}}$S is observed for those with a heterocyclic oxygen atom, the elimination of CO occurs, and the presence of a N($\hbox {CH}_{{3}}$) group in the ring leads to detachment of the methyl radical.

The results clearly show that ESI HCD MS/MS provides detailed information on all structural motifs of the analyzed 2-phenacylheterocycles and their difluoroboranyl derivatives and can be used as a fast and simple tool for the structural analysis of novel difluoroborates. Furthermore, samples for MS and MS/MS analysis do not require any special, time-consuming preparation, a small amount (ppm) of compounds is required for the experiments, and HCD provides simple mass spectra that are easy to interpret. However, the elucidation of the details of the HCD fragmentation mechanisms of the analyzed compounds requires the involvement of theoretical chemistry methods, such as DFT, which provides valuable complementary information.

It should be underlined that the detailed theoretical study of the secondary fragmentation processes is not provided here. One need also note that the energetic, enthalpic and Gibbs free energy data should be interpreted with caution, taking into account the particular experimental HCD conditions which do not allow for reaching of the thermal equilibrium.

## Supplementary Information

Supplementary Information.
