# Supplementary material for: Tautomeric equilibrium, proton affinity and mass spectrometry fragmentation of flexible hydrogen-bonded precursors and rigid N ⟶ BF2 fluorescent dyes
Source: Sci Rep. 2021 Aug 6;11:15995. doi: 10.1038/s41598-021-94978-9 (PMC8346630; doi:10.1038/s41598-021-94978-9)
Supplement: Supplementary file 1 — Supplementary Information. [file 41598_2021_94978_MOESM1_ESM.pdf]

Supporting Information: Tautomeric Equilibrium, Proton Affinity and Mass Spectrometry Fragmentation of Flexible Hydrogen-Bonded Precursors and Rigid N⟶BF_2 Fluorescent Dyes[†]

Małgorzata A. Kaczorowska,[†] Anna Kaczmarek-Kędziera,[‡] and Borys Ośmiałowski[*,‡]

†*Faculty of Chemical Technology and Engineering, UTP University of Science and Technology, Seminaryjna 3, 85-326 Bydgoszcz, Poland*

‡*Faculty of Chemistry, Nicolaus Copernicus University in Toruń, Gagarina 7, 87-100 Toruń, Poland*

E-mail: borys.osmialowski@umk.pl

June 30, 2021

MS Measurements

The electrospray ionization of 1-methyl-2-phenacylbenzimidazole, 2-phenacylbenzothiazole and 2-phenacylbenzoxazole (compounds **1**-**3**), their dimethylamino derivatives (compounds **4**-**6**) and corresponding difluoroborates (compounds **7**-**9**), leads to the formation of the singly charged ions [**1**+H]$^+$ (m/z_{meas}=251.1184, m/z_{calc}=251.1184), [**2**+H]$^+$ (m/z_{meas}=254.0639, m/z_{calc}=254.0639), [**3**+H]$^+$ (m/z_{meas}=238.0867, m/z_{calc}=238.0868), [**4**+H]$^+$ (m/z_{meas}=294.1605, m/z_{calc}=294.1604), [**5**+H]$^+$ (m/z_{meas}=297.1062, m/z_{calc}=297.1061), [**6**+H]$^+$ (m/z_{meas}=281.1292, m/z_{calc}=281.1290), [**7**+H]$^+$ (m/z_{meas}=342.1593, m/z_{calc}=342.1589), [**8**+H]$^+$ (m/z_{meas}=345.1048, m/z_{calc}=345.1044) and [**9**+H]$^+$ (m/z_{meas}=329.1276, m/z_{calc}=329.1273), respectively. All the fragments detected in the HCD MS/MS experiments are described in Supplementary Tables S1-S9, which include exact and accurate masses and the calculated mass errors (ppm).

Fragmentation of 2-phenacylheterocycles

In addition to the main fragmentation observed in mass spectra and presented in the main text of the manuscript, the minor HCD fragmentation processes, detected for three analyzed ions, namely [**1**+H]$^+$–[**3**+H]$^+$, lead to the formation of products: $[C_5H_5]^+$, $[C_6H_5]^+$, $[C_6H_7O]^+$ ($[C_6H_5OH+H]^+$), [**X**-H_2O+H]$^+$ and [**X**-H_2O-H_2+H]$^+$ (where **X**= **1**, **2** and **3**). Generation of the $[C_6H_7O]^+$ species is most possibly related to the protonation of oxygen atoms from the hydroxyl groups of the precursors and requires, additionally, the transfer of the hydroxyl group to the benzene ring. Due to the nature of the HCD method, the most likely mechanism of formation of $[C_5H_5]^+$and $[C_6H_5]^+$ species is related to secondary fragmentation processes, such as the loss of CH_2O and elimination of H_2O from $[C_6H_7O]^+$ respectively.

The ESI HCD MS/MS spectrum of [**1**+H]$^+$ shows signals corresponding to ions containing both nitrogen atoms, such as $[C_8H_7N_2]^+$, $[C_8H_9N_2]^+$, $[C_9H_{10}N_2]^{+\cdot}$, $[C_9H_9N_2]^+$, $[C_9H_7N_2O]^+$ and $[C_{10}H_9N_2O]^+$. In case of most products it is possible to determine the structure of ions formed. For example, in the case of $[C_8H_7N_2]^+$ and $[C_8H_9N_2]^+$ ions, there is no doubt that

they contain the entire –N(CH_3) group in the heterocyclic ring ($[(C_6H_4)((NCH_3)(N))C]^+$ and $[(C_6H_4)((NCH_3)(NH))CH]^+$ respectively). The HCD mass spectrum of singly charged cations of 1-methyl-2-phenacylbenzimidazole shows also less abundant signals, which are related to the detachment of the methane molecule ($[\mathbf{1}\text{-}CH_4\text{+}H]^+$) and methyl radical from the nitrogen atom located in the heterocyclic ring ($[\mathbf{1}\text{-}H_2O\text{-}CH_3^{\bullet}\text{+}H]^{+\bullet}$). The elimination of CH_4 requires the migration of one proton within the molecule. The $[\mathbf{1}\text{-}H_2O\text{-}CH_3^{\bullet}\text{+}H]^{+\bullet}$ ions can be the result of the secondary fragmentation process, such as detachment of the methyl radical from $[\mathbf{1}\text{-}H_2O\text{+}H]^+$. Losses of the methyl radicals with low abundances have also been observed in the gas-phase dissociation of singly charged ions of another BODIPYs[S1] and of carbocyanine dyes containing methyl/ethyl groups attached to the nitrogen atoms from the heterocyclic ring.[S2] Minor fragmentation reactions in HCD of $[\mathbf{1}\text{+}H]^+$ cations lead to the generation of the ions $[C_{15}H_{12}N_2]^{+\bullet}$ and $[C_{15}H_{13}N_2]^+$ (i.e., $[\mathbf{1}\text{-}CH_2O\text{+}H]^+$). The simplest possible mechanism of the formation of $[C_{15}H_{12}N_2]^{+\bullet}$ species is related to the elimination of the hydrogen radical from the $[C_{15}H_{13}N_2]^+$ ions, formed as a result of the loss of the CH_2O molecule from the parent ions. Another possible dissociation pathway leading to the generation of the $[C_{15}H_{12}N_2]^{+\bullet}$ ions is associated with the transfer of the methyl group from the heterocyclic ring nitrogen atom to the hydroxyl group oxygen atom and, finally, rearrangement within the molecule. Intramolecular transfer of the methyl group upon the gas phase decomposition of protonated ions has been reported for various chemical compounds, including carbocyanine dyes.[S2,S3] Finally, the generation of the $[C_{15}H_{12}N_2]^{+\bullet}$ species may be associated with cleavages of two bonds between the carbon atom linked to a hydroxyl group and the adjacent carbon atoms (one double bond and one single bond), migration of two hydrogen atoms and intramolecular rearrangement. The migration of two protons in ESI MS/MS has been recognized previously, for example in experiments performed for singly charged ions of S-methyl benzenylmethylenehydrazine dithiocarboxylate and for other carboxamides.[S4,S5]

The HCD MS/MS spectrum of $[\mathbf{2}\text{+}H]^+$ ions shows similar signals, equivalent to that

observed for [**1**+H]$^+$, such as these corresponding to ions: [C_6H_5S]$^+$ ([C_6H_6N]$^+$ in the case of singly charged ions of compound **1**) and also signals which have not been observed in the gas-phase fragmentation of [**1**+H]$^+$, i.e. [**2**-H_2S+H]$^+$ and [**2**-HSOH+H]$^+$. The generation of [**2**-H_2S+H]$^+$ species is presumably related to the location of the ionizing proton at the sulfur atom and intramolecular hydrogen migration. It has been reported previously, that ionizing protons can also be located in less basic sites in molecules, (for example sulfur atoms), permitting charge-directed fragmentations.[S6–S8] The theoretical results also confirm such a possibility. The formation of the [**2**-HSOH+H]$^+$ ions requires the transfer of the –SH group or the sulfur atom (which is less likely, as the ionizing proton is located at the S atom) to the oxygen atom of the hydroxyl group (or vice versa, transfer of the OH group to a protonated sulfur atom), the cleavage of at least three bonds and intramolecular rearrangement. The transfer of unit/hydrogen in space is, possibly, related to the spatial arrangement of the fragments of the molecule. Intramolecular migration of protons through space, which depended on the spatial proximity of certain functional groups, has been also observed in electron capture dissociation (ECD) of the amyloid-β peptide[S9] and in fragmentation caused by collisions of carbocyanine dyes ions with inert gases.[S4]

Similarly to HCD of [**1**+H]$^+$ and [**2**+H]$^+$, the higher energy collisional dissociation of [**3**+H]$^+$ ions leads to the formation of products containing the entire heterocyclic ring (i.e., [C_8H_6NO]$^+$, [$C_9H_6NO_2$]$^+$). Both oxygen atoms present in the molecule can participate in the dissociation reactions. This is confirmed by the presence in the HCD MS/MS spectrum of the [**3**+H]$^+$ species of signals corresponding to [3-H_2O-CO+H]$^+$ ions, which most probably originate from secondary fragmentation processes. The involvement of an oxygen atom located in the heterocyclic ring in minor HCD fragmentation processes, which must be related to the protonation of this oxygen atom, is not unusual, given that a similar behavior was also noticed in the gas-phase dissociation of molecules containing an O atom in the furan moiety, i.e., N-(isoquinolin-1-yl)furan-2-carboxamide and metal ions/furan complexes.[S6,S10] The results of theoretical analysis also confirm the possibility of protonation of heterocyclic

oxygen atom.

For all of the examined 2-phenacylheterocycles in the ESI HCD MS/MS experiments, charge-directed fragmentation products are formed. The dominant dissociation reactions lead to the generation of similar ions and are related to the presence of heterocyclic ring nitrogen atoms (–N=) of all analyzed molecules which are favorable protonation sites. The properties of the heteroatoms located in the five-membered heterocyclic ring strongly affect minor fragmentation processes. Since all assignments of the dissociation products were made on the basis of the accurate mass measurement and verified by comparing the observed and the expected isotope patterns, there is no question as to the elemental composition or the charge of the ions formed.

Fragmentation of the dimethylamino derivatives of 2-phenacylheterocycles

A comparison of the fragmentation behavior of the ion pair $[\mathbf{1}+H]^+$ and $[\mathbf{4}+H]^+$, containing –N(CH_3) group in the heterocyclic unit allows for the conclusion that the presence of an additional –N$(CH_3)_2$ group attached to the benzene ring prevents the generation of $[C_8H_7N_2]^+$ and $[C_9H_{10}N_2]^{+\cdot}$ products which contain the entire five-membered ring. This phenomenon is possibly related to the differences in basicity of nitrogen atoms contained in molecules (e.g. –N=, –N(CH_3) and –N$(CH_3)_2$) that, in turn, controls the position of the attachment of ionizing protons. Both compounds **1** and **4** contain several nitrogen atoms of which the properties are not identical. The electron density at the nitrogen atom depends on many factors, such as: atom hybridization, the location of the electron pair, the nature and number of groups bonded to the atom.[S11] The presence of an additional electron-donating methyl group[S12] in N$(CH_3)_2$ increases the basicity of that nitrogen atom and, in effect, the protonation of the N atom from the dimethylamino group occurs. More information on the effect of a group –N$(CH_3)_2$ on electron distribution in molecules **4-6** can be found in the work by Grabarz *et al.*[S13]

The ESI HCD MS/MS spectrum of singly charged ions $[\mathbf{2}+H]^+$ containing a sulfur

Table S1: Fragments observed following higher energy collisional dissociation of singly charged ions of [**1**+H]+

m/z_{meas}	m/z_{calc}	Assignment	Mass Error (ppm)
251.1184	251.1184	$[\mathbf{1}+H]^+$ $(C_{16}H_{14}N_2O_1+H)^+$	0.00
235.0872	235.0871	$[\mathbf{1}-CH_4+H]^+$	0.42
233.1079	233.1079	$[\mathbf{1}-H_2O+H]^+$	0.00
231.0922	231.0922	$[\mathbf{1}-H_2O-H_2+H]^+$	0.00
221.1079	221.1079	$[C_{15}H_{13}N_2]^+$	0.00
220.1000	220.1000	$[C_{15}H_{12}N_2]^{+\bullet}$	0.00
218.0846	218.0844	$[\mathbf{1}-H_2O-CH_3^{\bullet}+H]^{+\bullet}$	0.91
173.0714	173.0715	$[C_{10}H_9N_2O]^+$	0.57
159.0557	159.0558	$[C_9H_7N_2O]^+$	0.63
145.0764	145.0766	$[C_9H_9N_2]^+$	1.37
146.0842	146.0844	$[C_9H_{10}N_2]^{+\bullet}$	1.37
133.0765	133.0766	$[C_8H_9N_2]^+$	0.75
131.0608	131.0609	$[C_8H_7N_2]^+$	0.76
105.0342	105.0340	$[C_7H_5O]^+$	1.90
95.0499	95.0497	$[C_6H_7O]^+$	2.10
92.0503	92.0500	$[C_6H_6N]^+$	3.26
77.0395	77.0391	$[C_6H_5]^+$	5.19
65.0396	65.0391	$[C_5H_5]^+$	7.67

atom in the five-membered moiety, compared with that obtained for their dimethylamino derivatives [**5**+H]$^+$, shows more signals corresponding to ions of which the formation is associated with the location of the ionizing proton at the S atom (i.e., [**2**-H_2S+H]$^+$, [**2**-HSOH+H]$^+$, $[C_6H_5S]^+$). Moreover, the higher energy collisional dissociation of ions of both 2-phenacylbenzothiazoles leads to the formation of $[C_9H_6NOS]^+$ species of which the generation is related to the location of the ionizing protons at the heterocyclic ring nitrogen

Table S2: Fragments observed following higher energy collisional dissociation of singly charged ions of [**2**+H]$^+$

m/z_{meas}	m/z_{calc}	Assignment	Mass Error (ppm)
254.0639	254.0639	[**2**+H]$^+$ ($C_{15}H_{11}SNO$+H)$^+$	0.00
236.0533	236.0534	[**2**-H_2O+H]$^+$	0.42
234.0376	234.0378	[**2**-H_2O-H_2+H]$^+$	0.85
220.0762	220.0762	[**2**-H_2S+H]$^+$	0.00
204.0813	204.0813	[**2**-HSOH+H]$^+$	0.00
202.0657	202.0657	[**2**-H_2O-H_2S+H]$^+$	0.00
176.0169	176.0170	[C_9H_6NOS]$^+$	0.57
148.0219	148.0221	[C_8H_6NS]$^+$	1.35
109.0112	109.0112	[C_6H_5S]$^+$	0.00
105.0341	105.0340	[C_7H_5O]$^+$	0.95
95.0499	95.0497	[C_6H_7O]$^+$	2.10
89.0394	89.0391	[C_7H_5]$^+$	3.37
77.0394	77.0391	[C_6H_5]$^+$	3.89
65.0395	65.0391	[C_5H_5]$^+$	6.15

atoms. However, the intensity of the signal corresponding to these ions is much lower in the HCD fragmentation of the dimethylamino derivative. Differences in the HCD fragmentation behavior of [**2**+H]$^+$ and [**5**+H]$^+$ ions can be rationalized on the basis of differences in the number and nature of atoms to which the ionizing proton(s) can be attached. Because the nitrogen atom of the dimethylamino group is a more favorable protonation site than the sulfur atom, the presence of the –$N(CH_3)_2$ group in [**5**+H]$^+$ ions causes, compared with [**2**+H]$^+$, reduction of HCD fragmentation products related to the location of a proton at the S atom. Competition between the heterocyclic and dimethylamino nitrogen atoms in the ionizing proton attachment leads to reduction of the amount of products of which the

Table S3: Fragments observed following higher energy collisional dissociation of singly charged ions of [**3**+H]$^+$

m/z_{meas}	m/z_{calc}	Assignment	Mass Error (ppm)
238.0867	238.0868	[**3**+H]$^+$ ($C_{15}H_{11}NO_2$+H)$^+$	0.42
220.0761	220.0762	[**3**-H_2O+H]$^+$	0.45
218.0653	218.0606	[**3**-H_2O-H_2+H]$^+$	21.55
210.0918	210.0919	[**3**-CO+H]$^+$	0.47
208.0762	208.0762	[**3**-CO-H_2+H]$^+$	0.00
192.0812	192.0813	[**3**-H_2O-CO+H]$^+$	0.52
160.0396	160.0398	[$C_9H_6NO_2$]$^+$	1.25
132.0448	132.0449	[C_8H_6NO]$^+$	0.76
105.0341	105.0340	[C_7H_5O]$^+$	0.95
95.0499	95.0497	[C_6H_7O]$^+$	2.10
89.0394	89.0391	[C_7H_5]$^+$	3.37
77.0394	77.0391	[C_6H_5]$^+$	3.89
65.0395	65.0391	[C_5H_5]$^+$	6.15

generation requires protonation of –N= atom from five-membered ring, but this only applies to minor fragmentation pathways.

The main difference in the HCD fragmentation behavior of singly charged ions of 2-phenacylbenzoxazoles with and without –$N(CH_3)_2$ group is the lower intensity of the signal corresponding to [$C_9H_6NO_2$]$^+$ ions and the lack of the signal which can be assigned to the [**6**-H_2O-CO+H]$^+$ species (adequate to [**3**-H_2O-CO+H]$^+$) for dimethylamino derivative. Moreover, the generation of a series of products, which due to the nature of HCD are most probably result of secondary dissociation processes of [**6**+H]$^+$, such as: [**6**-H_2O-H_2NCH_3+H]$^+$, [**6**-H_2O-CH_4+H]$^+$ and [**6**-H_2O-$CH_3^{\bullet}$+H]$^{+\bullet}$, confirms that the ionizing protons can be located on both, the oxygen atoms and the nitrogen atoms of a dimethylamino substituent.

Table S4: Fragments observed following higher energy collisional dissociation of singly charged ions of [**4**+H]$^+$

m/z_{meas}	m/z_{calc}	Assignment	Mass Error (ppm)
294.1605	294.1606	[**4**+H]$^+$ ($C_{18}H_{19}N_3O$+H)$^+$	0.34
276.1501	276.1501	[**4**-H_2O+H]$^+$	0.00
266.1294	266.1293	[**4**-C_2H_4+H]$^+$	0.37
261.1266	261.1266	[**4**-H_2O-$CH_3^{\bullet}$+H]$^{+\bullet}$	0.00
248.1188	248.1188	[**4**-H_2O-C_2H_4+H]$^+$	0.00
231.0921	231.0922	[**4**-H_2O-$HN(CH_3)_2$+H]$^+$	0.43
173.0713	173.0715	[$C_{10}H_9N_2O$]$^+$	1.15
148.0759	148.0762	[$C_9H_{10}NO$]$^+$	2.02
145.0765	145.0766	[$C_9H_9N_2$]$^+$	0.69
134.0604	134.0606	[C_8H_8NO]$^+$	1.49
120.0813	120.0813	[$C_8H_{10}N$]$^+$	0.00
103.0549	103.0548	[C_8H_7]$^+$	0.97
91.0549	91.0548	[C_7H_7]$^+$	1.09
79.0551	79.0548	[C_6H_7]$^+$	3.79
77.0386	77.0391	[C_6H_5]$^+$	6.49
67.0551	67.0548	[C_5H_7]$^+$	4.47

Fragmentation of the difluoroborates

The HCD MS/MS spectra of all protonated difluoroborates show abundant signals, which can be assigned to products formed as a result of elimination of the hydrogen fluoride molecule and lower-intensity signals corresponding to the detachment of neutral $HOBF_2$ species from parent ions ([**X**-HF+H]$^+$, ([**X**-$HOBF_2$+H]$^+$, where **X**=**7**, **8**, **9**). The neutral elimination of HF has been also observed as the dominant fragmentation process in the MS/MS experiments performed for various BODIPYs, in which a doubly-fluorinated

Table S5: Fragments observed following higher energy collisional dissociation of singly charged ions of [**5**+H]$^+$

m/z_{meas}	m/z_{calc}	Assignment	Mass Error (ppm)
297.1062	297.1061	[**5**+H]$^+$ ($C_{17}H_{16}N_2OS$+H)$^+$	0.34
282.0829	282.0827	[**5**-$CH_3^{\bullet}$+H]$^{+\bullet}$	0.70
279.0956	279.0956	[**5**-H_2O+H]$^+$	0.00
263.0643	263.0643	[**5**-H_2O-CH_4+H]$^+$	0.00
251.0642	251.0643	[**5**-H_2O-C_2H_4+H]$^+$	0.39
248.0535	248.0534	[**5**-H_2O-NH_2CH_3+H]$^+$	0.40
245.1080	245.1079	[**5**-H_2O-H_2S+H]$^+$	0.40
235.0455	235.0456	[$C_{15}H_9NS$]$^{+\bullet}$	0.42
234.0378	234.0378	[$C_{15}H_8NS$]$^+$	0.00
204.0813	204.0813	[$C_{15}H_{10}N$]$^+$	0.00
176.0168	176.0170	[C_9H_6NOS]$^+$	1.13
156.0730	156.0813	[$C_{11}H_{10}N$]$^+$	53.10
148.0759	148.0762	[$C_9H_{10}NO$]$^+$	2.02
134.0604	134.0606	[C_8H_8NO]$^+$	1.49
120.0813	120.0813	[$C_8H_{10}N$]$^+$	0.00
105.0579	105.0578	[C_7H_7N]$^+$	0.95
79.0551	79.0548	[C_6H_7]$^+$	3.79
67.0552	67.0548	[C_5H_7]$^+$	5.96

boron atom is coordinated by two nitrogen atoms[S1] and as minor dissociation process for difluoroboranyl derivatives of N-(pyridin-2-yl)-substituted benzoamides and N-(isoquinolin-1-yl)furan(thiophene)-2-carboxamides in which the BF_2 group is connected with an oxygen atom.[S6] The loss of $HOBF_2$ has been noted in the gas-phase dissociation of all analyzed difluoroborates (examined previously[S6] and presented here) containing a –OBF_2 moiety, while the elimination of $HSBF_2$ was observed for molecules in which the –BF_2 group is bonded

Table S6: Fragments observed following higher energy collisional dissociation of singly charged ions of [**6**+H]$^+$

m/z_{meas}	m/z_{calc}	Assignment	Mass Error (ppm)
281.1292	281.1290	[**6**+H]$^+$ ($C_{17}H_{16}N_2O_2$+H)$^+$	0.71
266.1054	266.1055	[**6**-$CH_3^{\bullet}$+H]$^{+\bullet}$	0.37
263.1183	263.1184	[**6**-H_2O+H]$^+$	0.38
248.0948	248.0949	[**6**-H_2O-$CH_3^{\bullet}$+H]$^{+\bullet}$	0.40
247.0872	247.0871	[**6**-H_2O-CH_4+H]$^+$	0.40
235.1234	235.1235	[**6**-H_2O-CO+H]$^+$	0.42
232.0760	232.0762	[**6**-H_2O-H_2NCH_3+H]$^+$	0.86
222.0917	222.0919	$[C_{15}H_{12}NO]^+$	0.90
220.0760	220.0762	$[C_{15}H_{10}NO]^+$	0.91
160.0396	160.0398	$[C_9H_6NO_2]^+$	1.25
148.0759	148.0762	$[C_9H_{10}NO]^+$	2.02
132.0448	132.0449	$[C_8H_6NO]^+$	0.76
122.0970	122.0970	$[C_8H_{12}N]^+$	0.00
120.0813	120.0813	$[C_8H_{10}N]^+$	0.00
110.0606	110.0606	$[C_6H_8NO]^+$	0.00
105.0580	105.0579	$[C_7H_7N]^+$	0.95
91.0550	91.0548	$[C_7H_7]^+$	2.19
79.0550	79.0548	$[C_6H_7]^+$	2.53
77.0348	77.0391	$[C_6H_5]^+$	55.81
67.0551	67.0548	$[C_5H_7]^+$	4.47
65.0395	65.0391	$[C_5H_5]^+$	6.15

to the sulfur atom.[S6] Interestingly, the HCD MS/MS spectrum of the [**8**+H]$^+$ ions reveals signals corresponding to [**8**-CH_3OBF_2+H]$^+$ and [**8**-CH_3SBF_2+H]$^+$ although the –BF_2 group in compound **8** is bonded to the oxygen atom and the sulfur atom is located in the het-

erocyclic five-membered ring. The mechanism of generation of [**8**-CH_3SBF_2+H]$^+$ must be different from that leading to the generation of [**8**-CH_3OBF_2+H]$^+$ products and is much more complicated. The formation of [**8**-CH_3SBF_2+H]$^+$ in a single step would require the transfer of the –BF_2 unit to the sulfur atom (possibly protonated), migration of hydrogen atoms, cleavage of several bonds and intramolecular rearrangement.

In addition to the general losses of $CH_3^{\bullet}$, HF and $HOBF_2$, the examined difluoroborates exhibit other similar, minor dissociation products, for example: [**X**-HF-CO+H]$^+$, ([**X**-HF-$CH_3^{\bullet}$+H]$^{+\bullet}$, [**X**-CH_3OBF_2+H]$^+$, [$C_{10}H_8N_2OBF_2$]$^+$ (adequate to [$C_9H_5NOSBF_2$]$^+$ and [$C_9H_5NO_2BF_2$]$^+$ in the case of singly charged ions of **8** and **9** respectively), [C_8H_8NO]$^+$, where **X**=**7**, **8**, **9**. The influence of the properties of heteroatoms located in the heterocyclic rings has been observed in the case of minor HCD fragmentation processes. For example, the generation of [**7**-$CH_3^{\bullet}$-$HOBF_2$+H]$^+$ has been noted only for the difluoroborate molecule containing –N(CH_3) group in a five-membered ring. This suggests that the methyl radical can originate from this group. Elimination of CH_3SH and CH_3SBF_2 molecules from singly charged ions of **8** ([**8**-CH_3SH+H]$^+$, [**8**-CH_3SBF_2+H]$^+$ is also noteworthy, since the formation of corresponding ions in the fragmentation of protonated compounds **7** and **9** is not observed. The generation of [**8**-CH_3SBF_2+H]$^+$ products requires intramolecular migration of group(s)/hydrogen atoms and rearrangement.

Dissociation of the analyzed ions strongly depends on all structural motifs, however, the properties of heteroatoms are of particular importance as a charge-directed fragmentation occurs. Furthermore, both the number and the properties of heteroatoms as well as their interactions are important. As a result of the replacement of a hydroxyl hydrogen atom with a BF_2 group, the dominant fragmentation process is the detachment of the methyl radical from the dimethylamino group, regardless of the composition of the heterocyclic ring. The properties of the nitrogen atoms occurring in 2-phenacylheterocycles are not identical, and this is reflected in the fragmentation processes observed. Dissociation reactions associated with the participation of the heterocyclic ring nitrogen atoms (–N=) are dominant, those

related to protonation of nitrogen atoms of the dimethyamino groups are not dominant but a large number of products is formed, those involving the N atoms of the –N(CH_3) groups of the heterocyclic rings are minor and a small amount of products is formed. The presence of a sulfur atom in the 2-phenacylheterocyles contributes to the occurrence of additional gas-phase fragmentation pathways (i.e., loss of H_2S) related to the location of an ionizing proton at the S atom. Interestingly, HCD creates also ions which result from the intramolecular transfer of the group/atom(s), for example [**2**-HSOH+H]$^+$, [**8**-CH_3SBF_2+H]$^+$. Such fragmentation behavior is presumably related not only to the properties of the sulfur atoms but also to the spatial proximity of certain functional groups.

Additionally, in ESI MS/MS experiments performed for 2-phenacylbenzoxazole (without –N(CH_3)$_2$ substituents) containing an oxygen atom in heterocyclic ring the combined loss of H_2O and CO has been observed, which confirms the involvement of both O atoms present in the molecule in fragmentation process, whereas the absence of a similar fragmentation channel in the gas-phase fragmentation of protonated dimethylamino and difluoroboranyl derivatives confirms that all structural motifs affect a collisional dissociation behavior.

Table S7: Fragments observed following higher energy collisional dissociation of singly charged ions of [**7**+H]$^+$

m/z_{meas}	m/z_{calc}	Assignment	Mass Error (ppm)
342.1593	342.1589	[**7**+H]$^+$ ($C_{18}H_{18}N_3OBF_2$+H)$^+$	1.17
327.1356	327.1354	[**7**-$CH_3^{\cdot}$+H]$^{+\cdot}$	0.61
322.1532	322.1527	[**7**-HF+H]$^+$	1.55
307.1293	307.1292	[**7**-HF-$CH_3^{\cdot}$+H]$^{+\cdot}$	0.32
294.1607	294.1578	[**7**-HF-CO+H]$^+$	9.85
276.1501	276.1501	[**7**-$HOBF_2$+H]$^+$	0.00
262.1346	262.1344	[**7**-CH_3OBF_2+H]$^+$	0.76
261.1266	261.1266	[**7**-$CH_3^{\cdot}$-$HOBF_2$+H]$^{+\cdot}$	0.00
246.1031	246.1031	[**7**-C_2H_6-$HOBF_2$+H]$^+$	0.00
221.0697	221.0697	[$C_{10}H_8N_2OBF_2$]$^+$	0.00
177.0662	177.0635	[$C_8H_7N_2OBF$]$^+$	15.24
148.0760	148.0762	[$C_9H_{10}NO$]$^+$	1.35
145.0765	145.0766	[$C_9H_9N_2$]$^+$	0.69
134.0604	134.0606	[C_8H_8NO]$^+$	1.49
120.0813	120.0813	[$C_8H_{10}N$]$^+$	0.00
91.0550	91.0548	[C_7H_7]$^+$	2.19
79.0551	79.0548	[C_6H_7]$^+$	3.79
65.0396	65.0391	[C_5H_5]$^+$	7.68

Table S8: Fragments observed following higher energy collisional dissociation of singly charged ions of [**8**+H]$^+$

m/z_{meas}	m/z_{calc}	Assignment	Mass Error (ppm)
345.1048	345.1044	[**8**+H]$^+$ ($C_{17}H_{15}N_2OSBF_2$+H)$^+$	1.16
330.0811	330.0810	[**8**-$CH_3^{\bullet}$+H]$^{+\bullet}$	0.30
329.0740	329.0731	[**8**-CH_4+H]$^+$	2.73
325.0987	325.0982	[**8**-HF+H]$^+$	1.54
319.0884	319.0888	[**8**-C_2H_2+H]$^+$	1.25
311.0856	311.0826	[**8**-CH_3F+H]$^+$	9.64
297.1065	297.1010	[**8**-CH_3SH+H]$^+$	18.51
279.0955	279.0956	[**8**-$HOBF_2$+H]$^+$	0.36
269.0748	269.0748	[**8**-C_2H_2-HBF_2+H]$^+$	0.00
265.0798	265.0799	[**8**-CH3OBF_2+H]$^+$	0.38
254.0641	254.0639	[$C_{15}H_{12}NOS$]$^+$	0.79
249.1029	249.1028	[**8**-CH_3SBF_2+H]$^+$	0.40
224.0152	224.0153	[$C_9H_5NOSBF_2$]$^+$	0.45
178.0325	178.0327	[C_9H_8NOS]$^+$	1.12
164.0167	164.0170	[C_8H_6NOS]$^+$	1.83
155.0715	155.0718	[$C_7H_8NBF_2$]$^+$	1.93
148.0760	148.0762	[$C_9H_{10}NO$]$^+$	1.35
134.0604	134.0606	[C_8H_8NO]$^+$	1.49
120.0813	120.0813	[$C_8H_{10}N$]$^+$	0.00
79.0551	79.0548	[C_6H_7]$^+$	3.79

Table S9: Fragments observed following higher energy collisional dissociation of singly charged ions of [**9**+H]$^+$

m/z_{meas}	m/z_{calc}	Assignment	Mass Error (ppm)
329.1276	329.1273	[**9**+H]$^+$ ($C_{17}H_{15}N_2O_2BF_2$+H)$^+$	0.91
314.1039	314.1038	[**9**-$\dot{C}H_3$+H]$^{+\cdot}$	0.32
309.1215	309.1210	[**9**-HF+H]$^+$	1.62
303.1112	303.1116	[**9**-C_2H_2+H]$^+$	1.32
281.1292	281.1261	[**9**-HF-CO+H]$^+$	11.03
263.1183	263.1184	[**9**-$HOBF_2$+H]$^+$	0.38
251.1185	251.1184	[**9**-CO-HBF_2+H]$^+$	0.39
249.1030	249.1028	[**9**-CH_3OBF_2+H]$^+$	0.80
247.0868	247.0871	[**9**-CH_3OBF_2-H_2+H]$^+$	1.21
237.1025	237.1028	[**9**-$C_2H_3OBF_2$+H]$^+$	1.26
208.0382	208.0381	[$C_9H_7NO_2BF_2$]$^+$	0.48
160.0396	160.0398	[$C_9H_6NO_2$]$^+$	1.25
148.0759	148.0762	[$C_9H_{10}NO$]$^+$	2.02
134.0604	134.0606	[C_8H_8NO]$^+$	1.49
120.0813	120.0813	[$C_8H_{10}N$]$^+$	0.00
91.0550	91.0548	[C_7H_7]$^+$	2.19
79.0550	79.0548	[C_6H_7]$^+$	2.53
67.0551	67.0548	[C_5H_7]$^+$	4.47

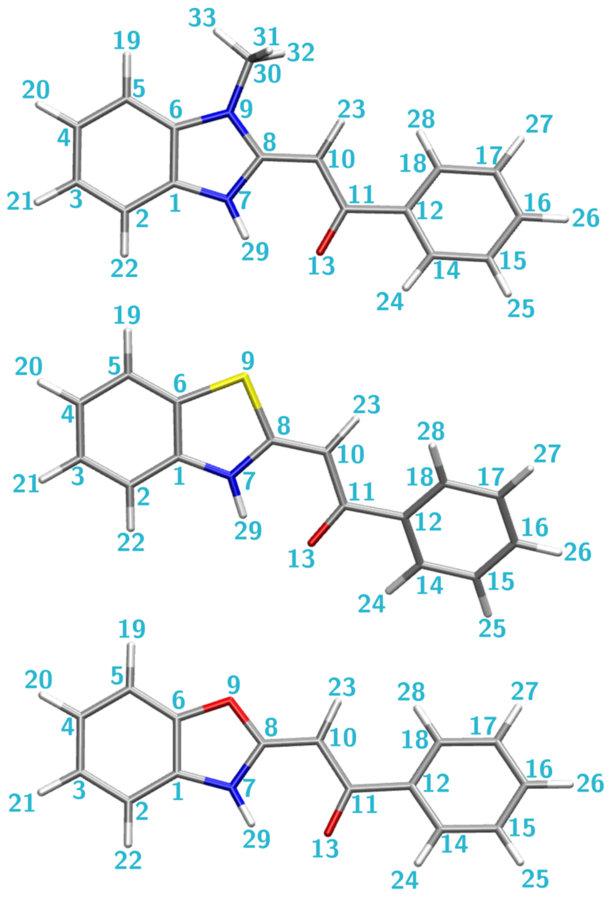

Figure S1: Atom numbering for neutral forms of **1**, **2** and **3**

Tautomeric equilibrium

Figs. S1–S3 contain the atom numebering for all of the investigated systems. In order to provide the reliable and thrustworthy theoretical data, the verification of the convergence of the relative energies with respect to the applied methodology has been performed for selected DFT functionals (among other double hybrid, B2PLYP-D3) as well as Hartree-Fock and MP2 approaches, representing the wave-function based formalisms. Additionally, the single point energy calculations have been carried out at the local correlation approach providing the gold standard of quantum mechanical calculations for large system, namely DLPNO-

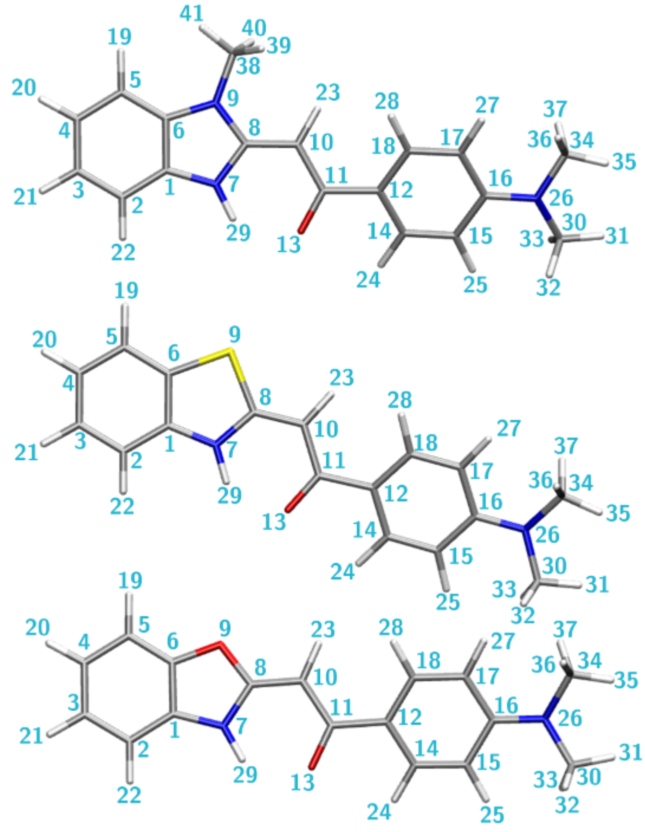

Figure S2: Atom numbering for neutral forms of **4**, **5** and **6**

CCSD(T)/aug-cc-pVTZ approach. The reliability of the coupled cluster approach has been confirmed by the T1 diagnostics, achieving the value smaller than 0.015 for all the investigated species. One can notice significant differences between DFT and WFT calculations, in particular for the MP2 approach (Fig. S4). The extraordinary stabilization of the keto form arises from the artificially strong bending of the optimal geometry, benefiting from the dispersion interactions between the aromatic parts of the molecule. The strongest effect is observed in the systems protonated at the terminal dimethylamine group, what corresponds to the strongest bending of the system (Fig. S5, panels (g)–(i)). Similar tendencies can be recognized for the relative Gibbs free energies of the tautomers (Fig. S5). These deviation is less pronounced for the MP2 single-point calculations for the ωB97X-D–optimized

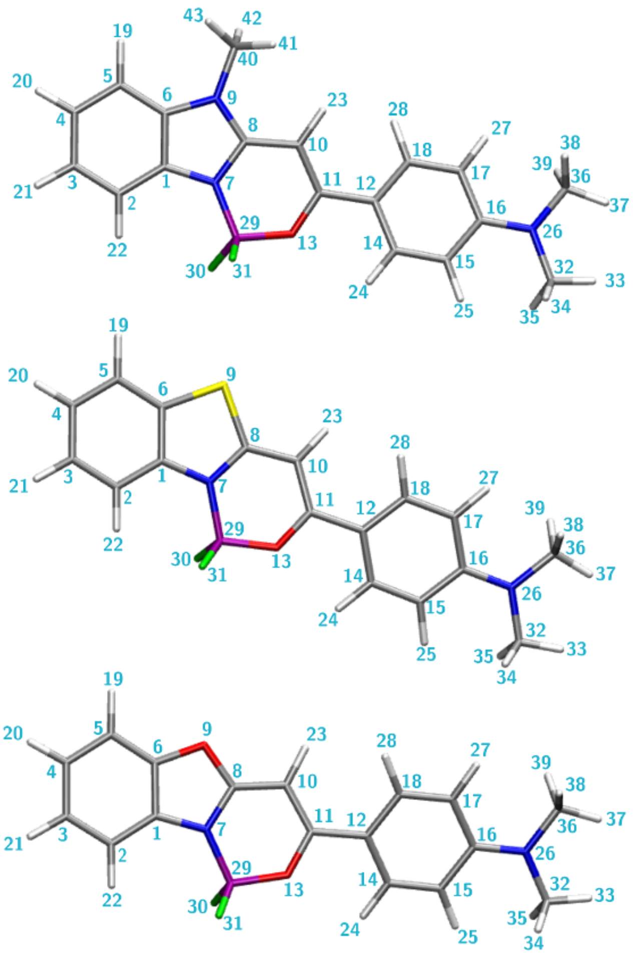

Figure S3: Atom numbering for neutral forms of **7**, **8** and **9**

geometry, as can be expected. Double hybrid functional confirms the reliability of the conventional range-separated ωB97X-D functional calculations, only slightly destabilizing the enamine for neutral species and stabilizing the enol tautomer for protonated systems. The qualitative agreement of the DFT calculations is verified for the reference DLPNO-CCSD(T) treatment, where all of the relative energies are smaller than 6 kcal/mol and the minor role of keto tautomer is noticed for the species protonated at the terminal dimethylamino group.

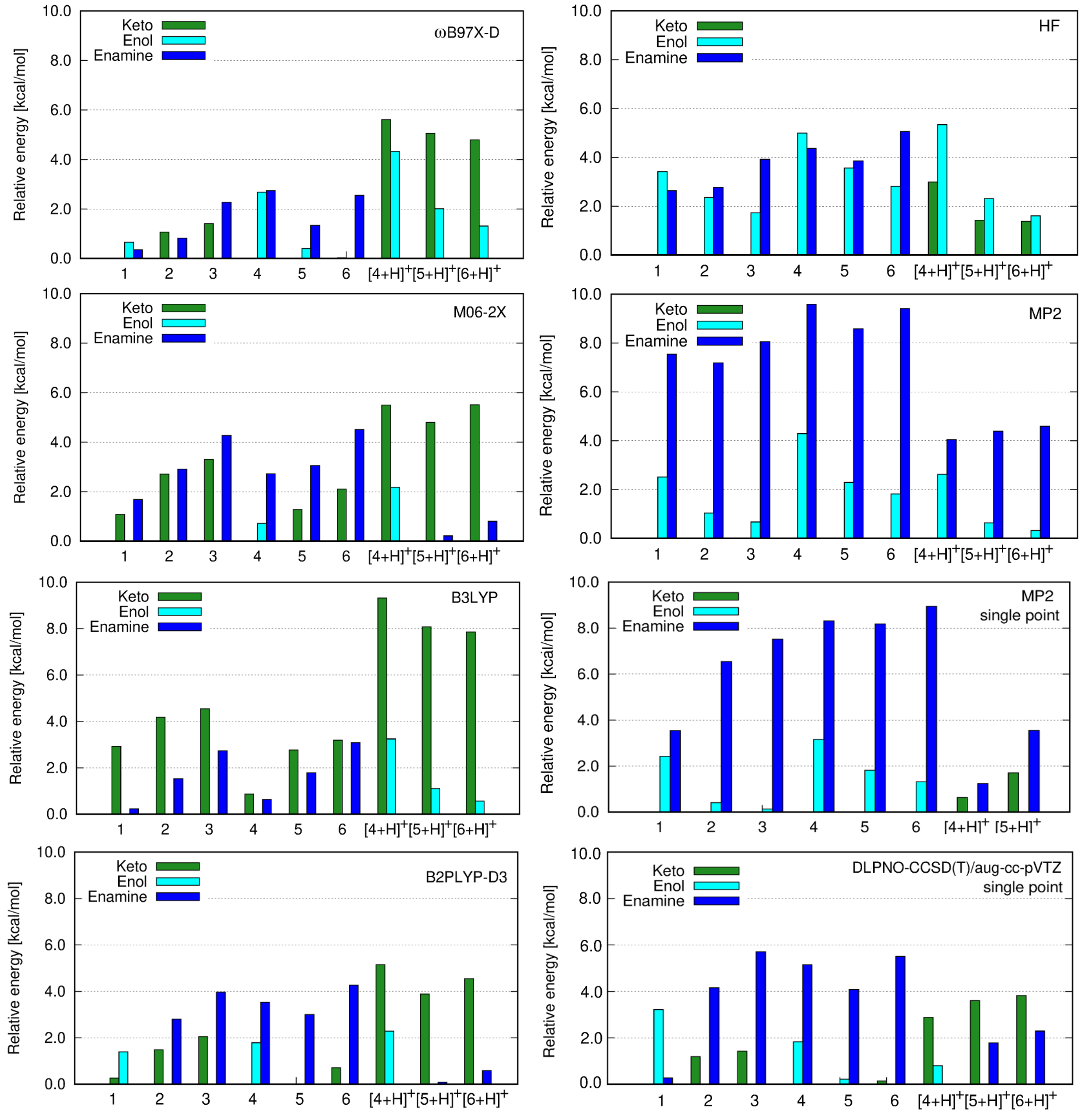

Figure S4: Relative energy for tautomers of **1**-**6** and the [**4+H**]$^+$–[**6+H**]$^+$ systems protonated at the terminal dimethylamine group: left panels within the DFT/6-311++G(d,p) approach and right panels in wave function theory approaches with 6-311++G(d,p) basis set except the DLPNO-CCSD(T) treatment (MP2 overestimation of the keto form stability with respect to the DFT results arises from the significantly bent structure, benefiting from the artificially overestimated dispersion attraction between the aromatic parts of the molecule)

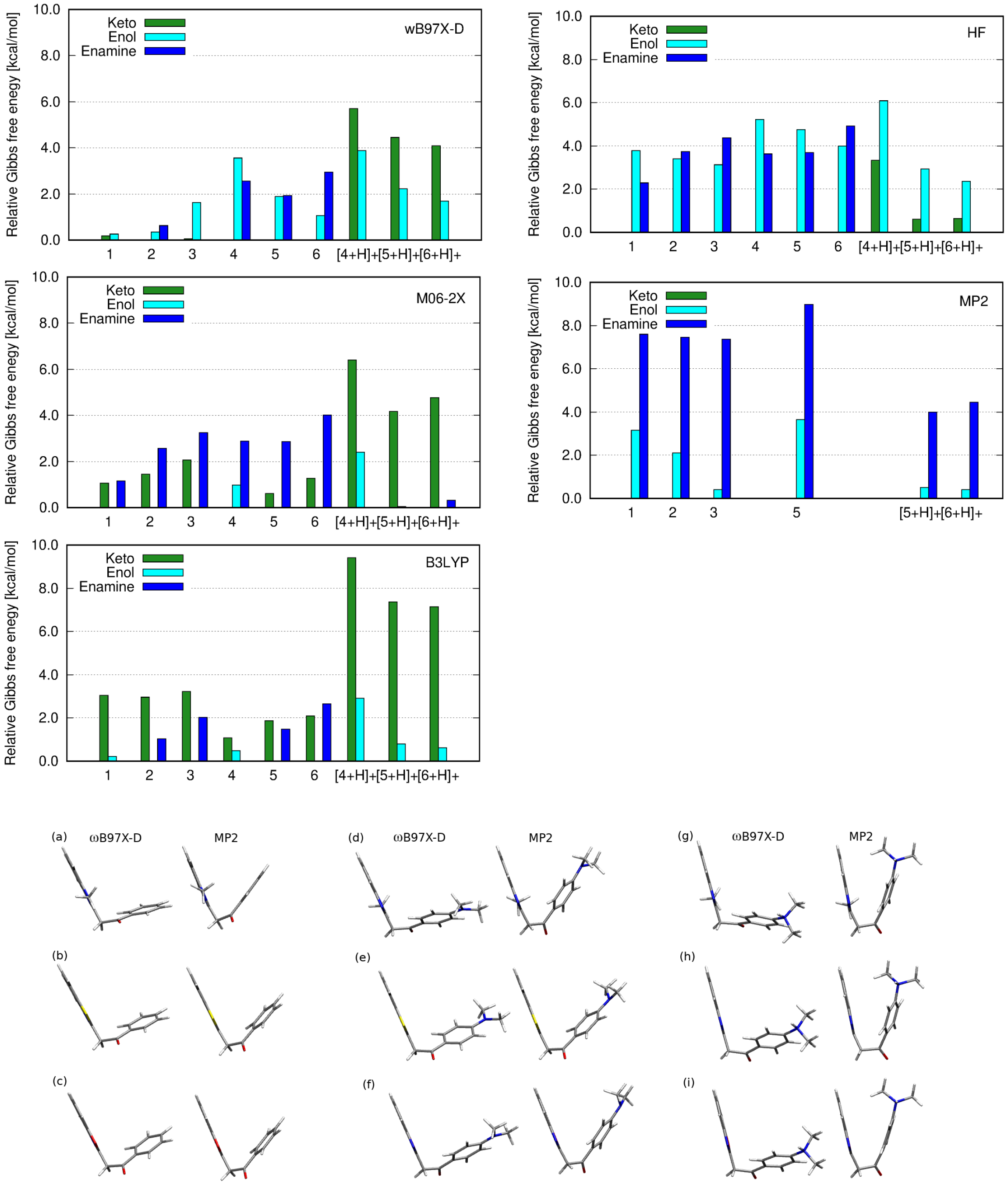

Figure S5: Relative Gibbs free energy for tautomers of **1**-**6** and the [**4+H**]$^+$–[**6+H**]$^+$ systems protonated at the terminal dimethylamine group. MP2 overestimation of the keto form stability with respect to the DFT results arises from the significantly bent structure, benefiting from the artificially overestimated dispersion attraction between the aromatic parts of the molecule, depicted schematically in lower part of the figure; (a) **1**, (b) **2**, (c) **3**, (d) **4**, (e) **5**, (f) **6**, (g) [**4**+H]$^+$, (h) [**5**+H]$^+$, (i) [**6**+H]$^+$

Proton affinity for 1–3

Table S10: Gas-phase basicity (GB) for investigated systems **1**–**6** [kcal/mol] estimated within the ωB97X-D/6-311++G(d,p) approach

Protonation site	**1**	**2**	**3**	**4**	**5**	**6**
			Keto (K)			
N7	229.23	218.10	214.85	236.57	225.34	229.56
N9/S9/O9	189.79	183.18	193.15	206.08	188.83	199.65
O13	198.88	200.03	198.13	215.57	217.15	217.98
N26				214.40	213.71	213.52
			Enol (O)			
N7	232.18	223.77	212.30	232.32	223.84	220.67
N9/S9/O9	194.58	186.45	168.97	202.74	188.49	177.65
C10	235.72	200.27	198.07	218.75	229.48	228.46
N26				219.61	217.83	216.98
			Enamine (E)			
N9/S9/O9	193.96	178.46	164.00	200.19	184.37	170.35
C10	200.33	225.14	224.03	243.60	227.27	232.95
O13	231.56	214.32	213.93	237.43	223.88	222.56
N26				238.50	220.09	220.56

For the neutral form of system **1**, twelve stable isomers have been found, differing in protonation pattern and rotations along the single bonds. The lowest relative energy is obtained for **1** bearing a native proton at C10 site (keto with carbonyl up: 0.00 kcal/mol and its rotamers, namely carbonyl perpendicular 2.30 kcal/mol and down 4.06 kcal/mol). Another stable form with a relative energy only 0.39 kcal/mol higher than the lowest one is a planar rotamer with a native proton at N7 atom (enamine). This isomer is stabilized additionally by the N-H…O interaction in six-membered ring and therefore can be consid-

ered favorable. Since the relative energy difference between those two isomers is below the accuracy level of performed calculations, one should be aware that the energetic ordering can be modified upon the change of the computational approach employed. The next stable species are found to possess the proton at N7 site (10.15 kcal/mol) and a following series of rotamers that contain proton at O13 atom (14.78 kcal/mol, 15.02 kcal/mol and 15.59 kcal/mol). Although this is not the full spectrum of possible conformers as it was obtained manually in the process of searching of possible proton transfer mechanisms in protonated species, one can expect that in the reaction mixture the prevailing forms are bearing a proton at C10 or N7 site, with a negligible amount of higher energy forms.

The first step in the ESI-HRMS measurement procedure is a protonation of the neutral form of the investigated species, governed by the basicity or proton affinity of the sites.[S14] Examined 2-phenacylheterocycles contain several heteroatoms that may be "attractive" sites for ionizing protons, namely nitrogen, oxygen or sulfur atoms. Their protonation affinity is presented in Fig. 3 of the main text of the manuscript, while the complementary gas-phase basicity is summarized in Tab. S10. The protonation of the lowest energy neutral form at N7 site is governed by the proton affinity equal to 236.56 kcal/mol (see Figure 3. of the main text), while for O13 site PA is 206.47 kcal/mol and the protonation at the heterocyclic N9 site is even less probable (PA equal to 197.79 kcal/mol). Therefore, it can be assumed that N7 protonation will be preferred. This leads to the bent protonated $[\mathbf{1}+H]^+$ ion 1.2 (see Figure 4. of the main text) with a relative energy of 4.59 kcal/mol with respect to other protonated forms of $[\mathbf{1}+H]^+$ found in the present study. Since the relatively low barrier (5.24 kcal/mol; however significant in comparison with kT in room temperature, that is equal to 0.59 kcal/mol) are required to be crossed in order to achieve the lowest energy planar $[\mathbf{1}+H]^+$ structure (protonated at N7 and C10 sites; relative energy 0.00 kcal/mol), the rotation along the C8-C10 bond appears as the attractive stabilizing mechanism of proton transfer in $[\mathbf{1}+H]^+$. Additionally, if one assumes that in the applied MS conditions the energy barriers can be easily crossed, also the easy proton transfer from

N7 to O13 site followed by the further molecular bending and hydroxyl group rotation could occur, producing the 1.4 isomer characterized by the relative energy of 33.14 kcal/mol. The position of the protonation sites in 1.4 allows to further transfer the mobile proton from C10 to O13 moiety, thus obtaining the high-energy 1.5 isomer doubly protonated at the hydroxyl group (relative energy of 72.98 kcal/mol). On the other hand, the close proximity of the C10 and O13 sites and the corresponding bending of 1.2 isomer can lead to the proton transfer to the 1.1 structure of the relative energy equal to 7.49 kcal/mol. However, for this reaction high barrier (50.64 kcal/mol) needs to be passed over and would be therefore less probable in the standard room temperature conditions than those previously discussed. Still the collisional energies in the applied HRMS methodology, of the order of 350-1050 kcal/mol, are sufficient to cross the highest barrier determined in the present study.

The detailed analysis of the Wiberg bond indexes for 1.3 provided in Table S11 can help to determine the weakest bonds in the molecules, for which the cleavage is most feasible. Two weakest bonds in 1.3 are the N9-C30 bond (Wiberg bond index 0.9258) what would correspond to the detachment of the methyl group from the heterocyclic ring and the C10-C11 bond (Wiberg bond index 0.9689) leading to the $[C_7H_5O]^+$ species observed as the strong signal in the mass spectra (Fig. 1, main text). In this case the remaining part of the molecule can create the $[C_9H_{10}N_2]^{+\bullet}$ radical cation, detected experimentally as well. Considering the high energies provided by the HCD MS/MS collisions, also the higher reaction barriers in the present case can be crossed. Therefore, starting from 1.4 ion the proton transfer from C10 to O13 can be achieved (system 1.5 in Figure 4. of the main text) by the reaction barrier of 70.91 kcal/mol, giving the species doubly protonated at oxygen site. This process significantly weakens the C11-O13 bond (Wiberg bond index 0.6890), thus leading to the viable H_2O detachment to $[1\text{-}H_2O+H]^+$ cation (experimental signal at 233 *m/z*). If one additionally notice the weak N9-C30 bond (Wiberg bond index 0.9420) in this system, the formation of $[1\text{-}H_2O\text{-}CH_3^{\bullet}+H]^{+\bullet}$ radical cation, recognized at 218 *m/z* in the experimental measurement, is rationalized. The proposed ESI HCD MS/MS fragmentation mechanism of

protonated compounds **1-3** is presented in Figure 5 of the main text.

The weak character of the C10-C11 bond in 1.2 protonated system is confirmed by the electron density calculated within AIM approach (see Supporting Information for the collected data). The smallest value of electron density ρ in the bond critical point, equal to 0.2350 atomic units (compare Tab. S12), consistent with the small Wiberg bond index, together with the relatively low energy of 1.2 (4.59 kcal/mol) support the fragmentation process at the C10-C11 bond, with the generation of the benzoyl ion. The 1.2 protonated system can be generated from the lowest energy form 1.3 by breaking of the weak intramolecular hydrogen bond of the energy estimated as 8.36 kcal/mol (Tab. S13) and crossing the small energy barrier (Figure 4 of the main text).

Twelve stable isomers of neutral form of **2** have been localized on the potential energy surface. The lowest energy planar enol form stabilized by the intramolecular N...H-O interaction is followed in the energetic sequence by the enamine planar tautomer exhibiting N-H...O contact and higher in energy by 0.82 kcal/mol. The keto tautomer in the bent form and carbonyl group pointing up (to the direction of sulfur part of heterocyclic ring) exhibits the relative energy of 1.06 kcal/mol. The rotation along the single C-C bond leads to the sequence of the three following keto tautomers with the carbonyl group either in the plane perpendicular to the heterocyclic ring (either pointing to the front or to the back of the molecule – two isoenergetic forms of 3.17 kcal/mol) or pointing down to the direction determined by the heterocyclic nitrogen, characterized by the relative energy of 2.44 kcal/mol. The remaining enol and enamine isomers lay higher than 10 kcal/mol and will not be present in a mixture in significant amount. The energetic order of the neutral **2** isomers and the corresponding proton affinities suggest that upon protonation the most abundant $[\mathbf{2}+\mathrm{H}]^+$ species arise from the enol planar form protonated at C10 site and indeed this seems to be the energetically favorable protonated form. The rotation along the C-C bond causes the opening of the hydrogen-bonded six-membered ring and produces the next energetically stable form bearing protons at N7 and C10 sites (relative energy of 6.54 kcal/mol). Since

this rotamer features the carbonyl oxygen approaching C10 protonated site, it allows for the proton transfer to O13 and formation of the stable enol form with another proton at N7. Further on it can again rotate easily to achieve finally the enol tautomer (N7 and O13 protonated) with these two protons in the deformed seven-membered cyclic arrangement, characterized by the relative energy of 9.97 kcal/mol. Although calculated proton affinity indicates the favorable protonation of **2** at N7, it has been proven that the protonation can also occur at less basic sites, namely at sulfur atom [22,28,29]. The PA(S9) value for enol form of **2** is equal to 189.74 kcal/mol and remains more than 40 kcal/mol lower than for the N7 protonation. This, on the other hand, could cause the weakening of the bonds in the heterocyclic ring (Wiberg bond order equal to 0.8540 for protonated S9 and C10 atoms to be compared to 1.3149 for the lowest energy isomer bearing protons at N7 and C10) and, as a consequence, its cleavage. These sulfur-protonated species lie higher in energy by more than 40 kcal/mol with respect to the most stable enol form, what would be of importance for the investigation in standard conditions, however in the mass spectrometry with high-energy collisions one can expect that this energetic cost is not prohibited. Due to the similar character of **1** and **2** protonated systems and their analogous energetics one can assume that all of the above-mentioned reaction barriers for thiazole will be of similar order of magnitude as in the case of imidazole and could be crossed easily in the experimental conditions of ESI HCD MS/MS. The comparison of the Wiberg bond indexes between most stable forms of protonated **1** and **2** exhibits that also for thiazole the weakest bond prone for fragmentation is the C10-C11 one with 0.9703 bond index which leads to the $[C_7H_5O]^+$ ion and the remaining $[C_8H_6NS]^+$ part. Next weakest bond is the C8-C10 one with the Wiberg bond index equal to 1.0470, however similarly as in the case of [**1**+H]$^+$ here also in MS there is no clear evidence of the fragmentation in this place. Another spot prone to the cleavage can be noticed at the C11-C12 bond (Wiberg bond index equal to 1.0762) with the formation of $[C_9H_6NOS]^+$ ion observed experimentally at 176 *m/z*. Assuming that crossing all of the reaction barriers for proton transfer in the investigated system is feasible upon the ESI

HCD MS/MS experimental conditions, the water loss is assigned to the fragmentation of the form doubly protonated at hydroxyl oxygen. Although its relative energy is equal to 61.32 kcal/mol and the reaction barrier can be expected to exceed 100 kcal/mol (by comparison to the corresponding values calculated for $[\mathbf{1}+H]^+$; see Figure 4. of the main text), the severe weakening of the C11-O13 bond with respect to other protonated forms (Wiberg bond order 0.6694) forces the formation of $[\mathbf{2}\text{-}H_2O+H]^+$ ion (compare Fig. 1. in main text). Likewise, as mentioned before, the isomer protonated at S9 atom exhibits the significant weakening of the heterocyclic ring bonds, and this effect is most severe for C8-S9 and N7-C8 bonds. Therefore, one can assume that in such a case, particularly in the enolic form, the heterocyclic ring is broken effortlessly (possibly in two subsequent steps), with fragmentation to the $[C_6H_5S]^+$ ion, detected in the experimental spectrum at 109 *m/z*.

Among the twelve stable isomers of the neutral form of **3**, the lowest relative energy is noticed for the enol planar form, stabilized with the intramolecular N…H-O contact, similarly as in the case of **2**. The strong stabilizing influence of hydrogen bond shall be noticed when juxtaposing this planar rotamer with any other enol form carrying the hydroxyl group rotated away from the heterocyclic ring. Such a destruction of the N…H-O interaction results with enol rotamers higher in energy by more than 12 kcal/mol. Comparable effects are observed in the case of the enamine tautomers, where the lowest energy one (2.28 kcal/mol) benefits from the presence of the intramolecular N-H…O contact and deformed rotamers, void of these advantageous interaction, appear more than 12 kcal/mol higher in energy. The third tautomeric form of **3**, keto, occurs as three different rotamers and the lowest among them is the isomer with the carbonyl group pointing up to the oxygen part of the heterocyclic ring (relative energy 1.48 kcal/mol). The rotation along the C-C single bond allows to obtain two remaining isomers, similarly as in the case of **2**, with the relative energy of 2.69 and 2.73 kcal/mol for down and perpendicular position of carbonyl group, respectively. Protonation of **3** in the multicomponent reaction mixture arising from the small energy differences between the tautomers of neutral oxazole is governed by the proton affinity values presented in Figure

3. of the main text. One can expect that the preferable protonation takes place for the lowest energy enol form at N7 site. Since the protonated isomer of the lowest relative energy bears protons at N7 and C10, the proton transfer is likely to occur in the initial [**3**+H]$^+$ structure, leading to the lowest energy protonated form. In the standard conditions one could expect that this reaction is not very credible due to the reaction barrier of more than 60 kcal/mol (estimated by the comparison to [**1**+H]$^+$ values, see Figure 4. of the main text) to be crossed for proton transfer and additional one for a proper C-C rotation in order to expose the corresponding protonation sites closely to each other. However the experimental collisions occurring during the ESI HCD MS/MS measurements grant the supply of the energy high enough to cross any barriers and therefore one can also expect that further proton transfer from C10 to O13 can appear, generating a doubly protonated –OH_2^+ group attached with a very weak C-O bond (Wiberg bond index equal to 0.6732). This, further on can lead to the fragmentation of the molecular ion with the detachment of H_2O molecule to the [**3**-H_2O+H]$^+$ moiety, observed experimentally at 220 *m/z*. The main fragmentation pattern for [**3**+H]$^+$, and therefore most intensive MS signals, can be expected – likewise – from the C10-C11 bond breaking (Wiberg bond index 0.9579) and $[C_7H_5O]^+$ generation. The remaining part, namely $[C_8H_6NO]^+$, appears in the experimental spectra at 132 *m/z*. The Wiberg bond indexes analysis for [**3**+H]$^+$ indicates another weak bond in this ion, which is prone to the fragmentation upon HCD collisions: C11-C12 bond (Wiberg bond index 1.0829). The resulting detachment of the phenyl ring leads to the $[C_9H_6NO_2]^+$ ion observed experimentally at 160 *m/z*. Moreover, the secondary fragmentation processes are expected to exploit the remaining weak bonds, namely C6-O9 (Wiberg bond index 0.8787), C1-N7 (1.0182) or C8-C10 (1.0754). One could also notice that the O9-protonation of the keto form could lead either to the easy O9-O13 proton transfer, if geometrically feasible, or in the case of O13 carbonyl group pointing down, to the heterocyclic ring opening with O9-C8 bond breaking.

Proton affinity for 4–6

Twelve stable isomers of neutral form of **4** have been determined within the ωB97X-D/6-311++G(d,p) approach. Likewise, in the case of **1**, the lowest energy tautomer belongs to the keto group with the carbonyl pointing up in the same direction as methyl group in heterocyclic ring. The remaining keto rotamers are higher in energy by 2.49 and 4.95 kcal/mol for downwards and perpendicular position of carbonyl group, respectively. Next there are two following planar structures stabilized by the intramolecular hydrogen bond, namely enol form of the 2.68 kcal/mol relative energy and enamine (2.74 kcal/mol). It should be noticed that the relative energies of the rest of the enol and enamine isomers fall below 20 kcal/mol. Proton affinities estimated for these neutral **4** isomers indicate clearly the strong preference for the protonation at the N7 site in all tautomers, while protonation at C10 appear to be least attractive (compare PAs given in Figure 3. of the main text). Therefore one can expect that for the lowest energy neutral keto form of **4**, protonation occuring at N7 site together with a C-C single bond rotation leads to the most stable protonated $[\mathbf{4}+H]^+$ planar isomer 4.3 (Figure 6. of the main text). After the easy C-C rotation to 4.4 (relative energy 2.92 kcal/mol), the following intramolecular proton transfer requiring the crossing of the 47.95 kcal/mol energy barrier contribute to the creation of the N7/O13 protonated isomers 4.5 of the relative energy equal to 8.86 kcal/mol. On the other hand, nearly barrierless proton transfer between N7 and O13 in the low energy planar rotamer can occur, thus opening further possibilities of generation of the 4.1 form (Figure 6. of the main text). Although here higher energy gain is mandatory to cross the 100 kcal/mol barrier, all of these are available in present experimental setup. The substitution of **4** with the terminal dimethylamino group opens additional possibilities of proton transfer with respect to system **1**. Namely, one proton can be fixed at dimethylamino moiety, while the other one can be freely transferred between different protonation sites in the central part of the molecule (one of which is native and the second one coming from ESI procedure). This whole mechanism takes place at the energies of about 20 kcal/mol higher than the most stable protonated

form 4.3 and exhibits barriers up to about 90 kcal/mol. Again – although large in the room temperature, in the ESI-HRMS conditions all of these processes can easily occur (Figure 6. of the main text).

For system **4**, similarly as in the case of **1**, the strongest signal in mass spectrum arises from the fragmentation at the C10-C11 bond (Wiberg bond index 0.9601 for 4.3 isomer) to $[C_9H_{10}NO]^+$ ions. 4.1 isomer can be expected to lose easily the water molecule, giving [**4**-H_2O+H]$^+$ signal in HCD MS/MS. Moreover, among the weakest bonds in [**4**+H]$^+$ one need to take into account N9-C30 bond (Wiberg bond index 0.9270 in 4.3, 0.9422 in 4.1), which in a secondary process following the water molecule detachment from 4.1 protonated isomer can produce [**4**-H_2O-$CH_3^{\bullet}$+H]$^{+\bullet}$, observed experimentally at 261 *m/z*. Still here one could also consider the detachment of the $CH_3^{\bullet}$ moiety from the terminal dimethylamino group, since the corresponding Wiberg bond order for N26-C30 or N26-C34 is even lower than N9-C30 (namely 0.8934 and 0.8943). Again the terminal dimethylamino group disconnection due to the low Wiberg bond index (0.8910) in a protonated 4.7 isomer (see Figure 6. of the main text) can be assumed to be responsible for the experimental signal at 230 *m/z* ascribed to [**4**-H_2O-$HN(CH_3)_2$+H]$^+$.

Among twelve isomers of neutral system **5**, the most stable one is the keto form with the bent molecular skeleton. Rotation along the C-C single bond leads to the perpendicular keto form of the relative energy equal to 2.28 kcal/mol. Additional tautomers featuring the significant contribution to the reaction mixture are – similarly as in the case of **1** – planar enol and planar enamine forms, characterized by the relative energy of 0.40 and 1.34 kcal/mol, respectively. The rotation of the enol and enamine tautomers leads to the species of the relative energy lower than 15 kcal/mol of the minor significance for the composition of the mixture. For the most stable neutral forms protonation is expected to occur again at N7 site (PA higher than 230 kcal/mol), however one can expect that also the remaining protonation sites can be exploited, including a sulfur atom, despite its low proton affinity (of the order of 190 kcal/mol), alike for **2**. Protonation produces numerous isomers, of which

the lowest relative energy characterizes the form bearing protons at N7 and C10 sites. The vital modification of the electronic distribution and thus the molecule characteristic features follows from the protonation at the terminal dimethylamino group. This phenomenon alters the tautomeric equilibrium of **5** in favour of the enamine form, in contrast to imidazole case, when upon N26 protonation the enolic form appears to be the most stable. According to the predictions based on the proton affinity, also the energetics of the protonated forms of **5** confirms that the protonation at S9 site is unfavorable (the relative energy higher than 50 kcal/mol). However, all of these energy barriers can be crossed in ESI HCD MS/MS measurements and the S9 protonation, likewise for **2**, weakens the bonds in heterocyclic rings and allow for its destruction. Nevertheless, in contrary to **2**, the possible fragments arising from the C8-S9 and C1-N7 bonds cleavage are not observed experimentally as a products of primary fragmentation process and the differences between **2** and **5** in this aspect could be elucidated on the basis of the number and appeal of the accessible protonation sites in both molecules (compare discussion of experimental data on page 9 of main text). From the computational point of view, the difference between the proton affinity at N7 and S9 sites is about 20% larger in the case of **5** than for **2** (see Figure 3. of the main text), what acts to the benefit of nitrogen protonation thus downplaying the sulfur protonation processes and its consequences. Beside the sulfur protonation, **2** and **5** exhibit numerous similarities in the fragmentation pattern. The most intensive HCD MS/MS signal arises from the $[C_9H_{10}NO]^+$ ion, created by the C10-C11 bond cleavage (Wiberg bond index 0.9609). This is the most vulnerable point of **5**, except of two N26-C bonds in terminal dimethylamino group (Wiberg bond indexes 0.9455 and 0.9499). These bonds' breaking can be responsible for the detachment of $CH_3^{\bullet}$ moiety and thus for less intensive signals in the experimental measurements. Additionally, as in the earlier systems, protonation at the hydroxyl group, although less beneficial energetically and less attractive with respect to the proton affinity of oxygen site, occurs to attenuate the C11-O13 bond (Wiberg bond order 0.6095) so much that [**5**-H_2O+H]$^+$ signal appears in ESI HCD MS/MS at 279 *m/z* being the second most

intensive in the whole spectrum. Another slightly stronger C11-C12 bond (Wiberg bond index 1.1538) cleaves producing the $[C_9H_6NOS]^+$ ion, also registered experimentally, but with much lower signal intensity.

Ten optimized stable isomers of neutral forms of **6** provides again the five species which are to be present in the reaction mixture in significant amounts. The lowest relative energy is obtained for the planar enol form stabilized by the N...H-O intramolecular interaction. A virtually isoenergetic is the keto form of the bent skeleton with the carbonyl group pointing up (to the same direction as the oxazole's oxygen). The C-C bond rotation of the keto form leads to the two other rotamers of the relative energy equal to 1.40 and 1.60 kcal/mol respectively for the perpendicular and downward carbonyl. The fifth abundant isomer is the planar enamine form of the relative energy of 2.55 kcal/mol, exhibiting the intramolecular hydrogen bond as well. Enol and enamine rotamers provide the zoo of isomers of the relative energies below 15 kcal/mol. Among the protonated forms, the most stable one occurs to be the planar enamine protonated at C10 site, followed by the enol tautomer protonated in the same C10 site (relative energy 2.24 kcal/mol), both stabilized by the intramolecular hydrogen bond. The relative energies below 10 kcal/mol are also estimated for the O13-protonated enamine (5.98 kcal/mol) and its rotamer mildly twisted from planarity (8.15 kcal/mol) and the enamine protonated at N26 (9.75 kcal/mol). Wiberg bond indexes in $[\mathbf{6}\text{+H}]^+$ indicate that one of the weakest points in the cation is the C10-C11 bond (Wiberg bond index 0.9584), which can cleave easily producing two fragments, namely $[C_9H_{10}NO]^+$ and $[C_8H_6NO]^+$, which exhibit respectively the strongest experimental signal at 148 *m/z* and small intensity signal at 132 *m/z*. Another spot prone to fragmentation is determined as N-C bonds in the terminal dimethylamino group. Thus, the detachment of the $CH_3^{\bullet}$ radical (or alternatively its intramolecular transfer) can occur leading to the weak signal at 266 *m/z*. The protonation of **6** at the oxygen of the hydroxyl group in enol tautomer decreases the C11-O13 bond order (Wiberg bond index 0.6252), therefore justifying the disconnection of H_2O fragment (experimental evidence at 263 *m/z*). The main difference in the HCD

MS/MS of **1-3** and their dimethylaminated derivatives **4-6** lie in the intensity of the signal arising from fragments generated by the C11-C12 bond cleavage. The detailed comparison of the Wiberg bond indexes (1.0679, 1.0762, 1.0829 for **1-3** respectively and 1.1370, 1.1538 and 1.1557 for **4-6** in their most stable form bearing protons at N7 and C10) can provide a hint: the terminal substitution with a dimethylamino group strengthens the bond of interest in this manner decreasing the probability of the C11-C12 bond cleavage. The protons at C10 and O13 increase the Wiberg bond index in **1** and **4** respectively to 1.2109 and 1.3691, confirming the higher endurance of the C11-C12 bond in dimethylamino substituted systems independently on the protonation pattern.

Proton affinity for 7–9

For system **7**, the most intensive experimental signal at 327 *m/z* appears from the molecular ion with the removed methyl radical. This fragment can arise either from the terminal dimethylamino group or the methyl substituent at the heterocyclic ring. Protonation of **7** at N26 site diminishes the strength of the both N26-C32 and N26-C36 bonds thus making more probable the fragmentation with a $CH_3^{\bullet}$ radical arising from terminal dimethylamino group. However, the comparison of the Wiberg bond indexes for N26-C32/C36 and N9-C40 bonds (compare Table S18) does not give unambiguous answers, since all the analyzed bond orders are below 1.0000 and N9-C40 becomes stronger only in the case of O13 protonation (Wiberg bond index 1.2591). Therefore, only the further comparison between the computational and experimental fragmentation patterns in **7**, **8** and **9** and experimentally observed [**X**-$CH_3^{\bullet}$+H]$^{+\bullet}$ radical ions in all of these three cases allows to deduce that $CH_3^{\bullet}$ radical appears rather from the terminal dimethylamino group.

The next intensive signal in the ESI-MS measurement (148 *m/z*) can be generated by the simultaneous or subsequent breaking of two bonds: C10-C11 characterized by the Wiberg bond index 0.9993 and O13-B29 one (0.5574). The third most abundant ion giving the strong

experimental signal at 322 *m/z* emerge from the ESI proton attacking the well-exposed part of an ion, namely fluorine sites. Although protonation affinity at F is significantly lower than for the C10 site (217.21 kcal/mol versus 234.32 kcal/mol), the spatial arrangement of the whole system suggests that it could be easier to protonate F than any other more crowded sites with steric hindrances. The weak B-F bonds (0.7339 and 0.7274 Wiberg bond indexes before protonation) induce the easy HF removal from the [**7**+H]$^+$ ion upon the proton attack at F sites and result in a mild ring strenghtening after the HF detachment. Starting from this [**7**-HF+H]$^+$ cation, the C8-C10 bond (Wiberg bond index 1.2640) together with the C11-O13 bond (Wiberg bond index 1.0076) breaking produces the $[C_8H_7N_2OBF]^+$ cation arising at 177 *m/z* with a low intensity. The remaining experimentally observed species appear also in small intensities of the ESI-HCD MS/MS signals. The O13 protonation additionally weakens the strength of the O13-B29 bond to 0.3830 leading to the detachment of the $HOBF_2$ unit, which gives the low-intensity experimental signal at 276 *m/z*. When this detachment occurs concurrently with the N-C bond breaking in the heterocyclic ring, the [**7**-$CH_3^{\bullet}$-$HOBF_2$+H]$^{+\bullet}$ cation radical is observed at 261 *m/z*. Here again neither the N-C bond orders (0.93 vs 0.94) nor the energetics of the protonated forms allow to doubtlessly confirm the origin of this methyl radical, however since such a fragment is not observed neither for **8** nor for **9**, one can assume that this particular $CH_3^{\bullet}$ radical arises from the heterocyclic ring. Furthermore, the C11-C12 bond featuring the Wiberg bond index of 1.0261 after the N26 protonation is prone to breaking with the detachment of the $[C_8H_{10}N]^+$ ion observed at 120 *m/z*.

The lowest energy [**8**+H]$^+$ ion is protonated at C10 site (8.1) and can undergo the proton transfer process to O13 site crossing the barrier of 68.62 kcal/mol and arising at the O13-protonated moiety (8.2) of the relative energy equal to 22.72 kcal/mol. Next, the proton can be shifted to N7 site (8.3) of only slightly higher relative energy (23.00 kcal/mol) via the reaction barrier of 35.92 kcal/mol. The high following energy barrier of 59.31 kcal/mol for proton transfer to S9 site allows to achieve high-energy tautomer (relative energy of 47.65 kcal/mol), which however can be reached in the ESI HCD MS/MS experimental conditions

despite the comparably low proton affinity of sulfur in this system. Additionally, ESI proton attacking the N26 site can generate the isomer protonated at the terminal dimethylamino group of the energy equal to 6.03 kcal/mol, which would be also abundant in the reaction mixture in standard conditions. The experimental ESI HCD MS/MS spectrum shows the presence of the two large intensity signals despite the molecular [**8**+H]$^+$ ion: one arises from the detachment of the methyl radical (Wiberg bond order of N26-C32 and N26-C36 bonds equal to 0.8938 and 0.8933, respectively in a N26 protonated form) and the other one at 148 *m/z*, ascribed to $[C_9H_{10}NO]^+$, can be generated by the subsequent or simultaneous C10-C11 and O13-B29 bond breaking which can occur for instance for C10-protonated isomer characterized by the Wiberg bond indexes 0.9981 and 0.5807, respectively for the two mentioned bonds. Similarly, as in the case of **7**, also here the protonation at F site can be noticed regardless of the relatively small protonation affinity, leading to the HF detachment. However, the corresponding HCD MS/MS signal is significantly less intensive than in the case of **7**. One could assume that the origin of these differences lies in slightly larger proton affinity at fluorine site for **7** than for **8** and **9** (compare 217.21 kcal/mol for **7** with 211.29 and 210.21 kcal/mol for **8** and **9**, respectively). However comparison of the relative energies of the [**X**+H]$^+$ isomers (for **X**=**7**, **8** and **9**) protonated at F (18.39 kcal/mol, 19.05 kcal/mol and 18.23 kcal/mol respectively) does not confirm this reasoning definitely. Moreover, in [**8**+H]$^+$ the weak construction of the BF_2-containing ring becomes even weaker in comparison to [**7**+H]$^+$, with N7-B29 Wiberg bond index smaller than 0.6 for almost all protonated forms except 8.2 one, protonated at O13. This could facilitate the BF_2-ring breaking and the rotation of the BF_2 moiety to the S9 site of the heterocyclic ring. Several stable structures with a S9-B29 bond has been found, however all of them are characterized by the relative energies of the order of 50 kcal/mol or higher. Nevertheless, even such a large energy cost (including significantly higher energy barriers for this rotation) can be overcome in the experimental ESI HCD MS/MS conditions. Therefore, one could expect that the [**8**-CH_3SBF_2+H]$^+$ signal observed at 249 *m/z* arise indeed from the two-step process including the rotation of the

O-BF_2 moiety to S9 site and concurrent O13-B29 bond breaking and methyl group transfer. Another small intensity signal occurring at 120 *m/z* is ascribed to the $[C_8H_{10}N]^+$ cation is formed as a consequence of the C11-C12 bond breaking (Wiberg bond index dimished to 0.8812 upon the O13 protonation).

The protonation affinity order for **9** directly follows the sequence observed both for **7** and **8**. Therefore, one can expect the preferable protonation the C10 site, with production of the 9.1 protonated system of the lowest relative energy (see Figure S7). The intramolecular proton transfer from C10 to O13 site can be governed by the 68.21 kcal/mol delivered to the system for the barrier crossing. As the result, the 9.2 structure of the relative energy equal to 21.65 kcal/mol is obtained. Further on, the proton can be transferred to the N7 site, via the 35.77 kcal/mol barrier, and this reaction is only slightly endoenergetic, producing the 9.3 isomer of the relative energy 23.02 kcal/mol. The higher strains are necessary to allow for the consecutive proton transfer to the N9 site in the heterocyclic ring. Therefore, the calculated energy barrier is 80.85 kcal/mol and the N9-protonated isomer features the relative energy of 58.96 kcal/mol. On the other hand, if the ESI proton attacks directly the dimethylamino group, the obtained 9.5 structure is only slightly less stable than the C10-protonated most stable one (relative energy equal to 4.63 kcal/mol), thus it would be the abundant cation in the reaction mixture in standard conditions, similarly as in the other difluoroborate cases considered here. The analysis of the Wiberg bond indexes again allows to verify the probability of the various fragmentation channels. The most intensive signal observed experimentally arises from the methyl radical detachment from the [**9**+H]$^+$ ion, where the smallest Wiberg bond indexes are noticed in the case of the N26 protonation (0.8932 and 0.8936 respectively for N26-C36 and N26-C32 bonds). Bypassing the initial molecular ion [**9**+H]$^+$ signal, the next high intensity signal occurs at 148 *m/z* and emerge from the $[C_9H_{10}NO]^+$ cation, similarly as in the case of **8**, appearing by the C10-C11 bond breaking (Wiberg bond index in C10-protonated 9.1 system equal to 0.9981). Likewise, in **8**, the HF detachment is also registered at 309 *m/z*, despite the protonation affinity at F site

considerably lower than for C10 or N26 sites, leading to the molecular complex of the relative energy equal to 18.23 kcal/mol. The molecular ion denoted in Fig. 3 of the main text as [**9**-$HOBF_2$+H]$^+$ can arise from one of the two stable structures protonated at the O13 site: the 9.2 isomer with the relative energy of 21.65 kcal/mol or less probably due to the much higher relative energy (59.22 kcal/mol) and corresponding large reaction barriers, the one biding the BF_2 moiety between two oxygen atoms (namely O9 and O13) after the intramolecular rearrangement with a rotation similar as in the case of thiazole **8** system. The comparison of the Wiberg bond indexes in these two structures (O13-B29 0.3737 and B29-N7 0.6442 for 9.2 and respectively O13-B29 0.4882 and B29-O9 0.4656 for the rotated one) allows to assume that the detachment of the $HOBF_2$ fragment from the lower energy structure should be more feasible in standard conditions, however in the extreme ESI HCD MS/MS energy range, both mechanisms are plausible. The last low intensity signal appearing at 120 *m/z* and easy for the theoretical explanation, alike in **8**, arises from the C11-C12 bond breaking characterized by the Wiberg bond index equal to 1.0260 for the N26-protonated isomer.

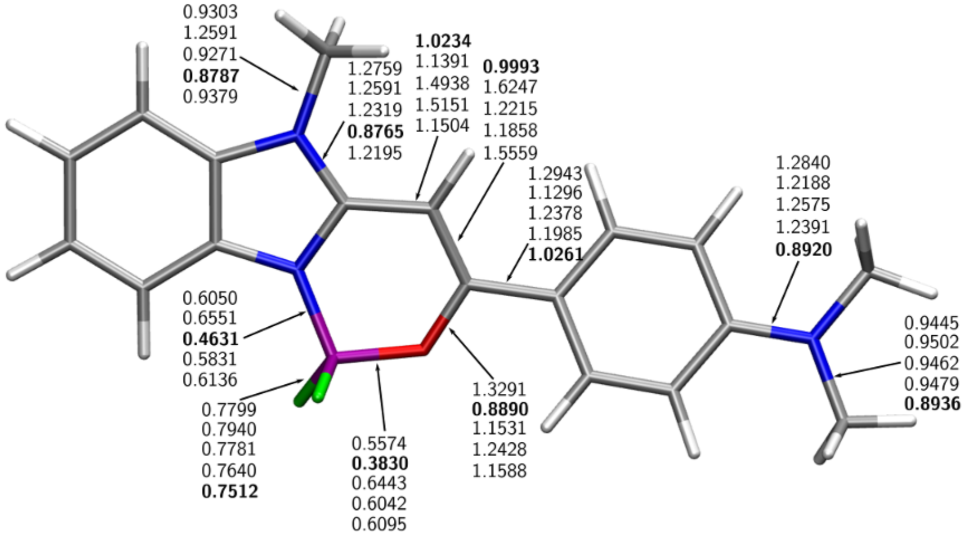

Figure S6: Wiberg bond indexes for protonated forms of **7** in the following order from top to bottom: 7.1 (protonated at C10; relative energy 0.00 kcal/mol), 7.2 (protonated at O13, relative energy 21.58 kcal/mol), 7.3 (protonated at N7; 23.28 kcal/mol), 7.4 (protonated at N9; 35.54 kcal/mol) and 7.5 (protonated at N26; relative energy 8.90 kcal/mol). The weakest bond index is boldfaced; for simplicity the structure is presented for neutral form of **7**.

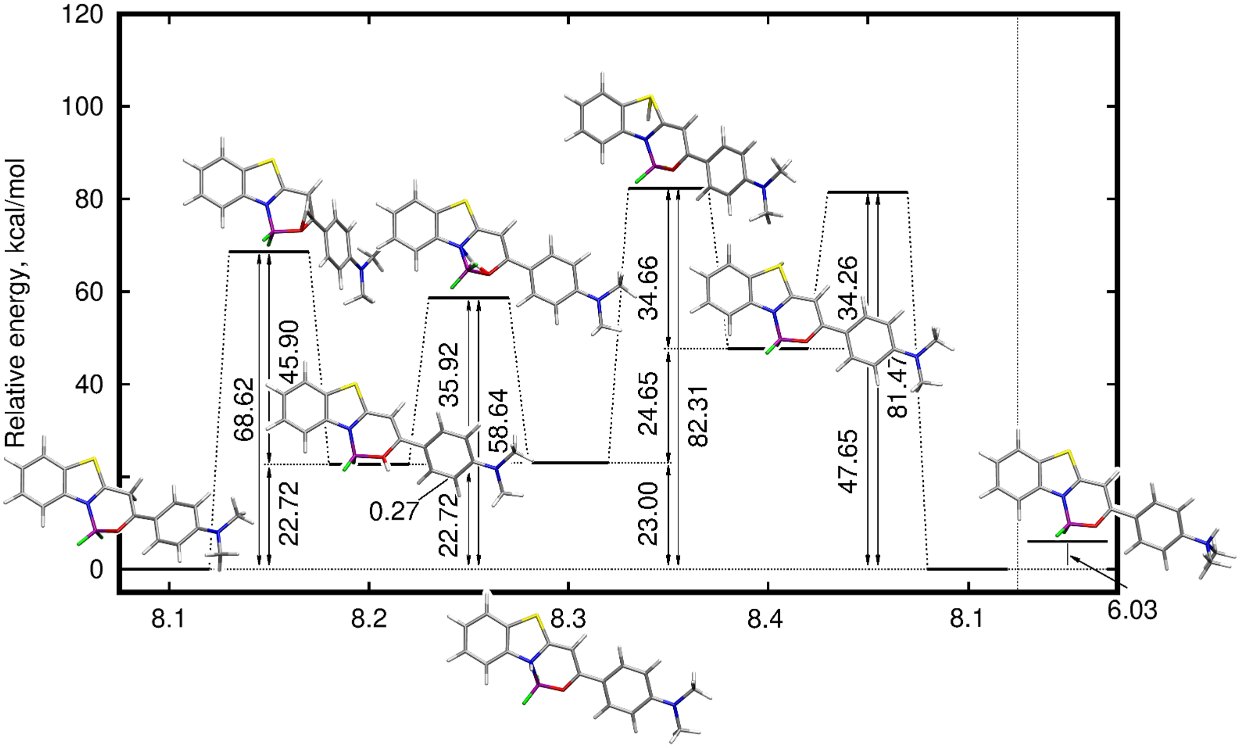

Figure S7: Mechanism of possible proton transfer processes in [**8**+H]$^+$ ion calculated within the ωB97X-D/6-311++G(d,p) approach

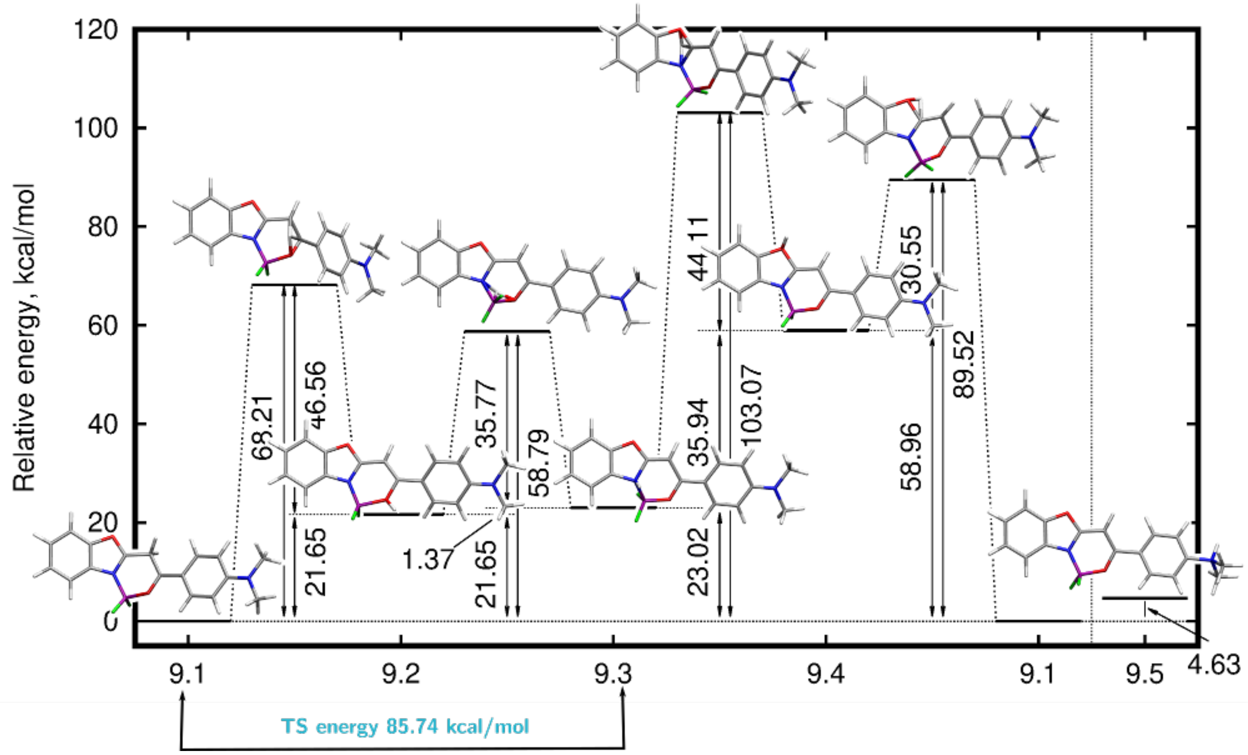

Figure S8: Mechanism of possible proton transfer processes in [**9**+H]$^+$ ion calculated within the ωB97X-D/6-311++G(d,p) approach (in blue transition state relative energy for proton transfer from N7 to C10 is given)

Table S11: Wiberg bond indexes for protonated ion [**1**+H]$^+$ (atom numbering in Fig. S1; the weakest bonds in a molecular core boldfaced; lowest energy in blue)

[**1**+H]$^+$	1.1	1.2	1.3	1.4	1.5
Relative energy	7.49	4.59	0.00	34.15	72.98
Protonation site X	N7	N7	N7	O13	O13
Protonation site Y	O13	C10	C10	C10	O13
C10-H23	0.9058	0.9037	0.8802	0.8900	0.9059
X-H29	0.7647	0.7761	0.7126	0.8844	0.6870
Y-H34	0.7434	0.8936	0.8802	0.7110	0.6742
C1-C6	1.2974	1.3304	1.3034	1.2847	1.2627
C1-N7	1.0760	1.0753	1.0825	1.1632	1.2154
N7-C8	1.2654	1.2982	1.3428	1.5523	1.5205
C8-N9	1.2618	1.3242	1.3006	1.1779	1.1359
C6-N9	1.0538	1.0568	1.0571	1.0737	1.1059
N9-C30	**0.9277**	**0.9163**	**0.9258**	**0.9380**	**0.9420**
C8-C10	1.1557	1.0321	1.0333	**0.9857**	1.0637
C10-C11	1.5916	**0.9262**	**0.9689**	1.0197	1.8113
C11-C12	1.0217	1.0513	1.0679	1.2109	1.0718
C11-O13	1.1107	1.7944	1.7309	1.3048	**0.6890**

Table S12: AIM parameters: electron density ρ, gradient of electron density $\nabla^2\rho$ and total energy density H_b according to Cremer and Kraka[S15] [a. u.] for selected bond critical points in protonated ions $[\mathbf{1}+H]^+$ (atom numbering in Fig. S1; lowest energy in blue)

$[\mathbf{1}+H]^+$		1.1			1.2			1.3			1.4			1.5	
Relative energy		7.49			4.59			0.00			34.15			72.98	
Protonation site X		N7			N7			N7			O13			O13	
Protonation site Y		O13			C10			C10			C10			O13	
	ρ	$\nabla^2\rho$	H_b	ρ	$\nabla^2\rho$	H_b	ρ	$\nabla^2\rho$	H_b	ρ	$\nabla^2\rho$	H_b	ρ	$\nabla^2\rho$	H_b
C10-H23	0.2778	-0.9355	-0.2761	0.2792	-0.9481	-0.2771	0.2734	-0.9122	-0.2680	0.2767	-0.9368	-0.2720	0.2784	-0.9526	-0.2764
X-H29	0.3653	-2.6098	-0.7180	0.2793	-0.9515	-0.2760	0.2733	-0.9118	-0.2679	0.2773	-0.9391	-0.2731	0.3507	-2.5933	-0.7015
Y-H34	0.3368	-1.8291	-0.5014	0.3380	-1.8013	-0.4946	0.3257	-1.8771	-0.5100	0.3582	-2.6168	-0.7130	0.3465	-2.5815	-0.6965
C1-C6	0.3182	-0.9164	-0.3325	0.3182	-0.9153	-0.3326	0.3183	-0.9153	-0.3327	0.3123	-0.8800	-0.3213	0.3107	-0.8743	-0.3182
C1-N7	0.2912	-0.6371	-0.4044	0.2890	-0.6061	-0.4017	0.2925	-0.6326	-0.4078	0.3063	-0.8660	-0.4026	0.3156	-0.8953	-0.4274
N7-C8	0.3307	-0.9006	-0.4782	0.3330	-0.8434	-0.4906	0.3419	-0.9012	-0.5065	0.3709	-1.1009	-0.5499	0.3681	-1.1077	-0.5394
C8-N9	0.3310	-0.8989	-0.4782	0.3383	-0.8639	-0.4999	0.3329	-0.8445	-0.4878	0.3156	-0.8032	-0.4451	0.3059	-0.7867	-0.4205
C6-N9	0.2887	-0.6647	-0.3947	0.2893	-0.6605	-0.3970	0.2892	-0.6466	-0.3978	0.2932	-0.6474	-0.4075	0.2988	-0.6464	-0.4205
N9-C30	0.2502	-0.5560	-0.3175	0.2437	-0.4862	-0.3112	0.2494	-0.5448	-0.3175	0.2564	-0.6215	-0.3217	0.2573	-0.6356	-0.3206
C8-C10	0.2825	-0.7487	-0.2754	0.2590	-0.6436	-0.2340	0.2574	-0.6407	-0.2267	0.2514	-0.6056	-0.2104	0.2743	-0.7203	-0.2500
C10-C11	0.3262	-0.9304	-0.3590	0.2350	-0.5187	-0.1831	0.4033	-0.0285	-0.6740	0.2623	-0.6730	-0.2364	0.3443	-1.0237	-0.4096
C11-C12	0.2713	-0.7163	-0.2452	0.2685	-0.6826	-0.2382	0.2716	-0.6999	-0.2447	0.2941	-0.8202	-0.2974	0.2754	-0.7357	-0.2663
C11-O13	0.3073	-0.2785	-0.4561	0.4123	0.0135	-0.6966	0.2437	-0.5643	-0.1952	0.3375	-0.1154	-0.5214	0.1725	-0.0549	-0.1874

Table S13: AIM parameters for the intramolecular hydrogen bonds in investigated neutral and protonated systems (V denotes virial field or potential energy density, G is the Lagrangian form of kinetic energy density in bond critical point (BCP), H_b stands for the negative value of hamiltonian form of kinetic energy density and E_{HB} represents the intramolecular hydrogen bond energy in kcal/mol;[S16] atom numbering in Figs. S1-S3)

System		BCP	Protonation sites	ρ	$\nabla^2\rho$	V	G	H_b	E_{HB}
**1**	Enol	N7...H29	O13	0.0510	0.1178	-0.0488	0.0391	-0.0097	15.30
**1**	Enamine	O13...H29	N7	0.0330	0.1187	-0.0270	0.0284	0.0013	8.48
**1**+H^+	1.3	O13...H29	N7, C10	0.0324	0.1213	-0.0266	0.0285	0.0018	8.36
**2**	Enol	N7...H29	O13	0.0484	0.1175	-0.0456	0.0375	-0.0081	14.31
**2**	Enamine	O13...H29	N7	0.0363	0.1276	-0.0310	0.0314	-0.0005	9.73
**2**+H^+	2.3	O13...H29	N7, C10	0.0402	0.1365	-0.0357	0.0349	-0.0008	11.20
**3**	Enol	N7...H29	O13	0.0446	0.1153	-0.0407	0.0348	-0.0059	12.77
**3**	Enamine	O13...H29	N7	0.0323	0.1172	-0.0261	0.0277	0.0016	8.19
**3**+H^+	3.3	O13...H29	N7, C10	0.0333	0.1214	-0.0273	0.0288	0.0015	8.53
**4**	Enol	N7...H29	O13	0.0516	0.1186	-0.0497	0.0397	-0.0100	15.60
**4**	Enamine	O13...H29	N7	0.0337	0.1209	-0.0279	0.0290	0.0012	8.74
**4**+H^+	4.3	O13...H29	N7, C10	0.0368	0.1313	-0.0316	0.0322	0.0006	9.92
**4**+H^+	4.6	O13...H29	N7, N26	0.0307	0.1130	-0.0246	0.0264	0.0018	7.71
**4**+H^+	4.7	N7...H29	O13, N26	0.0585	0.1168	-0.0583	0.0437	-0.0145	18.28
**5**	Enol	N7...H29	O13	0.0492	0.1182	-0.0466	0.0381	-0.0085	14.62
**5**	Enamine	O13...H29	N7	0.0371	0.1295	-0.0319	0.0321	0.0002	10.01
**5**+H^+	5.3	O13...H29	N7, C10	0.0480	0.1472	-0.0453	0.0410	-0.0042	14.21
**5**+H^+	5.6	O13...H29	N7, N26	0.0342	0.1228	-0.0286	0.0297	0.0010	8.97
**5**+H^+	5.7	N7...H29	O13, N26	0.0524	0.1170	-0.0504	0.0398	-0.0156	15.81
**6**	Enol	N7...H29	O13	0.0451	0.1162	-0.0416	0.0353	-0.0062	13.05
**6**	Enamine	O13...H29	N7	0.0332	0.1200	-0.0272	0.0286	0.0014	8.53
**6**+H^+	6.3	O13...H29	N7, C10	0.0396	0.1340	-0.0345	0.0340	-0.0005	10.82
**6**+H^+	6.6	O13...H29	N7, N26	0.0291	0.1082	-0.0228	0.0249	0.0021	7.15
**6**+H^+	6.7	N7...H29	O13, N26	0.0470	0.1150	-0.0436	0.0362	-0.0074	13.68

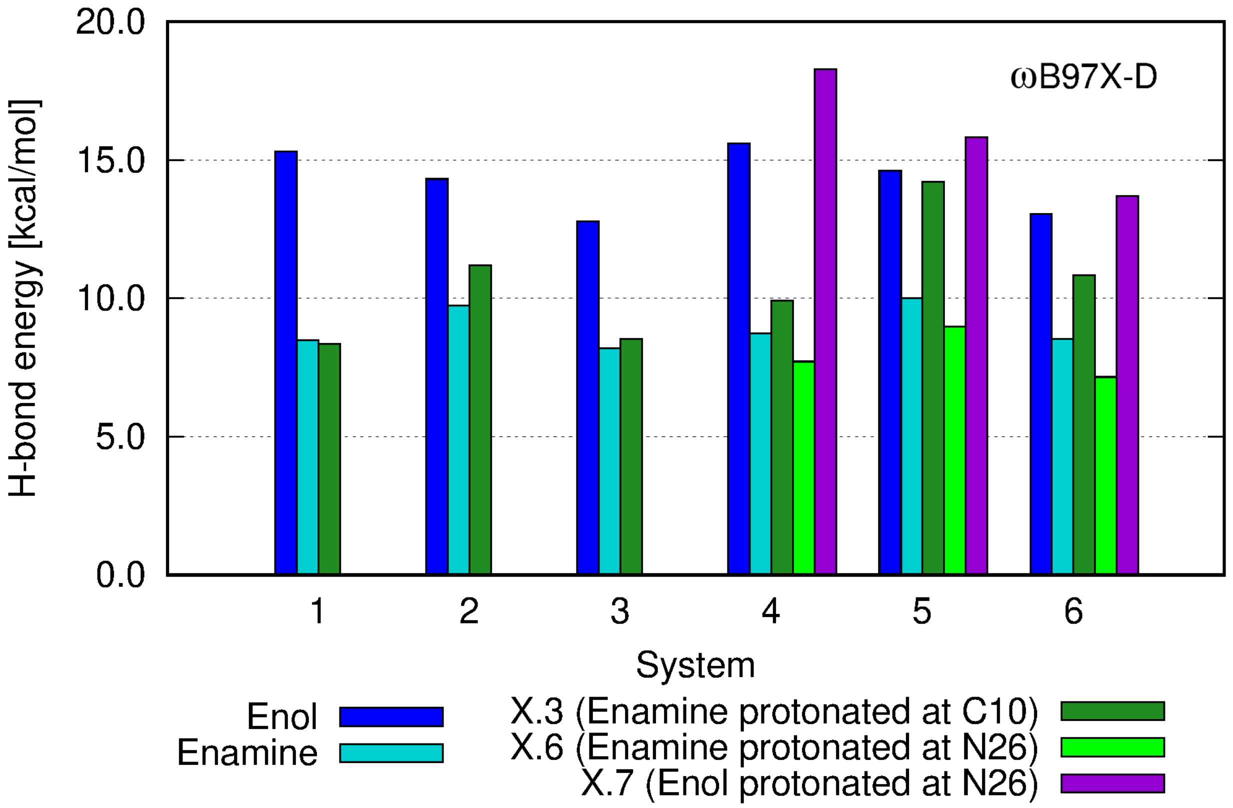

Figure S9: AIM intramolecular hydrogen bond energy in kcal/mol;[S16] atom numbering in Figs. S1-S3

Table S14: Wiberg bond indexes for protonated ion [**4**+H]$^+$ (atom numbering in Fig. S2; the weakest bonds in a molecular core boldfaced; lowest energy in blue)

[**4**+H]$^+$	4.1	4.2	4.3	4.4	4.5	4.6	4.7	4.8
Relative energy	71.06	24.19	0.00	2.92	8.86	17.61	21.94	23.22
Protonation site X	O13	C10	N7	N7	N7	N26	N26	N26
Protonation site Y	O13	O13	C10	C10	O13	N7	O13	C10
C10-H23	0.8952	0.8937	0.8839	0.9015	0.9071	0.9185	0.9173	0.8992
X-H29	0.7017	0.8909	0.8830	0.8882	0.7463	0.7769	0.7766	0.7750
Y-H34	0.6884	0.7278	0.6985	0.7802	0.7653	0.7216	0.5925	0.9028
C1-C6	1.2619	1.2818	1.3029	1.3043	1.2959	1.2846	1.2718	1.2755
C1-N7	1.2148	1.1661	1.0840	1.0744	1.0754	1.0807	1.1665	1.1748
N7-C8	1.5191	1.5662	1.3477	1.3036	1.2484	1.2293	1.4564	1.5601
C8-N9	1.1345	1.1661	1.2940	1.3248	1.2452	1.1828	1.1627	1.1665
C6-N9	1.1076	1.0773	1.0571	1.0565	1.0499	1.0534	1.0822	1.0871
N9-C30	**0.9422**	**0.9406**	**0.9270**	**0.9210**	**0.9303**	**0.9397**	**0.9431**	**0.9381**
C8-C10	1.0695	**0.9912**	1.0347	1.0340	1.1898	1.2916	1.1155	**0.9996**
C10-C11	1.7976	1.0139	**0.9601**	**0.9288**	1.5154	1.3104	1.6167	**0.9746**
C11-C12	1.1490	1.3691	1.1370	1.1173	1.0846	**0.9815**	1.0375	**0.9915**
C11-O13	**0.6014**	1.2008	1.6550	1.7152	1.0996	1.5088	1.1140	1.8174
C16-N26	1.2216	1.3150	1.2160	1.2086	1.2047	**0.8889**	**0.8910**	**0.8913**
N26-C32	**0.9508**	**0.9404**	**0.9506**	**0.9516**	**0.9512**	**0.8951**	**0.8942**	**0.8934**
N26-C36	**0.9497**	**0.9401**	**0.9522**	**0.9530**	**0.9528**	**0.8955**	**0.8947**	**0.8943**

Table S15: AIM parameters: electron density ρ, gradient of electron density $\nabla^2\rho$ and total energy density H_b according to Cremer and Kraka[S15] [a. u.] for selected bond critical points in [**4**+H]$^+$ ions protonated at the core (atom numbering in Fig. S2; lowest energy in blue)

[**4**+H]$^+$		4.1			4.2			4.3			4.4			4.5	
Relative energy		71.06			24.19			0.00			2.92			8.86	
Protonation site X		O13			C10			N7			N7			N7	
Protonation site Y		O13			O13			C10			C10			O13	
	ρ	$\nabla^2\rho$	H_b	ρ	$\nabla^2\rho$	H_b	ρ	$\nabla^2\rho$	H_b	ρ	$\nabla^2\rho$	H_b	ρ	$\nabla^2\rho$	H_b
C10-H23	0.2815	-0.9745	-0.2804	0.2761	-0.9284	-0.2719	0.2735	-0.9117	-0.2684	0.2758	-0.9262	-0.2720	0.2772	-0.9283	-0.2755
X-H29	0.3547	-2.6084	-0.7082	0.2774	-0.9384	-0.2733	0.2732	-0.9100	-0.2679	0.2737	-0.9132	-0.2685	0.3665	-2.6148	-0.7200
Y-H34	0.3502	-2.5841	-0.7001	0.3635	-2.6267	-0.7196	0.3210	-1.8571	-0.5052	0.3389	-1.7995	-0.4946	0.3369	-1.8240	-0.5005
C1-C6	0.3106	-0.8732	-0.3178	0.3125	-0.8822	-0.3218	0.3180	-0.9137	-0.3320	0.3186	-0.9179	-0.3334	0.3180	-0.9158	-0.3321
C1-N7	0.3158	-0.9007	-0.4258	0.3077	-0.8805	-0.4015	0.2935	-0.6466	-0.4089	0.2894	-0.6127	-0.4022	0.2918	-0.6533	-0.4043
N7-C8	0.3672	-1.1059	-0.5376	0.3713	-1.0996	-0.5515	0.3434	-0.9096	-0.5097	0.3334	-0.8350	-0.4926	0.3290	-0.9072	-0.4730
C8-N9	0.3057	-0.7878	-0.4206	0.3121	-0.7900	-0.4377	0.3319	-0.8405	-0.4859	0.3398	-0.8603	-0.5049	0.3287	-0.9025	-0.4719
C6-N9	0.2988	-0.6536	-0.4197	0.2951	-0.6609	-0.4105	0.2895	-0.6536	-0.3978	0.2903	-0.6665	-0.3989	0.2891	-0.6780	-0.3942
N9-C30	0.2574	-0.6377	-0.3202	0.2578	-0.6359	-0.3221	0.2504	-0.5537	-0.3185	0.2478	-0.5166	-0.3177	0.2517	-0.5678	-0.3195
C8-C10	0.2762	-0.7298	-0.2528	0.2522	-0.6086	-0.2108	0.2577	-0.6430	-0.2285	0.2626	-0.6692	-0.2389	0.2861	-0.7610	-0.2845
C10-C11	0.3440	-1.0288	-0.4081	0.2612	-0.6641	-0.2299	0.2405	-0.5497	-0.1906	0.2335	-0.5112	-0.1809	0.3200	-0.9025	-0.3450
C11-C12	0.2849	-0.7688	-0.2881	0.3117	-0.8868	-0.3317	0.2826	-0.7451	-0.2661	0.2805	-0.7351	-0.2619	0.2798	-0.7475	-0.2633
C11-O13	0.1478	-0.0471	-0.1207	0.3213	-0.1762	-0.4862	0.3958	-0.1017	-0.6566	0.4046	-0.0727	-0.6785	0.3044	-0.2832	-0.4501
C16-N26	0.3227	-0.8785	-0.4576	0.3331	-0.8438	-0.4889	0.3215	-0.8706	-0.4557	0.3205	-0.8708	-0.4530	0.3205	-0.8798	-0.4511
N26-C32	0.2589	-0.6691	-0.3106	0.2535	-0.6329	-0.3061	0.2597	-0.6739	-0.3110	0.2594	-0.6723	-0.3107	0.2592	-0.6702	-0.3107
N26-C36	0.2584	-0.6657	-0.3102	0.2535	-0.6318	-0.3064	0.2589	-0.6689	-0.3102	0.2601	-0.6764	-0.3114	0.2600	-0.6753	-0.3114

Table S16: AIM parameters: electron density ρ, gradient of electron density $\nabla^2\rho$ and total energy density H_b according to Cremer and Kraka[S15] [a. u.] for selected bond critical points in [**4**+H]$^+$ ions protonated at the dimethylamino group (atom numbering in Fig. S2)

[**4**+H]$^+$	4.6			4.7			4.8		
Relative energy	17.61			21.94			23.33		
Protonation site X	N26			N26			N26		
Protonation site Y	N7			O13			C10		
	ρ	$\nabla^2\rho$	H_b	ρ	$\nabla^2\rho$	H_b	ρ	$\nabla^2\rho$	H_b
C10-H23	0.2775	-0.9264	-0.2780	0.2802	-0.9527	-0.2810	0.2771	-0.9350	-0.2736
X-H29	0.3261	-1.8388	-0.5030	0.3130	-2.1610	-0.6072	0.2784	-0.9434	-0.2749
Y-H34	0.3425	-1.7491	-0.4832	0.3424	-1.7499	-0.4833	0.3422	-1.7536	-0.4839
C1-C6	0.3153	-0.9022	-0.3266	0.3122	-0.8821	-0.3207	0.3120	-0.8801	-0.3206
C1-N7	0.2960	-0.7185	-0.4078	0.3099	-0.8741	-0.4131	0.3100	-0.8933	-0.4044
N7-C8	0.3295	-0.9444	-0.4699	0.3580	-1.0867	-0.5156	0.3678	-1.0881	-0.5445
C8-N9	0.3215	-0.8975	-0.4538	0.3117	-0.8092	-0.4365	0.3106	-0.7820	-0.4356
C6-N9	0.2901	-0.7056	-0.3925	0.2961	-0.6783	-0.4112	0.2977	-0.6816	-0.4147
N9-C30	0.2582	-0.6273	-0.3258	0.2593	-0.6469	-0.3238	0.2559	-0.6207	-0.3200
C8-C10	0.2989	-0.8183	-0.3159	0.2813	-0.7461	-0.2630	0.2540	-0.6150	-0.2145
C10-C11	0.3008	-0.8121	-0.3012	0.3288	-0.9430	-0.3647	0.2501	-0.5931	-0.2074
C11-C12	0.2552	-0.6280	-0.2139	0.2705	-0.7077	-0.2386	0.2567	-0.6276	-0.2148
C11-O13	0.3801	-0.2763	-0.6208	0.3142	-0.3699	-0.4704	0.4117	0.0398	-0.6944
C16-N26	0.2442	-0.5843	-0.2959	0.2450	-0.5908	-0.2965	0.2459	-0.6065	-0.2957
N26-C32	0.2366	-0.5537	-0.2739	0.2361	-0.5506	-0.2733	0.2356	-0.5462	-0.2731
N26-C36	0.2369	-0.5555	-0.2743	0.2364	-0.5524	-0.2738	0.2359	-0.5475	-0.2737

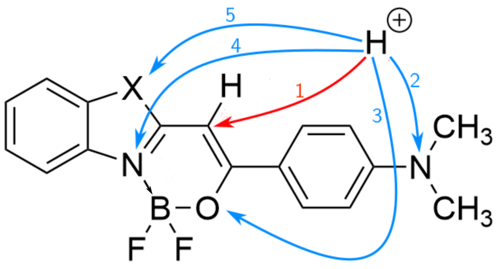

where X=N(CH_3), S, O

Figure S10: The energetically favourable protonation sites in compounds **7-9**. The numbers above the arrows refer to the protonation sites indicated from most to least beneficial.

Table S17: Relative energy ΔE, proton affinity (PA) and gas phase basicity (GB) for protonated systems [**7**+H]$^+$, [**8**+H]$^+$ and [**9**+H]$^+$ [kcal/mol] (boldfaced most stable protonated isomer and largest proton affinity)

	[**7**+H]$^+$			[**8**+H]$^+$			[**9**+H]$^+$		
Protonation site	ΔE	PA	GB	ΔE	PA	GB	ΔE	PA	GB
N7	23.28	211.22	202.55	23.00	206.30	198.40	23.02	204.54	196.59
N9/S9/O9	35.54	199.04	190.78	47.65	184.21	176.90	58.96	169.62	162.15
C10	**0.00**	**234.32**	**225.86**	**0.00**	**229.49**	**221.06**	**0.00**	**227.26**	**218.45**
O13	21.58	213.33	205.73	22.72	207.13	199.04	21.65	206.11	198.08
N26	8.91	224.78	216.42	6.03	222.55	213.97	4.63	221.88	213.33

Table S18: Wiberg bond indexes for protonated ion [**7**+H]$^+$ (atom numbering in Fig. S3; the weakest bonds in a molecular core boldfaced; lowest energy in blue)

[**7**+H]$^+$	7.1	7.2	7.3	7.4	7.5
Relative energy	0.00	21.58	23.28	35.54	8.90
Protonation site X	C10	O13	N7	N9	N26
C10-H23	0.9014	0.8952	0.8954	0.9073	0.9125
X-H44	0.8574	0.6759	0.7491	0.7544	0.7767
C1-C6	1.3040	1.2988	1.3252	1.3281	1.2944
C1-N7	1.0805	1.0666	**0.9292**	1.0331	1.0870
N7-C8	1.3704	1.2732	**0.9485**	1.2957	1.3159
C8-N9	1.2759	1.2591	1.2319	**0.8765**	1.2195
C6-N9	1.0563	1.0564	1.0003	**0.8883**	1.0585
N9-C40	**0.9303**	1.2591	**0.9271**	**0.8787**	**0.9379**
C8-C10	1.0234	1.1391	1.4938	1.5151	1.1504
C10-C11	**0.9993**	1.6247	1.2215	1.1858	1.5559
C11-C12	1.2943	1.1296	1.2378	1.1985	1.0261
C11-O13	1.3291	**0.8890**	1.1531	1.2428	1.1588
O13-B29	**0.5574**	**0.3830**	**0.6443**	**0.6042**	**0.6095**
N7-B29	**0.6050**	**0.6551**	**0.4631**	**0.5831**	**0.6136**
B29-F30	**0.7799**	**0.7940**	**0.7781**	**0.7640**	**0.7512**
B29-F31	**0.7612**	**0.8084**	**0.7763**	**0.7539**	**0.7378**
C16-N26	1.2840	1.2188	1.2575	1.2391	**0.8920**
N26-C32	**0.9445**	**0.9502**	**0.9462**	**0.9479**	**0.8936**
N26-C36	**0.9429**	**0.9505**	**0.9476**	**0.9499**	**0.8943**

Table S19: AIM parameters: electron density ρ, gradient of electron density $\nabla^2\rho$ and total energy density H_b according to Cremer and Kraka[S15] in bond critical points for the corresponding bonds [a. u.] for selected bond critical points in protonated [**7**+H]$^+$ ions (atom numbering in Fig. S3; lowest energy in blue)

[**7**+H]$^+$		7.1			7.2			7.3			7.4			7.5	
Relative energy		0.00			21.58			23.28			35.54			8.90	
Protonation site X		C10			O13			N7			N9			N26	
	ρ	$\nabla^2\rho$	H_b	ρ	$\nabla^2\rho$	H_b	ρ	$\nabla^2\rho$	H_b	ρ	$\nabla^2\rho$	H_b	ρ	$\nabla^2\rho$	H_b
C10-H23	0.2800	-0.9544	-0.2788	0.2823	-0.9728	-0.2821	0.2817	-0.9656	-0.2820	0.2807	-0.9570	-0.2809	0.2804	-0.9534	-0.2810
X-H44	0.2709	-0.9002	-0.2633	0.3530	-2.5927	-0.7048	0.3304	-1.6460	-0.4593	0.3371	-1.7481	-0.4808	0.3424	-1.7503	-0.4834
C1-C6	0.3172	-0.9087	-0.3305	0.3180	-0.9147	-0.3320	0.3220	-0.9330	-0.3406	0.3208	-0.9242	-0.3391	0.3166	-0.9072	-0.3292
C1-N7	0.2934	-0.7010	-0.4022	0.2904	-0.6936	-0.3953	0.2586	-0.6385	-0.3185	0.2936	-0.8057	-0.3870	0.2975	-0.7530	-0.4058
N7-C8	0.3518	-0.9618	-0.5234	0.3347	-0.9478	-0.4798	0.2750	-0.7837	-0.3145	0.3517	-1.0578	-0.5071	0.3442	-0.9903	-0.4997
C8-N9	0.3302	-0.8298	-0.4811	0.3304	-0.8777	-0.4782	0.3351	-0.9517	-0.4812	0.2472	-0.6425	-0.2739	0.3243	-0.8610	-0.4644
C6-N9	0.2888	-0.6339	-0.3983	0.2902	-0.6572	-0.3995	0.2817	-0.7147	-0.3716	0.2429	-0.5313	-0.2972	0.2912	-0.6685	-0.4004
N9-C40	0.2518	-0.5713	-0.3195	0.2514	-0.5637	-0.3196	0.2523	-0.5623	-0.3220	0.2291	-0.5015	-0.2657	0.2564	-0.6126	-0.3240
C8-C10	0.2602	-0.6510	-0.2270	0.2848	-0.7637	-0.2756	0.3202	-0.9113	-0.3655	0.3224	-0.9212	-0.3724	0.2877	-0.7766	-0.2815
C10-C11	0.2512	-0.6022	-0.2078	0.3331	-0.9723	-0.3782	0.2924	-0.7842	-0.2845	0.2896	-0.7735	-0.2777	0.3260	-0.9324	-0.3576
C11-C12	0.3028	-0.8444	-0.3111	0.2842	-0.7659	-0.2779	0.2981	-0.8288	-0.2999	0.2805	-0.7351	-0.2619	0.2681	-0.6959	-0.2340
C11-O13	0.3510	-0.1293	-0.5506	0.2506	-0.0692	-0.3389	0.3209	-0.2503	-0.4847	0.3352	-0.1702	-0.5154	0.3267	-0.2651	-0.4970
O13-B29	0.1253	0.4690	-0.0801	0.0833	0.2673	-0.0473	0.1541	0.5604	-0.1109	0.1369	0.5251	-0.0906	0.1396	0.5340	-0.0934
N7-B29	0.1446	0.3027	-0.1166	0.1639	0.2729	-0.1433	0.1107	0.1985	-0.0821	0.1376	0.3163	-0.1073	0.1437	0.3093	-0.1151
B29-F30	0.1827	0.9994	-0.1178	0.1862	1.0248	-0.1214	0.1816	0.9866	-0.1174	0.1778	0.9577	-0.1134	0.1691	0.8853	-0.1063
B29-F31	0.1757	0.9310	-0.1128	0.1891	1.0329	-0.1257	0.1828	1.0013	-0.1180	0.1742	0.9186	-0.1114	0.1734	0.9303	-0.1090
C16-N26	0.3297	-0.8543	-0.4792	0.3226	-0.8804	-0.4567	0.3264	-0.8580	-0.4702	0.3242	-0.8632	-0.4640	0.2455	-0.5985	-0.2962
N26-C32	0.2549	-0.6424	-0.3073	0.2586	-0.6662	-0.3105	0.2567	-0.6541	-0.3088	0.2575	-0.6597	-0.3093	0.2359	-0.5486	-0.2735
N26-C36	0.2557	-0.6481	-0.3080	0.2587	-0.6673	-0.3106	0.2573	-0.6585	-0.3094	0.2585	-0.6662	-0.3101	0.3125	-0.8795	-0.3259

Table S20: Wiberg bond indexes for protonated ion [**8**+H]$^+$ (atom numbering in Fig. S3; the weakest bonds in a molecular core boldfaced; lowest energy in blue)

[**8**+H]$^+$	8.1	8.2	8.3	8.4	8.5
Relative energy	0.00	22.72	23.00	47.65	6.03
Protonation site X	C10	O13	N7	S9	N26
C10-H23	0.9029	0.8920	0.8959	0.9026	0.9096
X-H40	0.8490	0.6724	0.7483	0.9078	0.7763
C1-C6	1.3244	1.3244	1.3590	1.3206	1.3257
C1-N7	1.0616	1.0469	**0.9220**	1.0503	1.0623
N7-C8	1.4506	1.3256	**0.9409**	1.2722	1.3744
C8-S9	1.2898	1.2570	1.1758	**0.9598**	1.2201
C6-S9	1.1063	1.0983	1.0393	1.0031	1.0954
C8-C10	1.0305	1.1871	1.6561	1.5519	1.1904
C10-C11	**0.9981**	1.5791	1.1324	1.1617	1.5313
C11-C12	1.3187	1.1556	1.2934	1.2179	1.0276
C11-O13	1.2928	**0.8812**	1.1785	1.2392	1.1607
O13-B29	**0.5807**	**0.4068**	**0.6315**	**0.6107**	**0.6192**
N7-B29	**0.5688**	**0.6335**	**0.4764**	**0.5658**	**0.5892**
B29-F30	**0.7623**	**0.8108**	**0.7795**	**0.7565**	**0.7506**
B29-F31	**0.7796**	**0.7881**	**0.7669**	**0.7603**	**0.7428**
C16-N26	1.2944	1.2316	1.2891	1.2506	**0.8924**
N26-C32	**0.9421**	**0.9489**	**0.9430**	**0.9468**	**0.8938**
N26-C36	**0.9432**	**0.9490**	**0.9440**	**0.9484**	**0.8933**

Table S21: AIM parameters: electron density ρ, gradient of electron density $\nabla^2\rho$ and total energy density H_b according to Cremer and Kraka[S15] in bond critical points for the corresponding bonds [a. u.] for selected bond critical points in protonated [**8**+H]$^+$ ions (atom numbering in Fig. S3; lowest energy in blue)

[**8**+H]$^+$		8.1			8.2			8.3			8.4			8.5	
Relative energy		0.00			22.72			23.00			47.65			6.03	
Protonation site X		C10			O13			N7			S9			N26	
	ρ	$\nabla^2\rho$	H_b	ρ	$\nabla^2\rho$	H_b	ρ	$\nabla^2\rho$	H_b	ρ	$\nabla^2\rho$	H_b	ρ	$\nabla^2\rho$	H_b
C10-H23	0.2815	-0.9666	-0.2804	0.2825	-0.9771	-0.2818	0.2836	-0.9843	-0.2837	0.2826	-0.9730	-0.2831	0.2812	-0.9611	-0.2813
X-H40	0.2708	-0.9011	-0.2630	0.3521	-2.5903	-0.7036	0.3303	-1.6328	-0.4564	0.2187	-0.6584	-0.1976	0.3424	-1.7514	-0.4836
C1-C6	0.3090	-0.8509	-0.3150	0.3105	-0.8605	-0.3183	0.3154	-0.8815	-0.3289	0.3076	-0.8418	-0.3137	0.3102	-0.8585	-0.3176
C1-N7	0.2892	-0.6978	-0.3949	0.2865	-0.6964	-0.3881	0.2548	-0.6406	-0.3097	0.2957	-0.8185	-0.3927	0.2930	-0.7524	-0.3973
N7-C8	0.3555	-0.8455	-0.5417	0.3377	-0.8747	-0.4948	0.2674	-0.7269	-0.2981	0.3432	-1.0112	-0.4851	0.3465	-0.9024	-0.5144
C8-S9	0.2191	-0.4741	-0.1974	0.2167	-0.4650	-0.1911	0.2089	-0.4307	-0.1786	0.1772	-0.2726	-0.1127	0.2130	-0.4396	-0.1797
C6-S9	0.2043	-0.3781	-0.1543	0.2042	-0.3804	-0.1545	0.1956	-0.3488	-0.1414	0.1904	-0.3143	-0.1335	0.2029	-0.3743	-0.1526
C8-C10	0.2568	-0.6291	-0.2220	0.2873	-0.7684	-0.2807	0.3328	-0.9673	-0.3826	0.3232	-0.9236	-0.3716	0.2897	-0.7809	-0.2854
C10-C11	0.2533	-0.6115	-0.2118	0.3313	-0.9675	-0.3751	0.2839	-0.7546	-0.2667	0.2877	-0.7676	-0.2740	0.3260	-0.9365	-0.3583
C11-C12	0.3056	-0.8581	-0.3175	0.2873	-0.7797	-0.2850	0.3045	-0.8576	-0.3129	0.2950	-0.8131	-0.2937	0.2686	-0.6983	-0.2348
C11-O13	0.3461	-0.1497	-0.5399	0.2483	-0.0525	-0.3346	0.3270	-0.2290	-0.4980	0.3355	-0.1580	-0.5160	0.3270	-0.2353	-0.4975
O13-B29	0.1328	0.4908	-0.0878	0.0891	0.3075	-0.0505	0.1505	0.5579	-0.1062	0.1390	0.5389	-0.0923	0.1428	0.5504	-0.0965
N7-B29	0.1347	0.2698	-0.1061	0.1587	0.2546	-0.1373	0.1141	0.1976	-0.0859	0.1336	0.2910	-0.1037	0.1381	0.2857	-0.1094
B29-F30	0.1764	0.9369	-0.1134	0.1900	1.0387	-0.1266	0.1830	0.9966	-0.1188	0.1751	0.9249	-0.1124	0.1739	0.9319	-0.1096
B29-F31	0.1835	1.0074	-0.1185	0.1851	1.0146	-0.1205	0.1814	0.9912	-0.1167	0.1772	0.9531	-0.1130	0.1710	0.9004	-0.1081
C16-N26	0.3308	-0.8505	-0.4825	0.3241	-0.8775	-0.4614	0.3299	-0.8479	-0.4805	0.3256	-0.8600	-0.4680	0.2459	-0.6027	-0.2962
N26-C32	0.2550	-0.6433	-0.3074	0.2580	-0.6619	-0.3102	0.2554	-0.6453	-0.3079	0.2578	-0.6614	-0.3097	0.2356	-0.5463	-0.2730
N26-C36	0.2545	-0.6395	-0.3071	0.2580	-0.6620	-0.3102	0.2548	-0.6418	-0.3073	0.2569	-0.6557	-0.3088	0.2357	-0.5469	-0.2733

- - - - -

Table S22: Wiberg bond indexes for protonated ion [**9**+H]$^+$ (atom numbering in Fig. S3; the weakest bonds in a molecular core boldfaced; lowest energy in blue)

[**9**+H]$^+$	9.1	9.2	9.3	9.4	9.5
Relative energy	0.00	21.65	23.02	58.96	4.63
Protonation site X	C10	O13	N7	O9	N26
C10-H23	0.8881	0.8877	0.8924	0.8996	0.9056
X-H40	0.8547	0.6759	0.7468	0.6625	0.7762
C1-C6	1.3298	1.3239	1.3492	1.3341	1.3230
C1-N7	1.0459	1.0345	**0.9265**	1.0384	1.0540
N7-C8	1.4787	1.3274	**0.9362**	1.3085	1.3755
C8-O9	1.1440	1.1270	1.0771	**0.7713**	1.0997
C6-O9	**0.9444**	**0.9459**	**0.9152**	**0.7659**	**0.9508**
C8-C10	1.0293	1.1896	1.6535	1.5206	1.1864
C10-C11	**0.9981**	1.5638	1.1369	1.1729	1.5263
C11-C12	1.3206	1.1627	1.2978	1.2144	1.0263
C11-O13	1.2997	**0.8964**	1.1727	1.2313	1.1749
O13-B29	**0.5731**	**0.3737**	**0.6308**	**0.6080**	**0.6062**
N7-B29	**0.5766**	**0.6442**	**0.4709**	**0.5557**	**0.5998**
B29-F30	**0.7708**	**0.8183**	**0.7845**	**0.7655**	**0.7580**
B29-F31	**0.7839**	**0.8026**	**0.7797**	**0.7681**	**0.7475**
C16-N26	1.2965	1.2373	1.2899	1.2487	**0.8928**
N26-C32	**0.9419**	**0.9483**	**0.9429**	**0.9469**	**0.8936**
N26-C36	**0.9430**	**0.9482**	**0.9438**	**0.9486**	**0.8932**

Table S23: AIM parameters: electron density ρ, gradient of electron density $\nabla^2\rho$ and total energy density H_b according to Cremer and Kraka[S15] in bond critical points for the corresponding bonds [a. u.] for selected bond critical points in protonated [**9**+H]$^+$ ions (atom numbering in Fig. S3; lowest energy in blue)

[**9**+H]$^+$		9.1			9.2			9.3			9.4			9.5	
Relative energy		0.00			21.65			23.02			58.96			4.63	
Protonation site X		C10			O13			N7			O9			N26	
	ρ	$\nabla^2\rho$	H_b	ρ	$\nabla^2\rho$	H_b	ρ	$\nabla^2\rho$	H_b	ρ	$\nabla^2\rho$	H_b	ρ	$\nabla^2\rho$	H_b
C10-H23	0.2788	-0.9509	-0.2756	0.2826	-0.9807	-0.2818	0.2831	-0.9832	-0.2828	0.2808	-0.9613	-0.2804	0.2814	-0.9644	-0.2812
X-H40	0.2710	-0.9026	-0.2636	0.3529	-2.5925	-0.7047	0.3298	-1.6380	-0.4571	0.3491	-2.6003	-0.7018	0.3424	-1.7517	-0.4836
C1-C6	0.3242	-0.9444	-0.3445	0.3247	-0.9487	-0.3453	0.3274	-0.9585	-0.3515	0.3258	-0.9594	-0.3525	0.3238	-0.9441	-0.3437
C1-N7	0.2857	-0.6731	-0.3862	0.2833	-0.6673	-0.3805	0.2569	-0.6263	-0.3155	0.2931	-0.7852	-0.3887	0.2908	-0.7313	-0.3910
N7-C8	0.3695	-0.9753	-0.5699	0.3476	-0.9939	-0.5101	0.2756	-0.7760	-0.3078	0.3625	-1.1415	-0.5233	0.3575	-1.0282	-0.5326
C8-O9	0.3192	-0.2211	-0.4829	0.3177	-0.2847	-0.4800	0.3149	-0.3960	-0.4723	0.2126	-0.1893	-0.2626	0.3112	-0.2803	-0.4657
C6-O9	0.2609	-0.0667	-0.3594	0.2639	-0.0829	-0.3654	0.2603	-0.1807	-0.3579	0.2015	0.0313	-0.2447	0.2655	-0.1071	-0.3685
C8-C10	0.2662	-0.6894	-0.2413	0.2947	-0.8179	-0.3015	0.3365	-0.9930	-0.4055	0.3237	-0.9186	-0.3968	0.2963	-0.8264	-0.3040
C10-C11	0.2510	-0.6024	-0.2072	0.3289	-0.9558	-0.3696	0.2825	-0.7454	-0.2642	0.2864	-0.7542	-0.2719	0.3241	-0.9254	-0.3539
C11-C12	0.3059	-0.8593	-0.3185	0.2883	-0.7842	-0.2874	0.3051	-0.8608	-0.3148	0.2945	-0.8112	-0.2936	0.2684	-0.6981	-0.2344
C11-O13	0.1356	-0.1276	-0.5384	0.2515	-0.0806	-0.3408	0.3250	-0.2406	-0.4936	0.3333	-0.1792	-0.5114	0.3280	-0.2365	-0.4996
O13-B29	0.1294	0.4897	-0.0836	0.0809	0.2403	-0.0465	0.1497	0.5489	-0.1057	0.1380	0.5259	-0.0919	0.1379	0.5255	-0.0918
N7-B29	0.1356	0.3013	-0.1054	0.1607	0.2782	-0.1386	0.1123	0.2012	-0.0837	0.1297	0.3047	-0.0982	0.1393	0.3107	-0.1095
B29-F30	0.1790	0.9516	-0.1162	0.1922	1.0557	-0.1288	0.1833	0.9991	-0.1192	0.1779	0.9427	-0.1150	0.1757	0.9409	-0.1116
B29-F31	0.1842	1.0033	-0.1198	0.1887	1.0412	-0.1241	0.1838	1.0058	-0.1192	0.1794	0.9653	-0.1153	0.1724	0.9082	-0.1095
C16-N26	0.3310	-0.8501	-0.4831	0.3249	-0.8769	-0.4635	0.3300	-0.8482	-0.4808	0.3254	-0.8615	-0.4674	0.2460	-0.6048	-0.2961
N26-C32	0.2543	-0.6382	-0.3069	0.2576	-0.6599	-0.3099	0.2548	-0.6417	-0.3072	0.2570	-0.6563	-0.3089	0.2356	-0.5459	-0.2731
N26-C36	0.2550	-0.6426	-0.3075	0.2576	-0.6595	-0.3100	0.2553	-0.6449	-0.3079	0.2579	-0.6622	-0.3098	0.2354	-0.5454	-0.2729

- - - - -

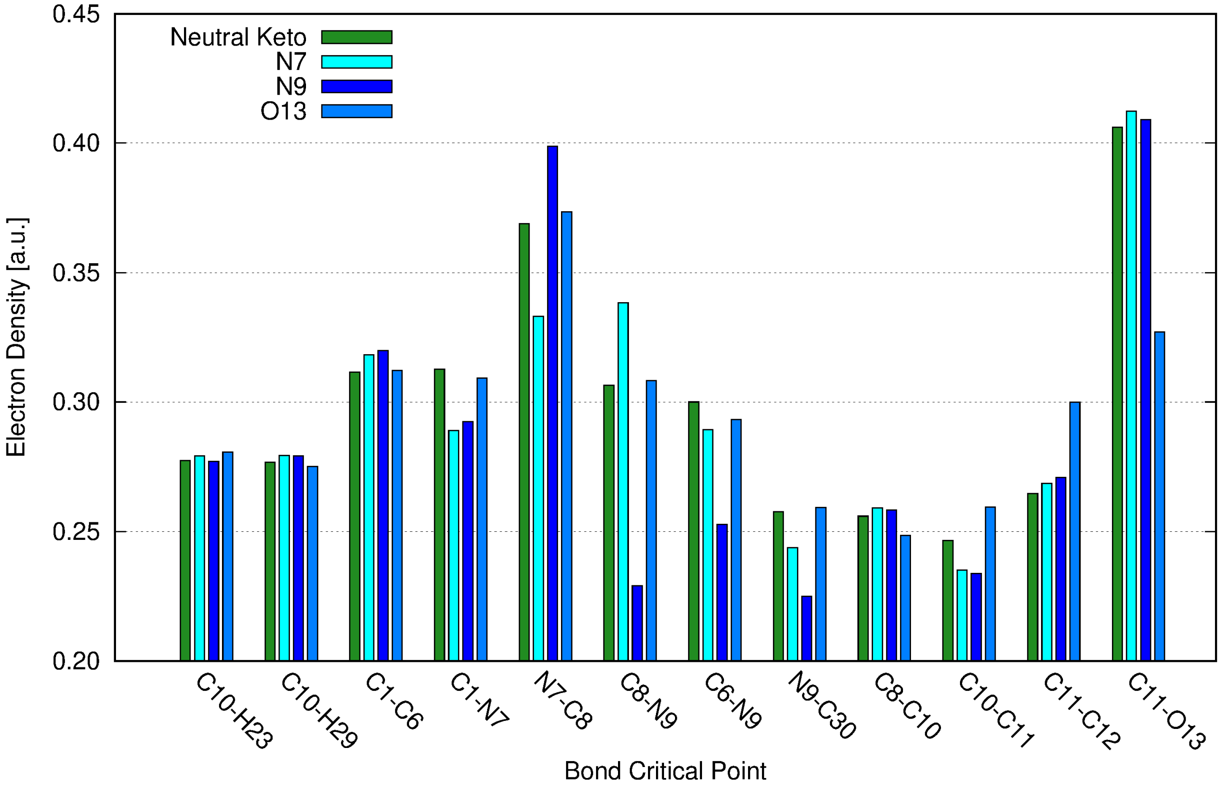

Figure S11: Modification of the AIM electron density ρ at bond critical points upon protonation at various sites for neutral keto form of system **1** (green) and its protonated counterparts (respectively at N7, N9 and O13 for cyan, dark blue and light blue boxes)

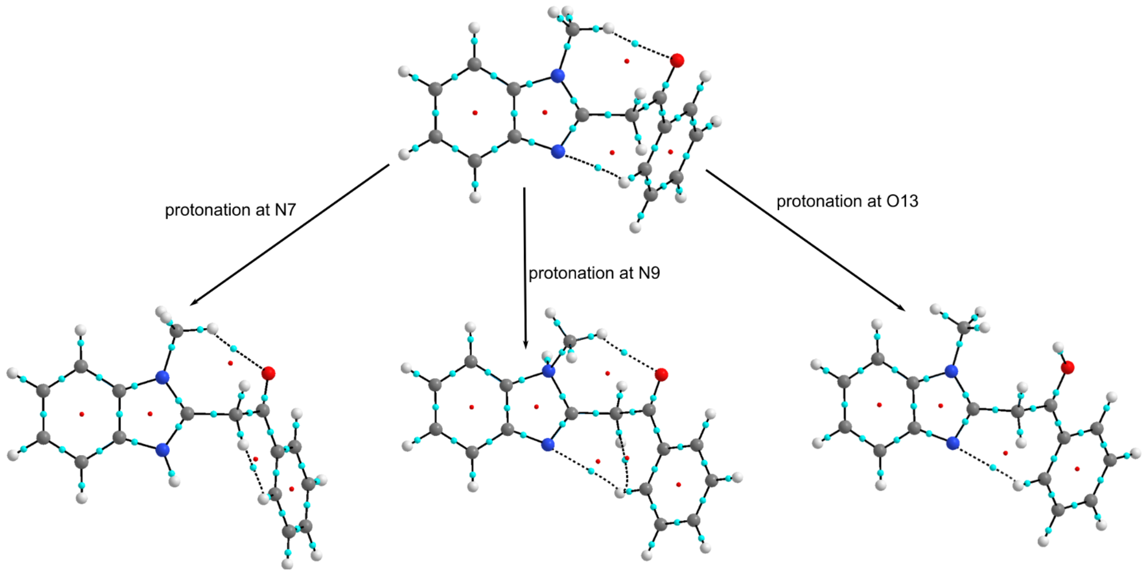

Figure S12: Molecular graphs for modification of the AIM electron density ρ at bond critical points for keto tautomer of **1** (upper panel) upon protonation at various sites (lower panel). Cyan balls depict bond critical points, red balls – ring critical points.

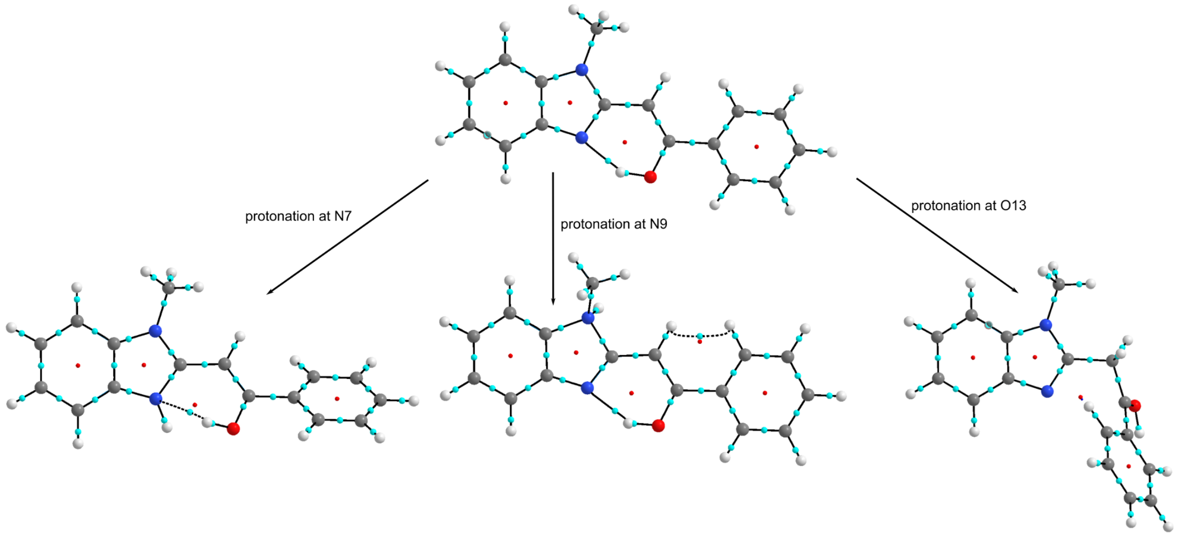

Figure S13: Molecular graphs for modification of the AIM electron density ρ at bond critical points for enol tautomer of **1** (upper panel) upon protonation at various sites (lower panel). Cyan balls depict bond critical points, red balls – ring critical points.

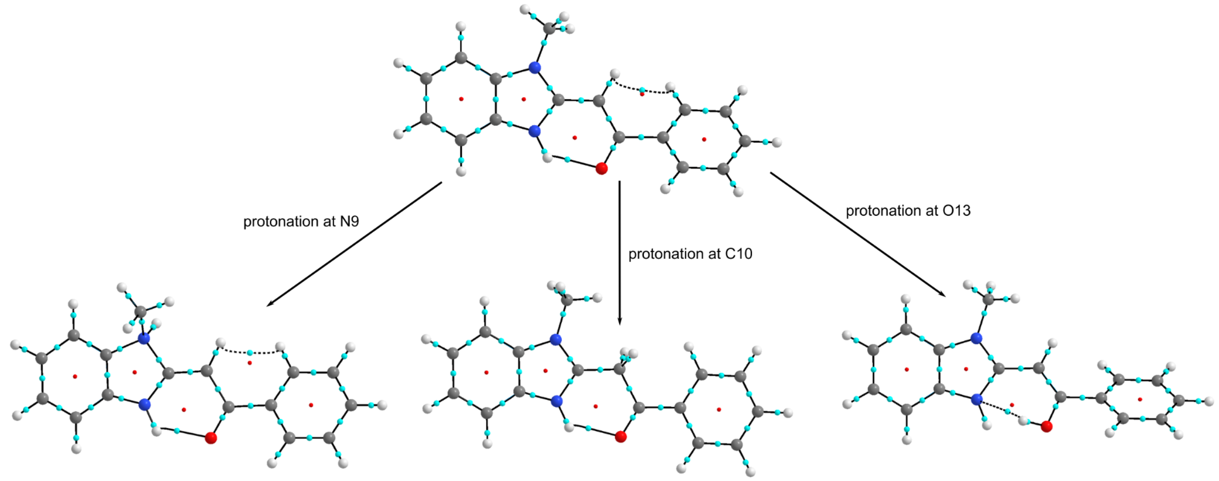

Figure S14: Molecular graphs for modification of the AIM electron density $\boldsymbol{\rho}$ at bond critical points for enamino tautomer of **1** (upper panel) upon protonation at various sites (lower panel). Cyan balls depict bond critical points, red balls – ring critical points.

References

(S1) Qi, Y. L.; Geib, T.; Huynh, A. M.; Jung, G.; Volmer, D. A. Fragmentation Patterns of Boron-Dipyrromethene (BODIPY) Dyes by Electrospray Ionization High-Resolution Tandem Mass Spectrometry. *Rapid Commun. Mass Spectrom.* **2015**, *29*, 885–890.

(S2) Kaczorowska, M. A.; Jędrzejewska, B. Electrospray Ionization Collision Induced Dissociation of Thio- and Selenocarbocyanine Dyes. *J. Mass Spectrom.* **2019**, *54*, 592–597.

(S3) Frański, R.; Gierczyk, B.; Zalas, M.; Jankowski, W.; Hoffman, M. Methyl Group Transfer Upon Gas Phase Decomposition of Protonated Methyl Benzoate and Similar Compounds. *J. Mass Spectrom.* **2018**, *53*, 379–384.

(S4) Liu, X.; Yang, J. L.; Li, J. H.; Li, X. L.; Li, J.; Lu, X. Y.; Shen, J. Z.; Wang, Y. W.; Zhang, Z. H. Analysis of Water-Soluble Azo Dyes in Soft Drinks by High Resolution UPLC-MS. *Food Addit. Contam. A* **2011**, *28*, 1315–1323.

(S5) Jiang, K.; Bian, G.; Hu, N.; Pan, Y.; Lai, G. Coordinated Dissociative Proton Transfers of External Proton and Thiocarbamide Hydrogen: MS Experimental and Theoretical Studies on the fragmentation of Protonated S-methyl Benzenylmethylenehydrazine Dithiocarboxylate in Gas Phase. *Int. J. Mass Spectrom.* **2010**, *291*, 17–23.

(S6) Kaczorowska, M. A.; Ośmiałowski, B. Collision Induced Dissociation of N-(pyridin-2-yl)-substituted benzo(thio)amides and N-(isoquinolin-1-yl)furan(thiophene)-2-carboxamides and Their Difluoroboranyl Derivatives. *Int. J. Mass Spectrom.* **2018**, *428*, 35–42.

(S7) Qi, P.; Liang, Z. A.; Wang, N.; Zhao, J. L.; Chen, Y. Z.; q. Zhou, Q.; Gao, H.; Jiang, J. J. How Does Azo Bond Cleave in the Gas Phase? Computational and Experimental Study on the Fragmentation Mechanism of Protonated Sudan I. *Chem. Select.* **2019**, *4*, 1666–1672.

(S8) Rifai, A.; Bourcier, S.; Jaber, F.; Bouchoux, G. Structures and Dissociation Mechanisms of Protonated and Electron Ionized Methamidophos. *Int. J. Mass Spectrom.* **2013**, *339-340*, 7–15.

(S9) Li, X.; Lin, C.; Han, L.; Costello, C. E.; O'Connor, P. B. Charge Remote Fragmentation in Electron Capture and Electron Transfer Dissociation. *J. Am. Soc. Mass Spectrom.* **2010**, *21*, 646–656.

(S10) Nanayakkara, V. K.; Freiser, B. S. Thermochemistry and Photodissociation Studies of [CoL], [CoL_2], L= Pyrrole, Furan, Thiophene and Selenophene. *J. Mass Spectrom.* **1997**, *32*, 475–482.

(S11) Ouellette, J. D.; R., R. R. *Organic Chemistry Study Guide*; Elsevier: Amsterdam, 2015.

(S12) Salvatella, L. The Alkyl Group Is a -I + R Substituent. *Educ. Química* **2017**, *28*, 232–237.

(S13) Grabarz, A.; Jędrzejewska, B.; Skotnicka, A.; Murugan, N. A.; Patalas, F.; Bartkowiak, W.; Jacquemin, D.; Ośmiałowski, B. The Impact of the Heteroatom in a Five-Membered Ring on the Photophysical Properties of Difluoroborates. *Dyes Pigm.* **2019**, *170*, 107481.

(S14) Konermann, L.; Ahadi, E.; Rodriguez, A. D.; Vahidi, S. Unraveling the Mechanism of Electrospray Ionization. *Anal. Chem.* **2013**, *85*, 2–9.

(S15) Cremer, D.; Kraka, E. A Description of the Chemical Bond in Terms of Local Properties of Electron Density and Energy. *Croat. Chim. Acta* **1984**, *57*, 1259–1281.

(S16) Espinosa, E.; Molins, E.; Lecomte, C. Hydrogen bond strengths revealed by topological analyses of experimentally observed electron densities. *Chem. Phys. Lett.* **1998**, *285*, 170–173.
